# Supplementary material for: Aging activates escape of the silent X chromosome in the female mouse hippocampus
Source: Sci Adv. 2025 Mar 5;11(10):eads8169. doi: 10.1126/sciadv.ads8169 (PMC11881916; doi:10.1126/sciadv.ads8169)
Supplement: 20250305-1 [file sciadv.ads8169.v1.pdf]

WOMEN'S HEALTH

Aging activates escape of the silent X chromosome in the female mouse hippocampus

Margaret Gadek[1,2,3], Cayce K. Shaw[1,4], Samira Abdulai-Saiku[1], Rowan Saloner[1,5], Francesca Marino[1,6], Dan Wang[1], Luke W. Bonham[5], Jennifer S. Yokoyama[1,5,7], Barbara Panning[8], Bérénice A. Benayoun[9,10,11,12], Kaitlin B. Casaletto[1,5], Vijay Ramani[8,13,14], Dena B. Dubal[1,3,4,6,15]*

Copyright © 2025 The Authors, some rights reserved; exclusive licensee American Association for the Advancement of Science. No claim to original U.S. Government Works. Distributed under a Creative Commons Attribution License 4.0 (CC BY).

Women live longer than men and exhibit less cognitive aging. The X chromosome contributes to sex differences, as females harbor an inactive X (Xi) and active X (Xa), in contrast to males with only an Xa. Thus, reactivation of silent Xi genes may contribute to sex differences. We use allele-specific, single-nucleus RNA sequencing to show that aging remodels transcription of the Xi and Xa across hippocampal cell types. Aging preferentially changed gene expression on the X's relative to autosomes. Select genes on the Xi underwent activation, with new escape across cells including in the dentate gyrus, critical to learning and memory. Expression of the Xi escapee *Plp1*, a myelin component, was increased in the aging hippocampus of female mice and parahippocampus of women. AAV-mediated *Plp1* elevation in the dentate gyrus of aging male and female mice improved cognition. Understanding how the Xi may confer female advantage could lead to novel targets that counter brain aging and disease in both sexes.

INTRODUCTION

The expansion of aging research to investigate female-specific biology and its mechanistic underpinnings represents a major advance of high importance for human health. True sex differences exist in aging, and understanding what makes women more resilient (or vulnerable) reveals targets for therapeutic paths that may benefit women's health, men's health, or both (*1*).

Women live longer than men, across socioeconomic status, despite famines and epidemics (*2*), and all around the world (*2*, *3*), suggesting a common biologic contribution to female longevity. Female survival advantage is also variably observed in the animal kingdom (*4*–*7*) and extends to many (*8*–*10*), but not all (*11*), strains of mice, including those that are genetically heterogeneous (*9*). Sex chromosomes affect longevity (*10*) and may also affect resilience in health span, or the span of time spent healthy for specified outcomes. While female health span differs in its resilience and vulnerabilities across the body (*12*, *13*), the connection between the number of X chromosomes (X's) and longevity raises the question of whether X's also affect cognitive resilience.

[1]Department of Neurology and Weill Institute for Neurosciences, University of California, San Francisco, San Francisco, CA, USA. [2]Medical Scientist Training Program, University of California, San Francisco, San Francisco, CA, USA. [3]Biomedical Sciences Graduate Program, University of California, San Francisco, San Francisco, CA, USA. [4]Rehabilitation Sciences Graduate Program, University of California, San Francisco, San Francisco, CA, USA. [5]Memory and Aging Center, University of California, San Francisco, San Francisco, CA, USA. [6]Neurosciences Graduate Program, University of California, San Francisco, San Francisco, CA, USA. [7]Department of Radiology and Biomedical Imaging, University of California, San Francisco, San Francisco, CA, USA. [8]Department of Biochemistry and Biophysics, University of California, San Francisco, San Francisco, CA, USA. [9]Leonard Davis School of Gerontology, University of Southern California, Los Angeles, CA, USA. [10]Molecular and Computational Biology Department, USC Dornsife College of Letters, Arts and Sciences, Los Angeles, CA, USA. [11]Biochemistry and Molecular Medicine Department, USC Keck School of Medicine; USC Norris Comprehensive Cancer Center, Los Angeles, CA, USA. [12]USC Stem Cell Initiative, Los Angeles, CA, USA. [13]Gladstone Institute for Data Science and Biotechnology, J. David Gladstone Institutes, San Francisco, CA, USA. [14]Bakar Computational Health Sciences Institute, San Francisco, CA, USA.
[15]Bakar Aging Research Institute, University of California, San Francisco, San Francisco, CA, USA.
*Corresponding author. Email: dena.dubal@ucsf.edu

An increasing body of evidence supports female resilience in brain aging. Women undergo slower molecular brain aging, measured by the epigenetic clock, across brain regions (*14*), compared to men. Furthermore, women harbor a younger metabolic brain age, measured by positron emission tomography imaging (*15*). These findings could underlie the striking observation that women show resilience to cognitive decline (*16*–*20*) and exhibit higher baseline memory functioning in typical aging of several populations, in the absence of dementia (*16*–*18*, *20*, *21*). Etiologies of female-based cognitive resilience, also observed in aging mice (*22*), highlight a role for the second X. In normal aging (*22*), and in models of Alzheimer's disease (AD) (*23*), adding a second X improves cognition in male mice and subtracting it worsens cognition in female mice (*22*, *23*), indicating a causal role for the second X in cognitive resilience.

Enriched for neural factors, the X harbors 5% of our genome and has been largely understudied in the aging brain. In female mammals with two X's, one is silenced through X chromosome inactivation (XCI), resulting in an active (Xa) and inactive X (Xi) (*24*). While XCI is thought to equalize X-linked gene expression between XX and XY cells, select genes from the Xi escape inactivation, to varying degrees and in a tissue-specific manner (*25*, *26*). Since escape results in expression from both the Xa and Xi (*25*), a second X selectively increases X-linked gene dose in females and could potentially drive sex bias in cognitive resilience. Whether Xi escape remains unchanged into old age and if aging can affect Xi expression in the female brain are fascinating and unresolved questions. Furthermore, whether Xi expression uniquely and heterogeneously manifests across cell types in a key area of learning and memory targeted by aging, such as the hippocampus, is unknown.

Here, we investigated whether aging modulates expression of genes on the Xi, or the silent X, in the female hippocampus using an approach combining single-nucleus RNA sequencing (snRNA-seq) with allele-specific analysis, thus enabling discrimination of Xi from Xa expression in a cell type–specific manner. We report that aging preferentially modulates differential gene expression on the X compared to autosomes, with a near-global up-regulation of genes from both the Xa and Xi, in a cell type–specific manner. Focused analysis of the Xi revealed many

changes including its cell type–specific activation of many genes, with increased baseline escape and new age-induced escape. Further investigation of a robust age-induced escape factor suggested that changes in Xi expression contribute to cognitive resilience in the aging female brain.

RESULTS

Genetic model of nonrandom XCI to detect escapees from Xi

To define and distinguish expression from the Xi and the Xa, we used a genetic model that combines two mouse strains, enabling strain-specific analysis based on differing alleles on the two X's. Combining mouse strains is commonly used to study allele-specific expression (*25*, *27*–*29*). In this model, one X is inherited from *Mus musculus* and the other from *Mus castaneus* (Fig. 1A), which exhibits single-nucleotide polymorphisms (SNPs) roughly every 264 base pairs (bp). Thus, transcripts can be mapped to either the *M. musculus* or *M. castaneus* genome.

XCI is random (Fig. 1A, bottom left) such that one X is the Xa in approximately half of cells and the other X is the Xa in the remaining half (*24*), which adds analytical complexity to single-cell studies. To simplify analysis, we implemented a genetic model of nonrandom XCI, with the Xa coming from *M. musculus* and the Xi coming from *M. castaneus* (Fig. 1A, bottom right) (*25*). In this model, the *M. musculus* X harbors a deletion of *Xist*, which encodes a long noncoding RNA required in cis for XCI (*30*). Since only the *M. castaneus* X can transcribe *Xist* during development, it is always the Xi in our mouse model. Thus,

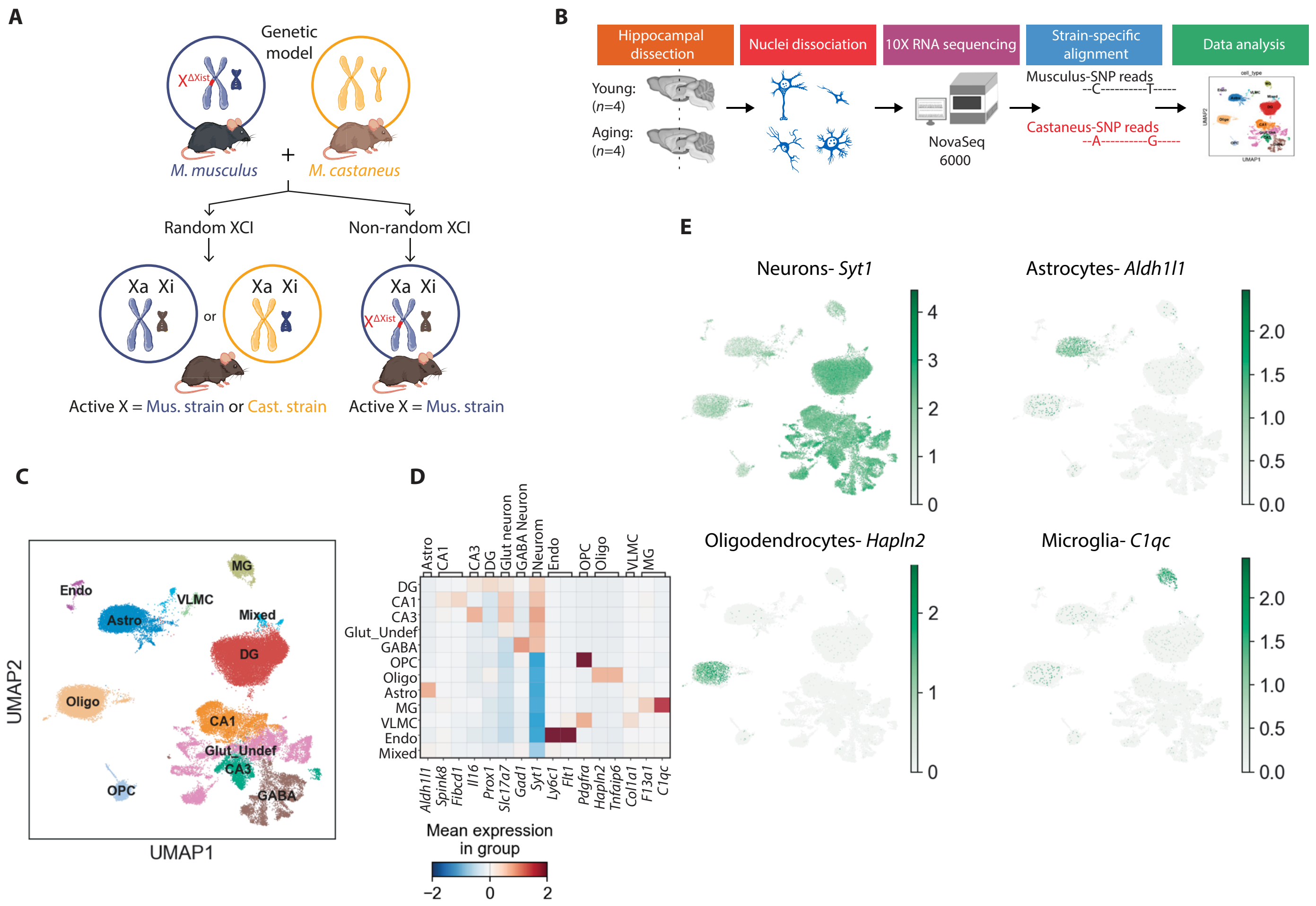

Fig. 1. Allele-specific, single-nucleus sequencing of cell types in the young and aging XX hippocampus. (**A**) Genetic model for allele-specific investigation of the X. *M. musculus* XX mice with an *Xist* deletion were crossed with XY *M. castaneus* mice. XX progeny without an *Xist* deletion (left) underwent random XCI. XX progeny with an *Xist* deletion (right) harbor an Xa chromosome strictly from the *M. musculus* strain and an Xi chromosome from the *M. castaneus*. Differences in SNPs between the strains enable a direct measure of expression from each X, the Xa and the Xi. Image credit: D. Velasco. (**B**) Diagram of the experimental workflow of allele-specific, single-nucleus sequencing. Young (*n* = 4 mice, age = 3 months) and old (*n* = 4 mice, age = 22 months) hippocampi from the strain-specific X-detection mice were dissected. Nuclei were dissociated and sequenced with 10x RNA sequencing on the Illumina NovaSeq 6000. Reads were aligned to the combined *M. musculus* and *M. castaneus* genome and analyzed by origin from Xa or Xi, cell type, and age. Hippocampi, cells, and NovaSeq were acquired with a ShutterStock Enhanced License. (**C**) Hippocampal cell types sequenced. Dimension reduction analysis using UMAP revealed 11 distinct cell type populations sequenced plus a mixed category. Clusters are the following: endothelial cells (Endo), astrocytes (Astro), vascular leptomeningial cells (VLMC), microglia (MG), DG neurons, CA1 neurons, glutamatergic undefined neurons (Glut_Undef), CA3 neurons, GABAergic neurons (GABA), oligodendrocyte progenitor cells (OPCs), and oligodendrocytes (Oligo). (**D**) Matrix plot showing expression relating cell type marker genes (columns), cell clusters (rows), and corresponding genes. (**E**) Heatmap of cell markers across UMAP clusters. Color intensity reflects extent of gene expression.

transcripts with SNPs mapping to the *M. castaneus* genome arise from the Xi and are designated escapees.

Using snRNA-seq, we profiled over 40,000 nuclei from four young and four old female mouse hippocampi (Fig. 1B). Nuclei were isolated from dissected hippocampi, with similar numbers of nuclei per sample, processed through droplet-based single-cell profiling, and sequenced. The reads were assigned to the *M. musculus* or *M. castaneus* genome for further analysis. Similar median unique molecular identifier (UMI) counts and gene counts were assigned to each sample (data S1). Autosomal reads were 56% *M. musculus* and 44% *M. castaneus*, with a mild mapping bias toward *M. musculus*, as observed in other allele-specific sequencing studies (*25*). In contrast, reads arising from the X were 91.7% *M. musculus* and 8.3% *M. castaneus* (including robust *Xist* expression), consistent with silencing of the *M. castaneus* X, and with the estimated 3 to 7% of escape in mice (*25*). These findings validate the genetic model of establishing nonrandom XCI to specifically enable the detection of escapees from the Xi.

Single-nucleus sequencing of the young and old female mouse hippocampus

Nuclei clustered into different cell types using the Leiden algorithm in Scanpy (Fig. 1C) (*31*). Using a comparison to the Allen Brain Atlas 10x hippocampus and cortex datasets, differentially expressed genes (DEGs) were assigned to corresponding groups (data S2) (*32*). Clusters with similar cell marker identities were combined, and cell types were assigned using the most DEGs in the cluster compared to all others. Neuronal cell types included glutamatergic neurons such as CA1, CA3, and dentate gyrus (DG) neurons. Other undefined glutamatergic neurons (Glut_Undef) and GABAergic (GABA) neurons were also present. Glial cell types included astrocytes (Astro), oligodendrocytes (Oligo), oligodendrocyte progenitor cells (OPC), and microglia (MG). Finally, vascular leptomeningeal cells (VLMC) and endothelial cells (Endo) were identified. Transcriptionally indistinct mixed nuclei made up 0.9% of the nuclei population. The distinctly identified clusters were further validated with known marker genes from other mouse brain single-cell datasets (Fig. 1, D and E) (*32*–*35*). Expression of marker genes across clusters showed expected patterns. Thus, the cell clustering analysis of our snRNA-seq dataset reflected the spectrum of cell types typically present in the hippocampus, indicating a robust model to probe gene expression across hippocampal cell types in aging.

Enrichment of DEGs on the X in aging hippocampus

We first conducted a differential gene expression analysis of combined cell types and individual cell types. Representation of hippocampal neuronal and glial cell types was similar in young and old cell populations and across samples (fig. S1) (data S3). Using the decoupler and pyDEseq2 programs (*36*, *37*), we interrogated aging-induced differential gene expression via pseudobulking expression by cell type and sample. In combined cell types, 926 genes were significantly differentially expressed in old compared to young nuclei (Fig. 2A) (data S4). Approximately 50% of these DEGs were previously identified to change in similar directions in datasets of the aging male (*38*) and female (*33*) mouse brain (Fig. 2A, top hits in red) (data S5). Also in the combined cell types, among the 926 DEGs, 29 were from the X (Fig. 2A, in blue).

As global changes to X regulation were previously identified as a feature of female hypothalamic aging (*33*), we wondered whether aging preferentially alters DEGs on the X compared to autosomes in the hippocampus. When normalizing for total genes of each chromosome, more genes changed on the X (Xa and Xi combined) than on autosomes (Fig. 2B). This finding extended to analyses of individual autosomes of similar size compared to the X (fig. S2). DEG overrepresentation on the X similarly extended to most cell types in the aging, compared to young, hippocampus (Fig. 2C). That is, similar to the combined all-cell population, CA1 neurons, CA3 neurons, DG neurons, GABAergic neurons, microglia, and oligodendrocytes individually showed an increased number of significant DEGs from the X, compared to autosomes. Notably, DG neuronal nuclei harbored the highest number of significant DEGs, from both autosomes (14%) and X's (20%), followed by Oligos (Fig. 2C), suggesting that they may be particularly sensitive to age-induced changes on the X.

Following the finding that aging-induced DEGs were enriched on the X, we next assessed cell type–specific RNA expression of X-linked genes, applying genome-wide corrections. First, using g:Profiler, we conducted a gene ontology (GO) term analysis of the X-linked genes significantly enriched in each cell type compared to other cell types in our dataset (*39*). The top predicted structures and functions across cell types were the synapse and synaptic organization (Fig. 2D), consistent with the high proportion of genes related to cognition on the X (*40*). Thus, the enrichment of neural functions on the X highlights the high value for its study in the hippocampus and for cognitive aging.

Aging-induced DEGs from Xa and Xi across hippocampal cell types

We assessed significant X-linked DEGs, for those broadly shared across cell types like neurons and glia, and for cell type–specific populations. DEGs, measured from the Xa or Xi, were classified as neuronal, glial, multiple, or cell type specific according to inclusion criteria (Fig. 2E). Aging nearly globally resulted in up-regulated neuronal and glial DEGs—and largely up-regulated multiple and cell type–specific DEGs—from both the Xa and Xi (Fig. 2E). Notably, most X-linked DEGs changed in a cell type–specific manner, highlighting the heterogeneity of aging effects within the hippocampus. Even so, the convergent aging-induced increase in X-linked expression across cell types suggests common chromatin regulators and transcription factors, and less transcriptional repression, across the X's.

To investigate how aging alters predicted cellular components and functions referable to the X, we performed individual GO term enrichment analyses of significant X-linked DEGs that change with aging for each cell type (fig. S3). GO terms related to synaptic regulation, activity, and organization were the most robust and remarkably conserved pathways among nearly all cell types (fig. S3). Cell type–specific differences also emerged, such as GTPase (guanosine triphosphatase) binding (CA3, DG, Oligos) or constituents of the myelin sheath (Astro and Oligos) (fig. S3). Rho GTPase signaling integrates synaptic structure and function through AMPA receptor trafficking and represents a treatment avenue for cognitive disorders (*41*, *42*), while myelination decreases with aging and contributes to cognitive decline (*43*). Since synapses and myelin are fundamental units and substrates of neural function, and targets of aging-induced dysfunction, these enrichments collectively suggest a molecular basis for XX-based resilience against cognitive decline in aging. Details of aging-induced X-linked DEGs that were most common to neuronal and glial cell populations are reported in Supplementary Text.

We identified the aging-induced DEGs derived from Xa, present in the most cell types; all notably harbor mutations in humans that

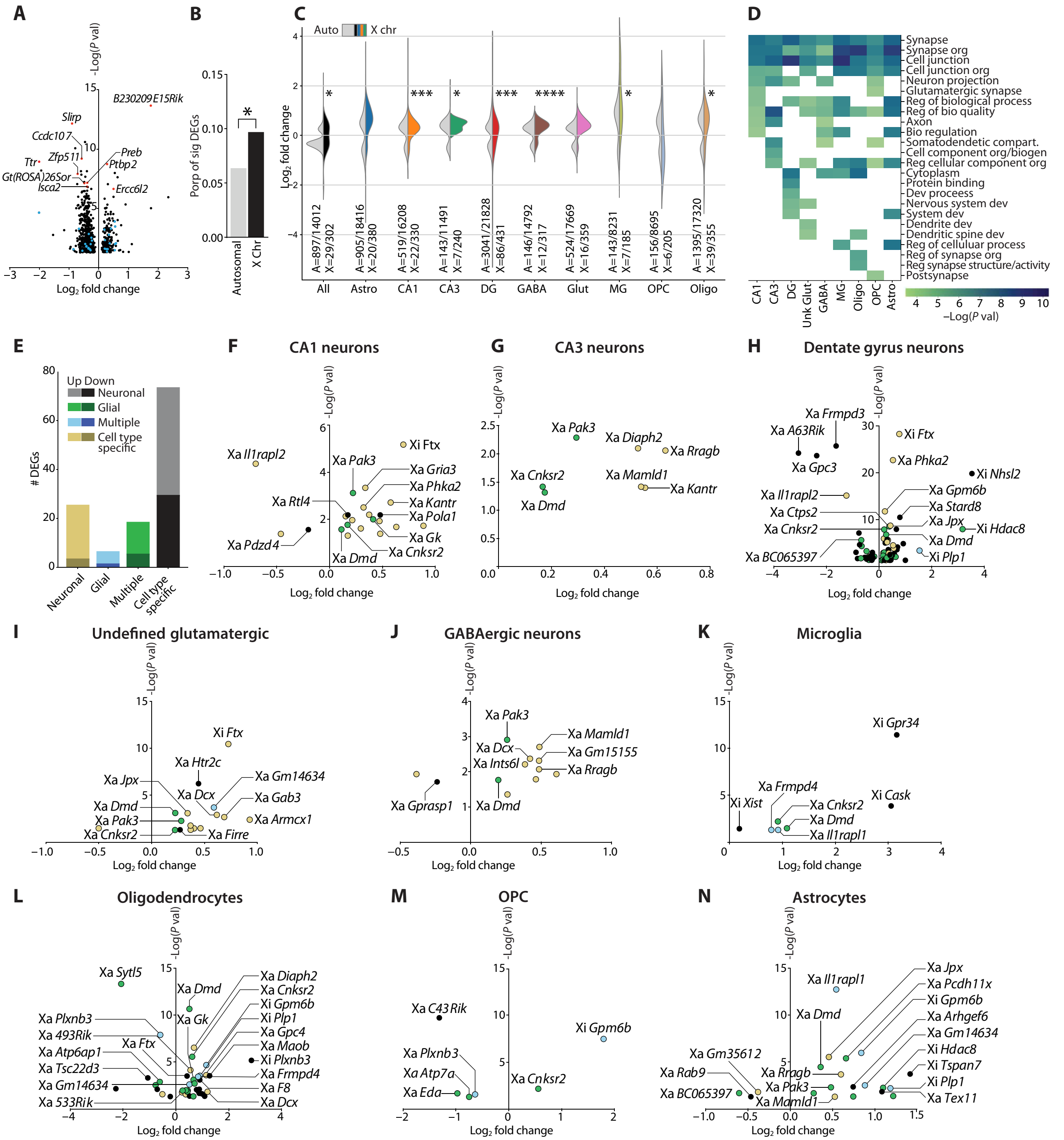

Fig. 2. Aging induces differential gene expression in the hippocampus across autosomes and the X's. (**A**) Aging significantly altered 926 DEGs ($\log_2$ fold change > 0.1 or < −0.1 and adjusted $P < 0.05$) in the hippocampus, including 29 expressed from the Xa and Xi in blue, when considering all cell types. Top 10 DEGs matching the described published datasets are in red. (**B**) DEGs from the autosome and X's, normalized to total expressed genes (*$P = 0.025$, χ^2 test). (**C**) Violin plot showing differentially expressed alleles by cell type and $\log_2$ fold change. Distributions of significant autosomal DEGs (light gray, left of violin plot) and distributions of significant X DEGs (color, right of violin plot) are shown. Proportion of significant autosomal (A) and X (X) genes over the total detected genes are shown comparing autosome and X significant DEG proportion (χ^2 tests: All *$P = 0.0253$, Astro ns, CA1 ***$P = 0.0005$, CA3 *$P = 0.0225$, DG ***$P = 0.0004$, GABA ****$P < 0.0001$, Glut ns, MG *$P = 0.0375$, OPC ns, Oligo *$P = 0.0452$. (**D**) Top 10 GO terms (rows) from X genes significantly expressed in each cell type by −log(adjusted P value). (**E**) Number of DEGs up- and down-regulated in the cell type–specific volcano plots in (F) to (N). Classifications include the following: Neuronal, ≥2/5 neuronal cell types ≤1 glial; Glial, ≥2/4 glial cell types ≤1 neuronal genes; Multiple, ≥2 cell types; Cell type–specific, 1 cell type only. Two genes in the multiple cell types category, Xa *Pcdh11x* and Xa *Sytl5*, were excluded because they increased in one cell type but decreased in another. (**F** to **N**) Volcano plots of significant DEGs on Xa and Xi in cell types including (F) CA1 neurons, (G) CA3 neurons, (H) DG neurons, (I) undefined glutamatergic neurons, (J) GABAergic neurons, (K) microglia, (L) oligodendrocytes, (M) oligodendrocyte progenitor cells (OPC), and (N) astrocytes. *A630012P03Rik*, *C430049B03Rik*, *4930500G05Rik*, and *5330434G04Rik* are abbreviated as *A63Rik*, *C43Rik*, *493Rik*, and *533Rik*, respectively. Image credit: E. Kakoshina.

cause intellectual disability: *Dmd* (*44*, *45*), *Cnksr2* (*46*, *47*), and *Pak3* (*48*, *49*). These X genes increased in at least seven cell types (Fig. 2, F to N) and have known hippocampal functions. *Dmd* encodes dystrophin, a scaffold protein at inhibitory synapses (*50*). *Cnksr2* is also a synaptic scaffolding protein for granule cells of the DG (*51*). *Pak3* is a synaptic connectivity protein that forms spines (*52*) and synapses (*53*), and contributes to cognition (*54*). Thus, synaptic functions of aging-induced Xa genes are prominent and show human relevance as demonstrated by intellectual disability resulting from their loss of function or pathogenic variation.

The aging-induced DEGs derived from the Xi represented in most cell types were *Ftx*, *Gpm6b,* and *Plp1*. These genes increased on the Xi in three to four cell types that were either neuronal (*Ftx*) or glial (*Gpm6b*, *Plp1*) biased (Fig. 2, F to N). *Ftx*, the long noncoding RNA, inhibits hippocampal apoptosis (*55*) and ferroptosis (*56*). Also, as a positive regulator of *Xist* (*57*), its age-induced increase might influence XCI regulation. Members of the proteolipid protein family, *Gpm6b* and *Plp1* (*58*), both contribute to myelination, and human mutations in *PLP1* cause hypomyelination and intellectual disability (*59*). Since defects in myelin maintenance contribute to age-related cognitive decline (*43*), it is interesting to speculate that modest increases in these factors from the Xi could attenuate cognitive deficits.

Since snRNA-seq provides a shallow depth of coverage and allele-specific analysis requires doubling the genes detected (one from each allele), together they necessarily constrain both coverage and statistical power. Thus, cell type–specific DEG analyses yield high specificity but low sensitivity for detecting gene changes, particularly from genes on the Xi, which are expressed at lower levels than on the Xa. Given our focus on detecting X escape from XCI, we complemented our study with additional analyses.

Xi escape from XCI, with reference to Xa, in the aging hippocampus

We next implemented a well-established and conservative analysis pipeline (*25*) to define X escape and assess how Xi expression changes across cell types and age, in reference to Xa. This better powered and more sensitive computational approach (Fig. 3A), compared to single-nucleus DEG analysis, aggregates reads broadly across and within cell types (pseudobulking) and enables a quantitative detection of escape from XCI (data S6). X escape status was calculated using an escape proportion and its corresponding 99% confidence intervals (CIs). To meet criteria as an escapee, a lower bound of the CI must be greater than 0, and the median escape value (the escape proportion) must be greater than 0.05 (Fig. 3A).

Baseline escape genes were identified as those that showed escape in the young life stage, and differences between escapee behavior in young and old cohorts were categorized. Genes exhibited increased escape from baseline if they escaped in both young and old samples, but the lower bound of their old CI was greater than the upper bound of their young CI. Baseline escapees increased (Fig. 3B, purple), lost (Fig. 3B, maroon), or maintained (Fig. 3B, red) their level of expression with age. In addition, aging activated expression of previously silent Xi-linked genes to induce their new escape (Fig. 3B, orange).

With this framework for Xi escape across aging in place, we examined escape in a population of combined cell types (Fig. 3B). Of the 73 baseline escapees identified, 4 increased expression with age, 51 maintained expression, and 18 lost expression with aging. Remarkably, aging induced new expression of 19 genes from Xi that were previously inactive in the young life stage. Of particular interest were age-induced (orange) and increased escape with age (purple) genes (Fig. 3C), since their Xi expression was increased in hippocampal cells (Fig. 3, D to L).

We next determined whether there was cell type variation in how aging affected Xi-linked gene expression (Fig. 3, D to L, and fig. S4, A to D). In the DG and glutamatergic neurons, aging both robustly activated and repressed Xi-linked genes, indicating dynamic age-induced remodeling of fundamental neural units underlying learning and memory. In comparison, in CA1 and CA3 (which receive and refine DG input), GABAergic cells, and OPCs, aging predominantly repressed Xi-linked gene expression. In contrast, aging markedly activated Xi-linked gene expression in microglia, astrocytes, and oligodendrocytes, potentially influencing neuroimmune-mediated functions and proper myelination. Thus, the effects of aging on the Xi varied by cell type, highlighting the complexity of gene regulation of the highly and structurally condensed Xi.

To maintain XCI-mediated transcriptional silencing, the Xi is tightly compacted by heterochromatin, is coated by Xist RNA, and exhibits altered DNA and histone methylation patterns compared to the Xa. Since aging altered expression of Xi-linked genes, we wondered whether it influences regulators of XCI. While *Xist* was abundant in every cell, as anticipated, a further increase in already high expression was only detected on Xi in microglia (Fig. 2K). Because *Xist* deletion from Xa was used to enforce nonrandom XCI, our method does not capture any *Xist* expression from the Xa, which may explain why we do not observe the previously reported increase in abundance of this noncoding RNA in several wild-type hippocampal cell types (*33*). Nonetheless, and interestingly, aging changed expression of other XCI regulators from the Xi, including *Jpx* and *Ftx*. Aging increased *Jpx* expression from the Xi in glutamatergic neurons (Fig. 3G) and decreased it in CA3 neurons (fig. S4) Furthermore, aging consistently increased Xi expression of *Ftx* in CA1, DG, and glutamatergic neurons (Fig. 3, D, F, and G, and fig. S4), key cell types for learning and memory in the hippocampus. Our results converge with previous findings that aging also increased total *Jpx* and *Ftx* expression in the female hypothalamus (*33*); our data suggest that this increase was from Xi. Whether these XCI regulators affect the XCI status of Xi, or whether they exert XCI-independent functions in the aging hippocampus, remains to be determined.

We examined whether the same genes could be targets of aging-induced Xi modulation across multiple cell types of the hippocampus. Approximately half (44%) of Xi-mediated changes in a gene's expression extended across two to eight cell types in the given escape pattern. For example, aging increased *Plp1* expression on the Xi consistently across seven cell types (CA1 neurons, DG neurons, undefined glutamatergic neurons, GABAergic neurons, oligodendrocytes, microglia, and astrocytes) (Fig. 3, D, F to J, and L, and fig. S4). Relatedly, aging increased expression of *Gpm6b* in glial cells (oligodendrocytes, OPCs, and astrocytes), induced new escape in MG, and decreased expression in neuronal cells (CA1, CA3, and undefined glutamatergic neurons) (Fig. 3, D, E, G, and J to L, and fig. S4). Thus, as top examples, *Plp1* and *Gpm6b* were targets of Xi modulation in aging, each in seven of nine cell types, suggesting their heightened accessibility to activation or repression on the silent X in aging.

Cell type–specific repression and activation of the Xi

To shore up findings of Xi escape, calculated with reference to Xa, we next further examined gene changes by focusing on Xi-only expression. In addition to understanding aging-induced Xi activation through escapee expression increases and new escape with aging, we

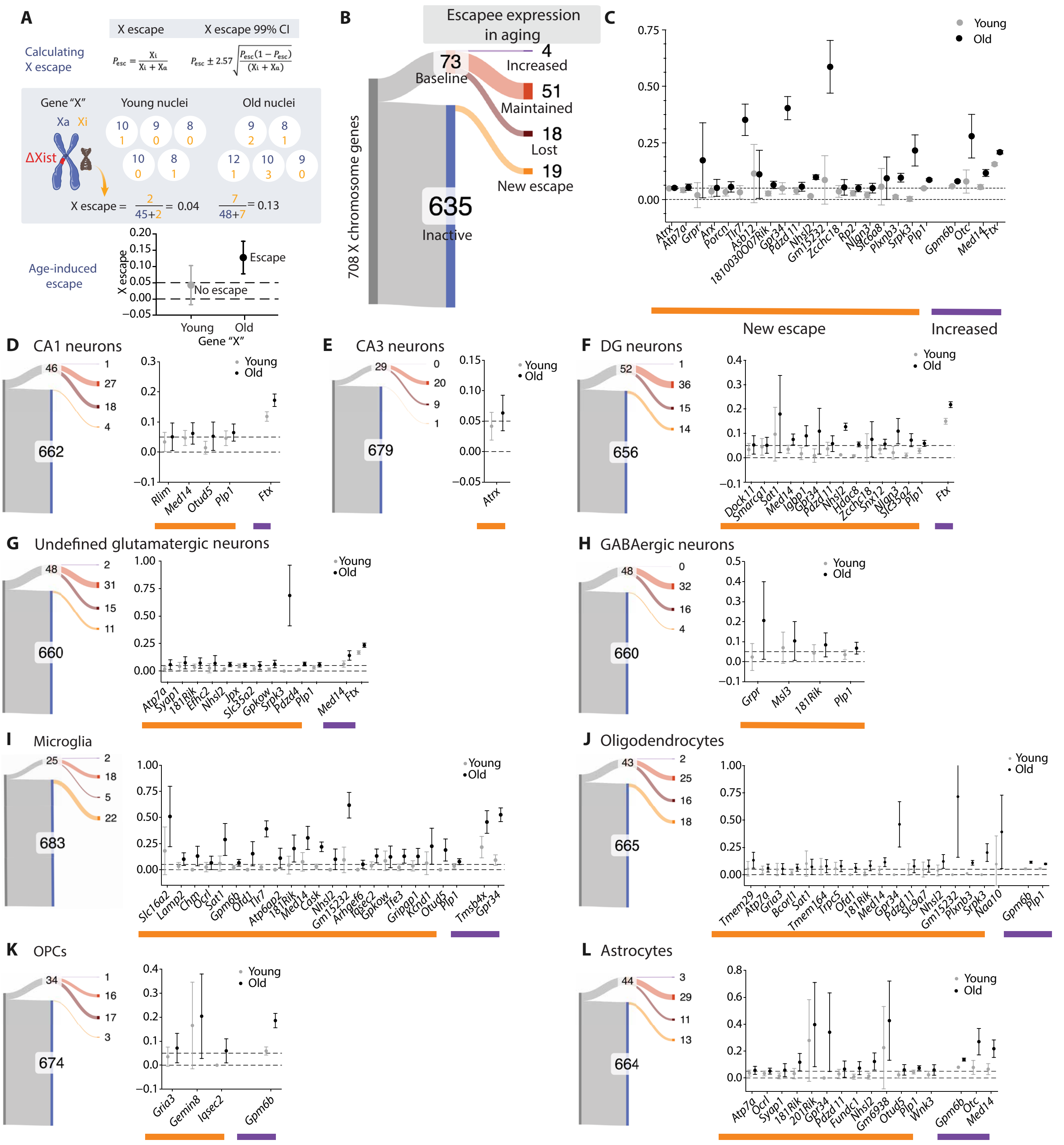

Fig. 3. Xi escape from XCI, with reference to Xa, in the aging hippocampus across cell types. (**A**) Schematic of escape ratio and CI calculation with an example calculation for hypothetical gene "X" showing age-induced escape. (**B**) Sankey plot showing the classification of X escapee expression of 708 detected X genes in young (middle) and old (right) populations for bulk cell populations. The gene expression is first classified as being baseline escapees (red) or inactive genes (blue). Escapee expression in aging is further divided into increased (purple), maintained (red), and lost escape with age (maroon). The Xi genes are further divided to note if they become new escapees (orange). (**C**) Bulk cell population, Xi escape 99% CIs of age-activated escapees (orange) and increased escape (purple). A gene is an escapee if the lower bound of its CI is greater than 0 (lower dotted line) and its median is greater than 0.05 (higher dotted line). (**D** to **L**) Sankey plots of Xi escape genes with age (left) and their escape CIs (right) in different cell types including (D) CA1 neuron, (E) CA3 neurons, (F) DG neurons, (G) undefined glutamatergic neurons, (H) GABAergic neurons, (I) Microglia, (J) Oligodendrocytes, (K) Oligodendrocyte progenitor cells (OPC), and (L) Astrocytes. *1810030O07Rik* and *2010308F09Rik* are abbreviated as *181Rik* and *201Rik*, respectively.

extended analyses to aging-induced repression by querying X genes that lost their baseline escape status with aging.

In a combined cell type analysis, escapee genes from the Xi identified to increase expression, lose expression, newly escape in aging, or fit multiple categories (mixed escape)—observed in at least two hippocampal cell types (fig. S4, B to D)—were examined for their fraction of cells and mean Xi expression in young and old mice (Fig. 4A). Broadly, aging modulated escapee expression (Fig. 4A). Notably, the escapee *Ftx* solely increased expression with aging across multiple cell types—and at very high levels. Mixed escape expression patterns showed opposing Xi changes with age in different cell types that canceled out the signal in this combined cell type analysis.

To increase resolution of Xi-only expression in aging, we next used a cell type–specific approach and queried loss of escapee expression occurring in at least two cell types (Fig. 4B and fig. S4). Aging repressed Xi through loss of escapee expression, resulting in loss of escape status, depicted by reduced gene expression and fraction of cells of the depicted cell type and life stage (Fig. 4B). Parallel

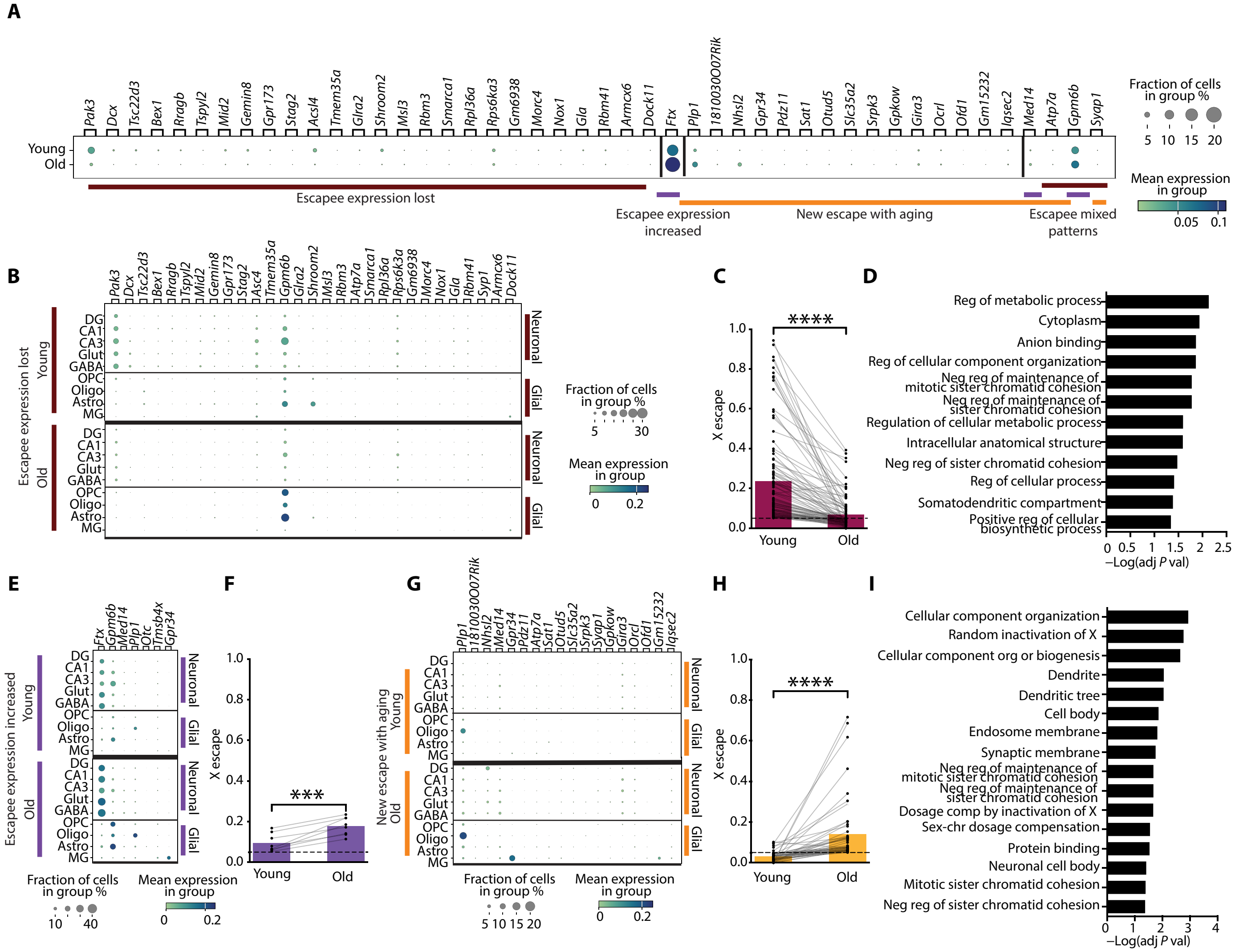

Fig. 4. Activation and repression of Xi-only expression in aging and across cell types. (**A**) Combined, bulk cell analysis of Xi expression showing genes with lost escape (maroon), escapee expression increased (purple), new escape with aging (orange), or mixed escape, observed in at least two hippocampal cell types in young and old populations. Circle color indicates expression. Circle size indicates the proportion of cells in the group expressing the gene. Image credit: D. Velasco. (**B**) Cell type–specific repression, or lost escape of Xi with age observed in at least two cell types. Young (top) and old (young) Xi expression of the escapee gene. (**C**) Median escape values of Xi genes that undergo repression, or loss of escape with age, in at least two cell types ($n = 100$ data points, representing 29 genes, each in the cell types with escapee expression lost, $****P < 0.0001$, paired two-tailed t test). (**D**) GO term analysis of Xi genes that underwent repression, or of loss of escape, in at least one cell type by −log(adjusted P value). (**E**) Cell type–specific increase in escapee expression from Xi with age in any cell type. (**F**) Median escape values of Xi genes that undergo increased escape expression with age, in at least two cell types ($n = 8$ data points, representing three genes, each in the cell types with increased escape expression with age, $***P = 0.0003$, paired two-tailed t test). (**G**) Cell type–specific Xi expression of genes that undergo new escape with aging in at least two cell types. (**H**) Median escape values of Xi genes that undergo new escape with aging, in at least two cell types ($n = 53$ data points, representing 18 genes, each in the cell types with new escape with aging, $****P < 0.0001$, paired two-tailed t test). (**I**) GO terms of combined Xi genes that underwent escapee expression increased and new escape with aging, in at least one cell type by −log(adjusted P value).

to patterns identified in escape analysis, aging repressed Xi expression and cell fraction of *Gpm6b* in neurons, and boosted them in glia (Fig. 4, B and E, and fig. S4, B to D). Notably, aging repressed *Pak3* across most cell types on the Xi (Fig. 4B and fig. S4C) and activated it on the Xa (Fig. 2). To further confirm the escapee's loss of expression, we graphed X escape for each aging-mediated Xi gene that lost escape status in a cell type. As expected, in their specific cell type, aging uniformly repressed these escapee genes (Fig. 4C).

We wondered what functions aging-mediated repression of the Xi could contribute to a hippocampal cell. A GO term analysis of these Xi genes in at least one cell type (fig. S4C) highlighted several terms, including those regulating chromosomes (Fig. 4D). A conspicuous contributor to this prediction is *Atrx*, a central player in genome stability that represses numerous regions of chromosomes and contributes to silencing of Xi in development (*60*). Thus, aging-mediated repression of the repressor *Atrx* on Xi could potentially contribute to new escape from the Xi.

Similar to our analysis of Xi repression, we conducted an analysis of Xi-only activation in aging, using the same cell type–specific approach (Fig. 4, E to H, and fig. S4, B and D). Aging activated Xi through increased escapee expression (Fig. 4, E and F) and new escape with aging (Fig. 4, G and H), depicted by augmented expression and fraction of cells in both groups of genes. As highlighted in the DEG and escape analyses, aging increased *Ftx* escape from the Xi in many cell types (Fig. 4E and fig. S4B). To further confirm this finding, we graphed X escape for each Xi-linked escapee that increased expression in a cell type. As expected, in their specific cell type, aging uniformly increased baseline expression of these escapee genes (Fig. 4F).

Several genes showed new escape with aging—indicating activation of the Xi—across many cell types. Among them, and confirming our previous analysis, *Plp1* notably exhibited new escape with aging in six cell types and increased escapee expression in the remaining (Fig. 4G and fig. S4D). To further confirm that genes inactive in the young life stage underwent new escape with aging, we graphed X escape for each Xi gene of this category in a cell type. As expected, in their specific cell type, aging robustly activated these genes—going from silent in young mice to new escapee expression in old mice (Fig. 4H).

To probe putative functions conferred by aging-mediated activation of Xi (increased escapee expression and new escape with aging) in the hippocampus, we performed a GO term analysis of this class with genes changed in at least one or more cell type (fig. S4, B and D). An enrichment of terms related to XCI, such as *Ftx*, *Jpx*, and *Rlim* (*57*, *61*, *62*), suggests that aging-induced Xi activation could alter its transcriptional landscape. This probably contributes to other terms enriching for broad neural functions (synaptic and endosomal membranes, neuronal cell body, and protein binding) (Fig. 4I). *Atrx* was implicated in multiple GO terms relating to chromosomal regulation, indicating that the repressor *Atrx* is repressed in some cell types and activated in others (*60*). Other escapees, *Plp1* and *Gpm6b*, are relevant to the cellular component organization term, as components of myelin and the membrane of many central nervous system cells (*63*, *64*). Finally, *Atp7a* and *Gria3* are known to contribute synaptic integrity, signaling, and neuronal activation (*65*, *66*). Thus, aging-mediated activation of the Xi may set off a cascade of transcriptional escape to ultimately alter neuronal and glial functions.

Human relevance of DEG and X escape analysis findings

The X is enriched for genes with neural functions, and the findings of our computational analyses highlighted broad neural functions alongside synaptic signaling and neural structural components. Thus, we wondered whether specific aging-induced changes in Xi-linked gene expression are enriched for genes represented among human, X-linked conditions of intellectual disability. In other words, if the select Xi genes identified here were particularly important to cognitive functions, then their mutations would predict cognitive dysfunction. To that end, we systematically searched the literature for the 100 Xi genes that exhibited age-induced changes in at least one or more cell types. Mutation of nearly half (49%) of these genes caused intellectual disability, typically in males since they lack a compensatory X (table S1) (*67*–*115*). This finding highlights the exceptionally important role of the silent X, changed by aging, in potentially influencing underlying substrates of cognitive function, one of our most valued outputs of brain function.

Female sex increases *Plp1* expression in the hippocampus of mice and parahippocampus of humans

Aging selectively activated Xi genes in both single-nucleus differential gene expression and X escape analyses, and among them, *Plp1* either was highly activated from Xi or showed increased escapee expression across cell types. *PLP1* is also implicated in Pelizaeus-Merzbacher disease, a leukodystrophy that causes intellectual disability, among other neurological deficits; thus, it has high relevance for human cognition (*81*, *115*). We therefore focused further studies on *Plp1*. Aging increased Xi expression of *Plp1* in DG neurons, oligodendrocytes, and astrocytes in the DEG analysis (Fig. 2, H, L, and N). The *Plp1* escapee ratio was highest (Fig. 5A), with the most robust Xi expression (Fig. 5B) in old oligodendrocytes. As PLP1 is intrinsic to oligodendrocyte structure and function, its strong increase from the Xi with aging suggests that it may enhance aging oligodendrocytes (*58*).

We further measured *Plp1* in mice and *PLP1* in humans. To confirm the aging-mediated increase in *Plp1* across cell types in XX mice, we compared its bulk expression in young compared to old mice in the XX and XY hippocampus (Fig. 5C) using a separate cohort. Using quantitative polymerase chain reaction (qPCR), we found that aging increased *Plp1* mRNA in XX, but not XY, mice. To assess whether this sex difference in *Plp1* extends to humans, we then assessed its expression in the parahippocampus of aging humans using the Mount Sinai Brain Bank (MSBB) cohort. This region surrounds the hippocampus and is involved in spatial memory, information, and context. Sequenced samples were analyzed and normalized for library size, and adjusted for relevant covariates, including batch, age at death, race, postmortem interval, RNA integrity number (RIN), exonic rate, and ribosomal RNA (rRNA) rate. In parallel to mice, older women showed increased *PLP1* expression in the parahippocampus, compared to older men (Fig. 5D). Increased *PLP1* in women was restricted to the parahippocampal region and not observed in the frontal pole, superior temporal cortex, or inferior frontal gyrus (fig. S5). Together, these findings confirm increased *Plp1*/*PLP1* expression in the hippocampus or its surrounding area of aging females in both mice and humans.

AAV-mediated *Plp1* overexpression in oligodendrocytes of the mouse hippocampus

To investigate the effects of increased *Plp1* on cognition in aging, we constructed an adeno-associated virus (AAV) to overexpress *Plp1*. We focused on oligodendrocytes because the highest overall expression and most robust aging-induced increase in *Plp1* was observed in this cell type. We used an AAV2/5 *Plp1* mRNA construct driven by *Mbp*, an oligodendrocyte-specific promoter, followed by a ubiquitous

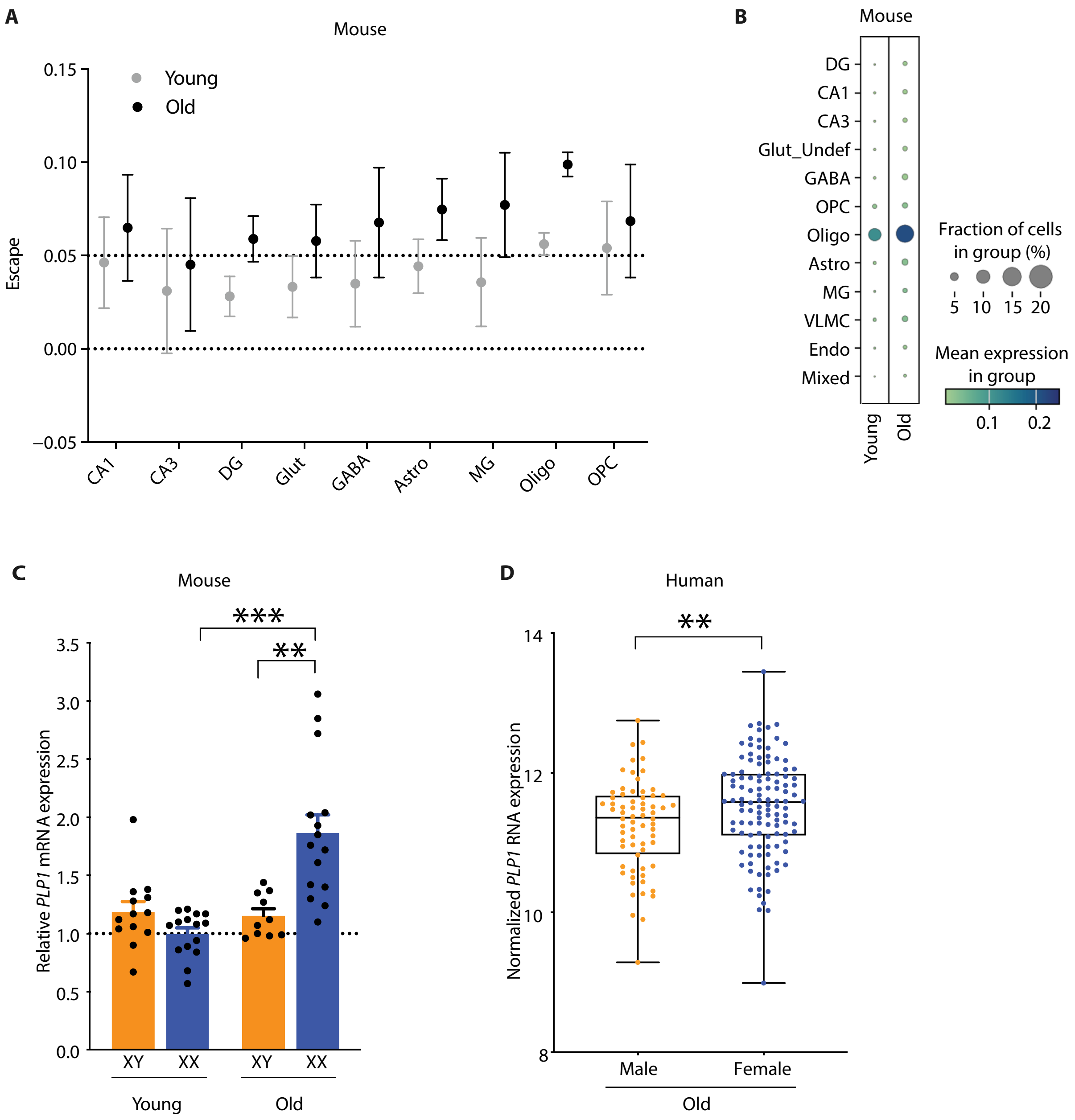

Fig. 5. Activation of hippocampal *Plp1* Xi expression across cell types in XX mice and increased *Plp1* in aging female mice and humans. (**A**) *Plp1* X-escape 99% CIs by cell type and age in XX mouse hippocampi. CIs with lower bounds above 0 (lower dotted line), with medians above 0.05 (upper dotted line), show escape from baseline Xi *Plp1* expression. (**B**) Mean expression of Xi *Plp1* in young and old hippocampal cell types. The fraction of cells in the group expressing *Plp1* correlates with circle size. (**C**) Relative *Plp1* mRNA expression in the whole hippocampus of young (age 3 months; XY, $n = 13$; XX, $n = 15$) and old (age 20 to 35 months; XY, $n = 10$; XX, $n = 15$) XY and XX mice. Means are relative to XX young mice, arbitrarily defined as 1. Old XX compared to young XX mice (***$P = 0.0002$, Bonferroni-corrected unpaired two-tailed t test). Old XX compared to old XY mice (**$P = 0.0014$, Bonferroni-corrected unpaired two-tailed t test). (**D**) Relative *PLP1* mRNA expression in the parahippocampal gyrus from postmortem tissue of old men and women (XY = 68, XX = 116). Data were normalized, $\log_2$-transformed, and adjusted to account for relevant covariates including batch, age at death, race, postmortem interval, RIN, exonic rate, and rRNA rate. Box plots represent the median and 25th and 75th percentiles, and box hinges represent the interquartile range of the two middle quartiles within a group. Min and max data points define the extent of whiskers (error bars) (**$P = 0.008$).

SV40 promoter transcribing green fluorescent protein (GFP) to mark successful transfection (Fig. 6A). The control AAV was identical to the *Plp1*-OE AAV, except for the deletion of *Plp1*. In vitro infection of a mixed glial population including oligodendrocytes confirmed that *Plp1*-OE compared to control AAV increased *Plp1* mRNA overexpression in both XY and XX cells (Fig. 6, B and C).

Following in vitro validation, we performed in vivo AAV studies in aging XY and XX mice. We specifically injected the control and *Plp1*-OE AAV into the DG of the mouse hippocampus to investigate whether *Plp1* mediates behavioral changes in the aging brain (Fig. 6D). We focused on the DG since it is a key region in cognitive functions like spatial memory (*116*) and also harbored the

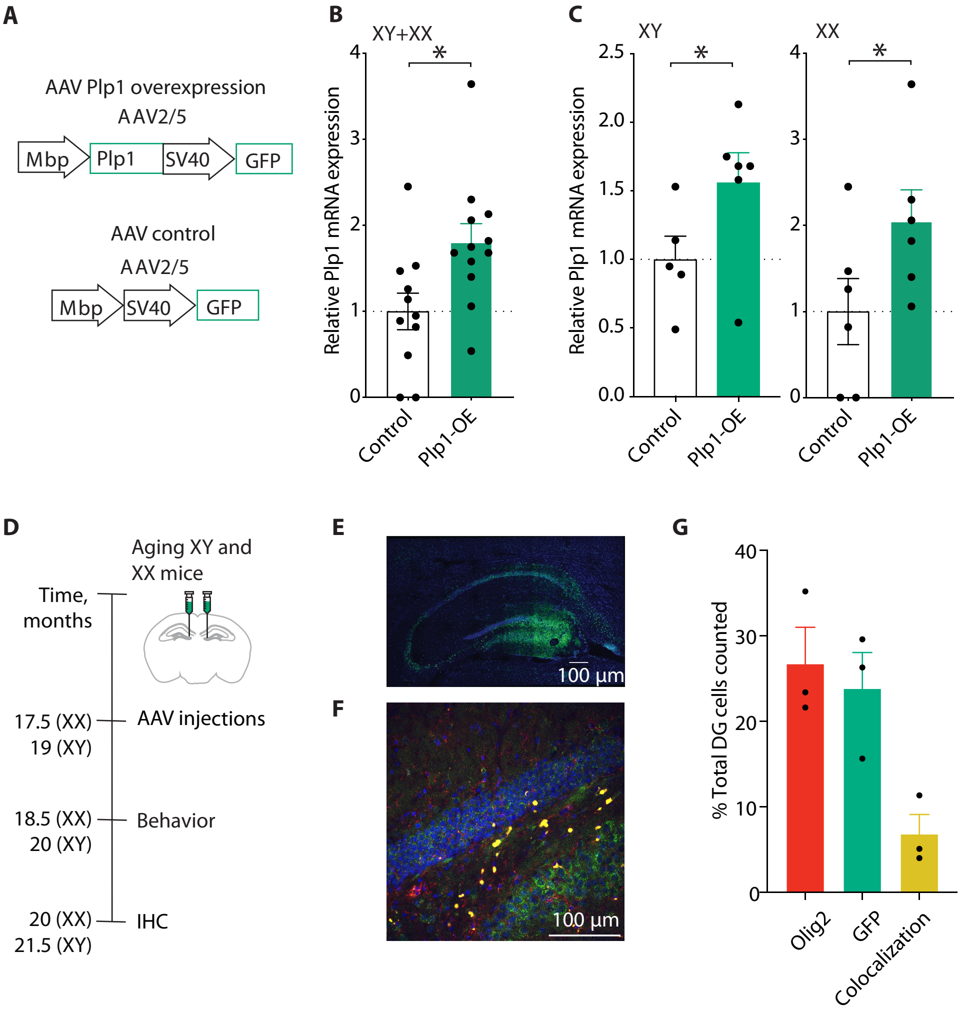

Fig. 6. AAV-mediated *Plp1* overexpression in vitro and in vivo. (**A**) Schematic of AAV2/5 viruses for *Plp1* overexpression (*Plp1*-OE) (top) and control (bottom). The *Plp1*-OE virus contains an oligodendrocyte-specific *Mbp* promoter driving *Plp1*, followed by an SV40 promoter transcribing GFP. The control virus is the same virus without *Plp1*. (**B**) Relative *Plp1* mRNA expression in XY and XX mixed glia infected with Control ($n = 11$ wells) and *Plp1*-OE ($n = 12$ wells) viruses (*$P = 0.0154$, two-tailed t test) representing two independent experiments separated by sex, shown in (C). (**C**) Relative *Plp1* mRNA expression in (left) XY mixed glia infected with Control ($n = 5$ wells) and *Plp1*-OE ($n = 6$ wells) viruses (*$P = 0.0409$, one-tailed t test) and (right) XX mixed glia mixed glia infected with Control ($n = 6$ wells) and *Plp1*-OE ($n = 6$ wells) viruses (*$P = 0.0386$ one-tailed t test). (**D**) Schematic of in vivo and ex vivo AAV hippocampal experiments in old XY and XX mice. Image credit: A. Moreno. (**E**) Representative 20× stitched image of the hippocampus with GFP infection largely in the DG region from the *Plp1*-OE virus (green) and DAPI (blue). Scale bar, 100 μm. (**F**) Representative 40× image of the DG with GFP infection from the *Plp1*-OE virus (green), OLIG2 oligodendrocyte marker (red), and DAPI (blue), indicating infected oligodendrocytes (merged, yellow). Scale bar, 100 μm. (**G**) Quantitation of the percentage of total cells counted in the DG expressing OLIG2 (oligodendrocyte marker) and GFP (marking AAV infection) and showing colocalization of both markers (yellow), indicating AAV-infected oligodendrocytes ($n = 3$ XX mice).

most DEGs in our pseudobulk analysis (Fig. 2, C and H), suggesting that it is particularly sensitive to aging. Using immunohistochemistry, we examined AAV infection of oligodendrocytes with immunofluorescence for GFP (Fig. 6, E and F) and OLIG2 (Fig. 6F), an oligodendrocyte marker. GFP (green) overlapped with OLIG2(red) in 6.8% of cells in the DG and 24.3% of oligodendrocytes within the region (Fig. 6G), confirming that the AAV infected oligodendrocytes.

Cell type–specific up-regulation of *Plp1* improves cognition in aging XY and XX mice

Finally, we tested whether increasing *Plp1*—through cell type–specific elevation in oligodendrocytes of the hippocampus—improved cognition in the aging XY and XX brain. To this end, we conducted a battery of behavioral tests in old male and female mice transfected with AAV2/5 with an empty vector (control) or *Plp1* overexpression (*Plp1*-OE) (Fig. 7A). *Plp1*-OE in oligodendrocytes did not alter anxiety-like behavior in the elevated plus maze (EPM) (Fig. 7B) or total activity in the open field (Fig. 7C) in either XY or XX mice. To probe spatial and working memory, domains preferentially targeted by aging (*117*), we then assessed mice in the two-trial large Y-maze. *Plp1*-OE in oligodendrocytes increased exploration in the novel compared to the familiar arm in both XY (Fig. 7D) and XX (Fig. 7E) mice, indicating that it improved cognition in aging mice of both sexes. Thus, *Plp1* elevation in the oligodendrocytes of the hippocampus—recapitulating a component of aging-induced activation of the silent Xi in females—enhanced learning and memory in aging brains of both XY and XX mice.

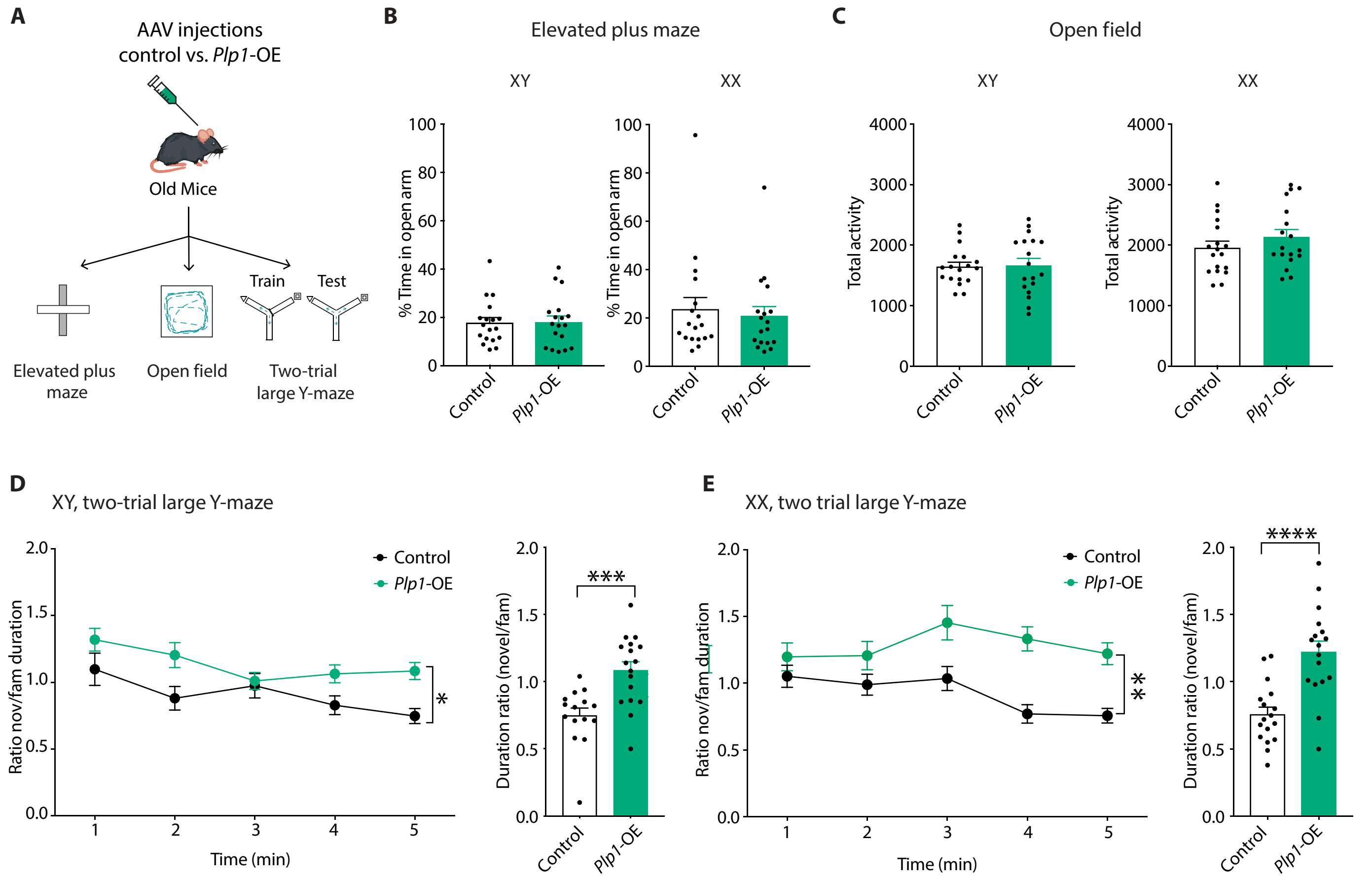

Fig. 7. Elevating *Plp1* in oligodendrocytes of the DG improves cognition in old XY and XX mice. (**A**) Experimental paradigm. Following AAV infection with AAV-control (Control) or AAV-*Plp1*-OE (*Plp1*-OE), old XX and XY mice underwent behavioral and cognitive tests including EPM, open field, and the two-trial large Y-maze. Image credit: A. Moreno and D. Velasco. (**B**) Percent time in the open arm of the EPM did not differ between old Control and *Plp1*-OE mice that were either XY (left) or XX (right) mice (age = 20 months XY; 18.5 months XX) ($n = 18$ mice per experimental group) ($P = 0.9071$ XY; $P = 0.6775$ XX, two-tailed unpaired t test). (**C**) Total activity in the open field did not differ between old Control and *Plp1*-OE mice that were either XY (left) or XX (right) (age = 20 months XY; 18.5 months) ($n = 18$ mice per experimental group) ($P = 0.8559$ XY; $P = 0.2802$ XX, two-tailed unpaired t test). (**D**) In old XY mice, *Plp1*-OE increased the duration in the novel compared to the familiar arm versus Controls in the two-trial large Y-maze across 5 min (left) (*$P = 0.0193$ treatment effect, two-way ANOVA) and averaged over 5 min (right) (***$P = 0.0005$, two-tailed unpaired t test) (age = 20.5 months) ($n = 15$ Control mice, $n = 17$ *Plp1*-OE mice). (**E**) In old XX mice, *Plp1*-OE increased the duration in the novel compared to the familiar arm versus Controls in the two-trial large Y-maze across 5 min (left) (**$P = 0.0020$ treatment effect, two-way ANOVA) and averaged over 5 min (right) (****$P < 0.0001$, two-tailed unpaired t test) (age = 19 months) ($n = 17$ Control mice, $n = 17$ *Plp1*-OE mice).

DISCUSSION

Our data show that aging remodels Xi, the silent X, across cell types of the female mouse hippocampus. Aging preferentially altered DEGs on the X compared to autosomes, and nearly globally up-regulated neuronal and glial DEGs from both Xa and Xi, in a cell type–specific manner. Focused analysis of Xi showed changes in escapee expression including new escape with aging, and collectively predicted transcriptional regulation of XCI and broad neural structures and functions including synaptic integrity, signaling, and constituents of myelin. *Plp1*, a component of myelin highly expressed in oligodendrocytes, was among the most common and robustly activated Xi genes with age, and also increased in the aging parahippocampus of women. AAV-mediated elevation of *Plp1* in oligodendrocytes of the DG, a key memory region targeted by aging, improved cognition in old mice of both sexes. Thus, recapitulation of a component of aging-induced activation of the silent X in females promoted better function of the old brain.

The study of female-specific biology is historically underrepresented in science and medicine but is essential and expanding fervently. This area of investigation is important because it (i) promotes an understanding of how fundamental biology contributes to health and disease in women and (ii) lays the groundwork to dissect sex differences between men and women. Unraveling the sex-based biology of what makes one sex more resilient or vulnerable paves the path to new and personalized treatments for both sexes. Our investigation of how aging affects the Xa and Xi chromosomes is specific to females and therefore forms a basis to understand sex differences, since typical males lack a second X.

The X, a major source of sex difference due to a second X in female mammals, represents 5% of the genome in men and women, and its study is growing rapidly, particularly in brain aging and age-related neurodegenerative conditions such as AD. Historically, analytic challenges posed by X hemizygosity in males, random XCI and baseline X escape in females, shared sequences between X and Y, and limited representation of the X in genome-wide association studies often resulted in X exclusion in studies (*118*, *119*). However, expanding tool kits and varied sequencing approaches offer innovative and

complimentary opportunities to study the X with high accuracy in the brain, as evidenced by several recent studies (*23*, *120*–*122*).

Our findings remarkably converge upon several targets identified in recent, unbiased, and large-scale human studies of the X in aging and AD. In these studies, either X expression (*23*) or its genetic variation (*120*–*122*) associated with genes coding for synaptic (*GRIA3*, *DMD*, *TENM1*, *FRMPD4*, *IL1RAPL1*, *GRIPAP1*), kinase or phosphatase (*PNCK*, *IRAK1*, *WNK3*, *MTM1*), ubiquitin ligase or deubiquitinase (*RLIM*, *OTUD5*), DNA polymerase or transcription (*AFF2*, *POLA1*), and sodium and potassium transporter (*SLC9A7*) factors—all of which were modulated in our study of aging-induced remodeling of Xa and Xi. Notably, genetic variation in *SLC9A7*, which underwent new escape with aging in oligodendrocytes (fig. S4D), associated with AD risk in a meta-analysis of over 1 million individuals (*122*). It will be particularly interesting to know how genetic variation alters cell type–specific SLC9A7 levels and function, and how that links to AD risk. This, along with the finding that around half of the aging-induced targets we identified on the Xi cause human intellectual disability if mutated (table S1), highlights the exceptional role of the X factors identified in contributing to cognition.

XCI was proposed in 1961 by Mary Lyon (*123*), followed by several decades of mechanistic advances (*24*, *124*–*130*). Newer tools such as single cell and snRNA-seq now enable more nuanced study of XCI escape across cell types (*131*–*134*), and allele-specific approaches enable direct measure of Xi expression (*25*, *27*–*29*, *135*–*139*). Our study uniquely combines snRNA-seq with an allele-specific approach to understand how aging alters Xa and Xi expression across cell types of the hippocampus, a region critical to cognition and targeted by aging, AD, and other neurodegenerative diseases.

Aging-induced activation of Xi, either through increased escapee expression or new escape altogether, increases the dose of activated X genes in cell types of the XX hippocampus. In other words, the aging female brain carries higher X expression due to the second "inactive" X. It is interesting to speculate that the X increase may benefit the aging brain since it is enriched for cognition-related genes. This may, in part, underlie why adding a second X to XY male mice improves, while subtracting one from XX female mice worsens, hippocampal-dependent cognitive functions in aging and an AD model (*22*, *23*). It may also link with female-based resilience to cognitive aging (*16*–*20*) and higher baseline memory functions in typical aging and early AD (*16*, *20*, *21*, *140*) observed in women, across many populations. However, X genes also harbor immune-related functions that exacerbate phenotypes, as noted with *Tlr7* in the brains of aging male mice (*141*) and *Kdm6a* in lymphocytes of female mice in an experimental model of autoimmune encephalitis (*142*). In addition, an X factor, USP11, promotes tau modification and pathology in female mice (*143*). Whether these factors escaped from the Xi is unclear in these studies, but it is important to note that while an increase in Xi gene expression could confer cognitive resilience at large, not all X factors will uniformly benefit the brain at all life stages and conditions. Each will require causal testing for their cell type–specific role(s) in brain aging and neurodegenerative diseases. What Xi activation broadly means for women's brain health—or for other systems of the body—is now a critical area of investigation.

Here, increasing *Plp1* expression in oligodendrocytes of the DG, which recapitulates a component of age-induced Xi activation, improved cognition in old XX and XY mice. First, *Plp1* is robustly expressed in oligodendrocytes, where it promotes myelination, a principal regulator of axonal conductance, and target of age-induced degeneration (*144*–*147*) that links to cognition (*148*). Oligodendrocyte vulnerability in aging impairs myelin plasticity through decreased remyelination and regenerative capacities alongside attenuated metabolic shuttling of lactate and pyruvate to axons (*43*). Therefore, increasing *Plp1* in this cell type may boost the functions of oligodendrocytes and counter age-induced vulnerabilities.

Increasing *Plp1* in the small hippocampal subregion of the DG improved cognitive functions, consistent with findings that interventions in cells forming a functional hub can modulate larger networks (*149*). Furthermore, *Plp1*-mediated cognitive improvement was acute and occurred when the intervention was introduced during old age, not requiring lifetime exposure to a transgenic alteration. This is important because treatments for cognitive aging, and for neurodegenerative diseases like AD, will require treatment late in life, and perhaps after symptoms emerge.

Beyond *Plp1*, aging activated the escape of several genes from the "inactive" Xi, suggesting its heightened accessibility to transcription. Epigenetic alterations are a hallmark of aging (*150*), and XCI is maintained by epigenetic programs of methylation, heterochromatin, and noncoding RNAs. It follows that the process of aging itself alters these programs across the genome including on the X (*151*), causing an overall loss of repression and increased transcription (*150*, *152*, *153*). More specifically, aging increases variability of the methylation (*151*) that widely silences genes on Xi (*154*), as observed in demethylation of regions escaping XCI in human leukocytes (*153*). Furthermore, aging increases chromatin accessibility (*152*), promoting increased gene expression (*155*), across the genome and particularly on topological-associated domain-like structures with high chromatin accessibility where escapees tend to cluster (*156*), as observed in hematopoietic stem cells (*152*). Collectively, these examples provide mechanistic insight into how aging may activate Xi in the brain, possibilities that require further study in neural cell types.

Given the density and high volume of epigenetic repression involved in maintaining XCI, the silent X may unintentionally but preferentially be affected by the global alterations of aging. Alternatively, or additionally, the aging-driven activation of Xi may offer an evolutionary advantage to females by increasing X dose, and thus potentially conferring resilience to cognitive decline. In the context of the debated "grandmother hypothesis" (*157*–*161*), a sharp mind and knowledge of resources could enhance the survival, reproductive success, and genetic fitness of a female's children, grandchildren, and local community. Whether or not activation of the Xi is an intended or unintended consequence of aging, this remarkable process of partial "awakening" with age, first noted in the in 1980s in the liver (*162*), requires further investigation. The timing of Xi activation, its mechanistic and epigenetic orchestration, its conservation and overlap with humans (who show more X escape at baseline than mice) (*163*–*165*), and its functional significance for the aging female brain are all high-value areas of future research.

Emulating the effects of escape from XCI or even directly activating Xi genes could be a potential therapeutic strategy in brain aging and neurodegenerative diseases. Treatments simulating escape from XCI have been explored to treat Rett syndrome and other X-linked conditions. Since Rett syndrome is caused by a mutation in *MEPC2* on one X (*166*, *167*), therapies to reactivate the silenced wild-type *MEPC2* or replace the mutated gene have been proposed (*166*, *168*, *169*). Our studies suggest that targeted elevation of *Plp1*, which is down-regulated

in some studies of AD (*170*)—or perhaps targeting a number of other activated Xi genes—might improve cognitive deficits in aging and neurodegenerative disease.

In our analysis, most DEGs (Fig. 2E) and escapees (fig. S4) were cell type specific (60% and 56%, respectively), yielding high resolution of the Xi with aging. Notably, cell type classification was recapitulated by manual and automated (*171*, *172*) annotation with 96% cell class overlap (data S7). While snRNA-seq datasets are inherently confounded by doublets and ambient RNA to some degree (*173*), we largely accounted for these confounders using Scrublet (*174*) and Cell Ranger, balancing resolution of cell type specificity while maintaining sufficient power to detect allele-specific reads.

Our study has several caveats and limitations. First, our experiments did not include males, who harbor a single Xa. Whether aging similarly increases X expression in males, as it does in females, remains an important question. Second, we did not study how parent-of-X origin, known to influence X gene expression in neurons (*175*), influences the age-induced modulation of the Xa or Xi in females. Third, in our allele-specific analysis, which uses two different reference genomes, mapping bias toward *M. musculus* may have occurred since it is a more refined and established genome than that of *M. castaneus*, which is more newly generated (*176*, *177*). This could result in capturing less of the Xi and lead to a more conservative analysis. Fourth, while a great many SNPs differentiate the two mouse genomes, increasing discrimination of the Xa from Xi, some genomic areas show scant SNPs, which could lead to decreased discrimination in some genes. However, this genetic structure, consistent between young and old mice, would not influence age-induced escape findings. Fifth, genetic deletion of *Xist* on the Xa chromosome, used to enforce nonrandom XCI, may have precluded our detection of age-induced *Xist* changes. Finally, like for any snRNA-seq library, selection bias of RNAs captured in our study was unavoidable, as was the twofold decrease in power resulting from measuring each gene twice to assess allele-specific expression.

In summary, our study reveals aging-induced activation, repression, and remodeling of the "silent" Xi and its cell type specificity, with high resolution. Since Xi activation increases X dose, and X genes disproportionately influence neural and cognitive functions, our findings may imply a mechanism for female-based resilience to cognitive aging and increased baseline functions in early AD (*16*–*21*). Understanding how Xi escapees, such as *Plp1*, can contribute to counteracting vulnerabilities of brain aging may pave the way to therapeutic targets that benefit one or both sexes.

MATERIALS AND METHODS

Animals

All mouse studies were approved by the Institutional Animal Care and Use Committee (IACUC) of the University of California, San Francisco (IACUC protocol number: AN204838). The studies complied with the National Institutes of Health guidelines. Mice were on a 12-hour light/dark cycle, in standard housing groups of five mice per cage with ad libitum access to food and water.

snRNA-seq mice

Female *M. musculus Xist*$^{\text{loxP+/−,Zp3-cre}}$ mice (*30*, *178*) with a congenic C57BL/6J background were crossed with male *M. castaneus* mice obtained from The Jackson Laboratory (strain 000928). XX progeny carrying the *Xist*$^{\text{loxP+/−,Zp3-cre}}$ allele were used for snRNA-seq. Each experimental group consisted of age-matched, littermate controls. Four young (3 months) and four old (22 months) mice were euthanized. Their fresh hippocampi were dissected and frozen for downstream studies.

Plp1 overexpression in mice

Aged male (19 months) and female (17.5 months) mice from a congenic C57BL/6J background were used for *Plp1* overexpression studies. *Plp1* was up-regulated using an AAV2/5 vector that encodes *Plp1* [National Center for Biotechnology Information (NCBI) Reference Sequence: NM_011123; 894 bp] from Applied Biological Materials (catalog number: #370271040110). The cytomegalovirus promoter was changed to an *Mbp* promoter. Downstream of *Plp1*, *Gfp* with an SV40 promoter is present. An additional control virus was used with the same sequence as the overexpression virus but no *Plp1*.

Mice were anesthetized (2 to 3% isoflurane) and placed in a stereotaxic frame using ear bars and tooth bar. AAV (5 μl per hemisphere) was stereotaxically injected into each DG of the hippocampus using the coordinates anterior-posterior (AP) = −2.1, medial-lateral (ML) = ±1.7, and dorsal-ventral (DV) = 1.9 at 3 μl/min, allowing for 10 min of diffusion. Experimenters were blinded to the identity of the viral injection.

Sequencing and bioinformatics

snRNA preparation

Nuclei were extracted using the Nuclei EZ Prep kit (Millipore Sigma), following the manufacturer's protocol. Frozen hippocampi were homogenized in lysis buffer and centrifuged twice to isolate the nuclei from the cell debris. The nuclei were resuspended in glycerol-containing buffer and stored at −80°C. The libraries were prepped with the goal of 5000 captured nuclei per sample using the Chromium Single Cell 3′ Library and Gel Bead Kit v3.1, Chromium Next GEM Chip G, and Dual Index Kit TT Set A (10x Genomics). The samples were sequenced by Novogene USA on an Illumina NovaSeq 6000 with paired-end 150-bp reads PE150 (Novogene) with the goal of 100,000 reads per target captured cell.

snRNA-seq data analysis

A combined *M. musculus* (GRCm39) and *M. castaneus* (CAST_EiJ_v1.111) genome was created using the mkref function of Cell Ranger (Cell Ranger/8.0 10x Genomics). FASTQ files were aligned to the combined reference genome using Cell Ranger Count v8.0.0 with the -include introns flag. Combined feature-barcode matrixes were formed using the aggregate function of Cell Ranger (Aggr v8.0.0). The resultant feature-barcode matrix was analyzed using Scanpy (*31*). The matrix was filtered by removing barcodes with less than 100 corresponding genes and removing genes associated with fewer than three barcodes.

Scrublet was used to remove predicted doublets from the dataset (*174*). Data were normalized to total counts and logarithmized. Dimension reduction analysis was conducted using uniform manifold approximation and projection (UMAP). The Leiden algorithm was used to cluster the cells at a 0.5 resolution. The Wilcoxian method was used to determine the top 100 DEGs in each group. The genes were compared to clusters in the Allen Brain Atlas 10x hippocampus and cortex dataset, and clusters were assigned (*32*). Clusters with similar markers were combined for downstream analysis. Automated annotation was conducted with CellTypist (*171*, *172*).

For DEG analysis, barcodes were pseudobulked by sample and cell cluster for downstream analysis using the decoupler and pyDEseq2 programs (*36*, *37*) after filtering out genes with a minimum of 10 reads across samples and 10 counts in an individual sample. Genes were considered to be differentially expressed if the adjusted *P* value was

<0.05, and the $\log_2$ fold change was >0.1 or <−0.1. Typical to DEseq2 pipelines, the *P* values are calculated with the Wald test and are adjusted using the Benjamini-Hochberg procedure.

GO term analysis was conducted for each cell type. Genes were ranked using the Scanpy function "rank_genes_groups" and grouped by cell type. The top 350 X chromosome genes (ranked by *P* value and $\log_2$ fold change) were evaluated with the g:Profiler if their adjusted *P* value was <0.05, and the $\log_2$ fold change was >0.1 or <−0.1 (*39*). *P* values are adjusted with the g:SCS method (*179*). GO term analysis was also conducted on significant (adjusted *P* value was <0.05, and the $\log_2$ fold change was >0.1 or <−0.1) X DEGs using g:Profiler (*39*).

Computational escape

Escape was assessed using a method adapted from Berletch *et al.* (*25*). The *M. musculus* and *M. castaneus* normalized reads were summed for each gene, and the escape ratio was evaluated by taking the proportion of *M. castaneus* reads from the total reads for the gene (Eq. 1).

Xa is the total normalized reads assigned to the *M. musculus* genome for the given cell type and age context.

Xi is the total normalized reads assigned to the *M. castaneus* genome for the given cell type and age context.

P_{esc} is the ratio of X_i reads

$$P_{esc} = \frac{Xi}{Xi + Xa} \tag{1}$$

The escape ratio was corrected for mapping bias using the methods explained by Berletch *et al.* (*25*) using the ratio of the total autosomal reads to the *M. musculus* and *M. castaneus* genomes (Eq. 2).

A_{Tcast} is the total normalized autosomal reads from the *M. castaneus* genome across all genes.

A_{Tmus} is the total normalized autosomal reads from the *M. musculus* genome across all genes.

P_{escadj} is the adjusted escape ratio

$$P_{escadj} = \frac{P_{esc}}{\left[P_{esc} + \left(\frac{A_{Tcast}}{A_{Tmus}}\right)(1 - P_{esc})\right]} \tag{2}$$

A 99% CI was conducted for each escape ratio (Eq. 3)

$$99\%CI = P_{escadj} \pm 2.575\sqrt{\frac{P_{escadj}(1 - P_{escadj})}{Xi + Xa}} \tag{3}$$

If the lower bound of the CI was greater than 0 and the corrected escape value was greater than 0.05, the gene was considered to escape. If Xa or Xi was lower than the 5th percentile for X allelic reads in all cell types, the gene was not considered an escapee. If Xa was lower than the 5th percentile for X allelic reads in all cell types, age-related escape trends were not calculated. Escape was assessed by cell type and age group. GO term analysis was conducted on escapees following certain trends with aging using the program g:Profiler (*39*).

Human PLP1 analysis

MSBB cohort data were accessed from the Accelerating Medicines Partnership Alzheimer's Disease (AMP-AD) knowledge portal at Synapse (*180*). Four different cortical regions—Broadmann area 10 (frontal pole), Broadmann area 22 (superior temporal gyrus), Broadmann area 36 (parahippocampal gyrus), and Broadmann area 44 (inferior temporal gyrus)—were analyzed (*181*). Using the DESeq2 package in R, raw *PLP1* count data for each region were normalized for library size and modeled as a function of sex while adjusting for standard covariates used in the MSBB processing pipeline: batch, age at death, race, postmortem interval, RIN, exonic rate, and rRNA rate (*180*, *182*). Sex differences are reported as $\log_2$ fold change of normalized *PLP1* expression. Cases with available uncensored age-at-death data were included in the final analysis (frontal pole: $n = 161$, superior temporal gyrus: $n = 159$, parahippocampal gyrus: $n = 184$, inferior temporal gyrus: $n = 150$).

In vitro and in vivo experiments

Immunohistochemistry

AAV-infected mice (2.5 months postinfection) were perfused with a peristaltic pump for 4 min using cold phosphate-buffered saline (PBS) (10 ml/min). Whole brains were collected and postfixed for 48 hours in 4% (w/v) paraformaldehyde. The brains were preserved in 30% (w/v) sucrose in PBS. A freezing sliding microtome (Leica) was used to section whole brains into 40-μm-thick coronal slices and stored in cryoprotective medium at −20°C.

Individual sections were blocked with 10% normal donkey serum. The blocked sections were incubated with primary antibodies [rabbit anti-GFP (1:1000, Sigma-Aldrich, G1544) and mouse anti-OLIG2 (1:200, Proteintech, 66513-1-IG)] at 4°C overnight. The sections were washed and incubated with secondary antibodies [donkey anti-rabbit 488, donkey anti-mouse 594 Alexa Fluor–conjugated secondary antibodies (1:1000, Invitrogen A-21206, Invitrogen R37115)] at room temperature for 2 hours, with a final addition of 300 nM 4′,6-diamidino-2-phenylindole (DAPI). Sections were washed and mounted on a microscope (Nikon CSU-W1) using Micro Manger 2.0 Gamma (*183*). The microscope has a Zyla 4.2 CMOS camera (Andor), piezo XYZ stage (ASI), CSU-W1 Spinning Disk with Borealis upgrade (Yokogowa/Andor), Spectra-X (Lumencor), CSU-W1 Penta Dichroic 405/488/561/640/755, ILE 4 line solid-state Laser Launch a slide with Vectashield.

Sections were imaged on the fluorescence microscope with a spinning disk confocal (405/488/561/640 nm; Andor). Images were taken using the Plan Apo λ 20×/0.75 objective using solid-state lasers 405 and 488 nm and emission filters 447/60 and 525/50 (Semrock) (for DAPI and GFP, respectively). Additional images were taken with the Plan Apo λ 40×/1.3 oil objective using solid-state lasers 405, 488, and 561 nm and emission filters 447/60, 525/50, and 607/36 (Semrock) [for DAPI, GFP, and red fluorescent protein (RFP), respectively]. Images were stitched and processed using Fiji (*184*). Images (40×) were used for cell counting, described below.

Cell counting

Microscopy images were analyzed using the segment artificial intelligence (AI) analysis from the Nikon NIS Elements AR software version 5.41.00 64bit. The images were preprocessed using the Mexican Hat, Fast Smoothing, and Subtracting backgrounds functions to make the nuclei clearer and increase the ability of the software to identify the nuclei. The segment AI was trained on 512 × 512 segments of the DG until the AI was reliably able to identify about 95% of DG nuclei. The trained segment AI was then applied to the DG images, where it counted the nuclei and measured the intensity of GFP and RFP in the cytoplasm. A threshold of 350 intensity units and 200 intensity units was set for RFP and GFP, respectively. All nuclei that met that threshold were recorded as positive for that signal. Cells that were positive for GFP and RFP were counted as cells with colocalization.

Mixed glial culture

Using methods previously described (*185*), primary mixed glia were cultured from postnatal 0 to 2 congenic C57BL/6J male XY and female XX mouse pups. Cells were plated at a density of 10^6 cells/ml

in 24-well plates. The cells matured in Dulbecco's modified Eagle's medium. At day 5 (division 2) in vitro, they were treated with the *Plp1* AAV (described in the *Plp1*-OE mouse section) at multiplicity of infection (MOI) = 2. At division 4, cells were treated with cytosine arabinoside to reduce glial proliferation (*186*). At division 10, cells were harvested, and RNA was isolated.

Quantitative PCR

qPCR was conducted on whole hippocampi from young (3 months) (n = 13; XX, n = 15) and old (20 to 35 months) (XY, n = 10; XX, n = 15) XX and XY mice. Mouse *Plp1* transcript variant 2 F: 5′CTTCCTTTATGGGGCCCTCC and R: 5′GGTGGTCTTGTAGTCGCCAA primers were used.

Behavior

Behavioral studies were conducted beginning 4 weeks after AAV injection, and experimenters were blinded to the treatments. Studies were conducted on age-matched controls and during the light cycle. All chambers, objects, and areas were cleaned with 70% alcohol between testing sessions.

Elevated plus maze

The EPM was carried out as described (*187*, *188*). For 1 hour before testing, mice were habituated in the testing room in dim light. During testing, mice were placed in the center of the EPM facing the open arm and could explore for 10 min. Their time spent in each region and distance traveled in the open and closed arms were recorded using the Kinder Scientific Elevated Plus-Maze (Chula Vista, CA) and MotorMonitor system.

Open field

The open field was carried out as described (*23*, *187*–*189*). Mice were acclimated for 30 min and explored the open field for 10 min. The total activity in the open field (clear chamber 41 × 30 cm) was detected by beam breaks and measured with an automated Flex-Field/Open Field Photobeam Activity System (San Diego Instruments).

Two-trial large Y-maze

The mice were evaluated in the two-trial large Y-maze following the described protocols (*188*, *190*, *191*). Mice were trained by allowing exploration of one arm of the Y with a visual cue. Following 16 hours after completion of training, the novel arm with a different visual cue was opened and mice were allowed access. Mice then explored all arms of the two-trial large Y-maze. The duration in the novel compared to the familiar arm, a measure of spatial learning and memory, was assessed.

Statistical analysis

Statistical analyses for in vitro and in vivo behavioral experiments were carried out using GraphPad Prism (version 7.0) for t tests and two-way analyses of variance (ANOVAs). All tests were two-tailed unless indicated otherwise. Differences between two means were assessed using unpaired t tests and a two-way ANOVA to assess differences among multiple means for all experiments unless otherwise stated. Error bars represent SEM, and null hypotheses were rejected at or below a P value of 0.05 when rounded to two decimal points.

Supplementary Materials

The PDF file includes:
Supplementary Text
Figs. S1 to S5
Table S1
Legends for data S1 to S8
References

Other Supplementary Material for this manuscript includes the following:
Data S1 to S8

REFERENCES AND NOTES

1. D. B. Dubal, C. Murphy, Y. Suh, B. A. Benayoun, Biological sex matters in brain aging. *Neuron* **113**, 2–6 (2025).
2. V. Zarulli, J. A. Barthold Jones, A. Oksuzyan, R. Lindahl-Jacobsen, K. Christensen, J. W. Vaupel, Women live longer than men even during severe famines and epidemics. *Proc. Natl. Acad. Sci. U.S.A.* **115**, E832–E840 (2018).
3. United Nations Department of Economic and Social Affairs, Population Division, *World Population Ageing* (United Nations Department of Economic and Social Affairs, 2020).
4. A. M. Bronikowski, R. P. Meisel, P. R. Biga, J. R. Walters, J. E. Mank, E. Larschan, G. S. Wilkinson, N. Valenzuela, A. M. Conard, J. P. de Magalhaes, J. E. Duan, A. E. Elias, T. Gamble, R. M. Graze, K. E. Gribble, J. A. Kreiling, N. C. Riddle, Sex-specific aging in animals: Perspective and future directions. *Aging Cell* **21**, e13542 (2022).
5. E. L. Barrett, D. S. Richardson, Sex differences in telomeres and lifespan. *Aging Cell* **10**, 913–921 (2011).
6. A. M. Bronikowski, J. Altmann, D. K. Brockman, M. Cords, L. M. Fedigan, A. Pusey, T. Stoinski, W. F. Morris, K. B. Strier, S. C. Alberts, Aging in the natural world: Comparative data reveal similar mortality patterns across primates. *Science* **331**, 1325–1328 (2011).
7. T. H. Clutton-Brock, K. Isvaran, Sex differences in ageing in natural populations of vertebrates. *Proc. Biol. Sci.* **274**, 3097–3104 (2007).
8. M. Bou Sleiman, S. Roy, A. W. Gao, M. C. Sadler, G. V. G. von Alvensleben, H. Li, S. Sen, D. E. Harrison, J. F. Nelson, R. Strong, R. A. Miller, Z. Kutalik, R. W. Williams, J. Auwerx, Sex- and age-dependent genetics of longevity in a heterogeneous mouse population. *Science* **377**, eabo3191 (2022).
9. C. J. Cheng, J. A. L. Gelfond, R. Strong, J. F. Nelson, Genetically heterogeneous mice exhibit a female survival advantage that is age- and site-specific: Results from a large multi-site study. *Aging Cell* **18**, e12905 (2019).
10. E. J. Davis, I. Lobach, D. B. Dubal, Female XX sex chromosomes increase survival and extend lifespan in aging mice. *Aging Cell* **18**, e12871 (2019).
11. S. N. Austad, K. E. Fischer, Sex differences in lifespan. *Cell Metab.* **23**, 1022–1033 (2016).
12. E. M. Crimmins, J. K. Kim, A. Sole-Auro, Gender differences in health: Results from SHARE, ELSA and HRS. *Eur. J. Public Health* **21**, 81–91 (2011).
13. E. H. Gordon, R. E. Hubbard, Do sex differences in chronic disease underpin the sex-frailty paradox? *Mech. Ageing Dev.* **179**, 44–50 (2019).
14. S. Horvath, M. Gurven, M. E. Levine, B. C. Trumble, H. Kaplan, H. Allayee, B. R. Ritz, B. Chen, A. T. Lu, T. M. Rickabaugh, B. D. Jamieson, D. Sun, S. Li, W. Chen, L. Quintana-Murci, M. Fagny, M. S. Kobor, P. S. Tsao, A. P. Reiner, K. L. Edlefsen, D. Absher, T. L. Assimes, An epigenetic clock analysis of race/ethnicity, sex, and coronary heart disease. *Genome Biol.* **17**, 171 (2016).
15. M. S. Goyal, T. M. Blazey, Y. Su, L. E. Couture, T. J. Durbin, R. J. Bateman, T. L. Benzinger, J. C. Morris, M. E. Raichle, A. G. Vlassenko, Persistent metabolic youth in the aging female brain. *Proc. Natl. Acad. Sci. U.S.A.* **116**, 3251–3255 (2019).
16. E. M. Arenaza-Urquijo, R. Boyle, K. Casaletto, K. J. Anstey, C. Vila-Castelar, A. Colverson, E. Palpatzis, J. M. Eissman, T. K. S. Ng, S. Raghavan, M. Akinci, J. M. J. Vonk, L. S. Machado, P. P. Zanwar, H. L. Shrestha, M. Wagner, S. Tamburin, H. R. Sohrabi, S. Loi, D. Bartres-Faz, D. B. Dubal, P. Vemuri, O. Okonkwo, T. J. Hohman, M. Ewers, R. F. Buckley, Reserve, Resilience and Protective Factors Professional Interest Area, Sex and Gender Professional Interest area and the ADDRESS! Special Interest Group, Sex and gender differences in cognitive resilience to aging and Alzheimer's disease. *Alzheimers Dement.* **20**, 5695–5719 (2024).
17. K. B. Casaletto, F. M. Elahi, A. M. Staffaroni, S. Walters, W. R. Contreras, A. Wolf, D. Dubal, B. Miller, K. Yaffe, J. H. Kramer, Cognitive aging is not created equally: Differentiating unique cognitive phenotypes in "normal" adults. *Neurobiol. Aging* **77**, 13–19 (2019).
18. C. R. Jack Jr., H. J. Wiste, S. D. Weigand, D. S. Knopman, P. Vemuri, M. M. Mielke, V. Lowe, M. L. Senjem, J. L. Gunter, M. M. Machulda, B. E. Gregg, V. S. Pankratz, W. A. Rocca, R. C. Petersen, Age, sex, and APOE ε4 effects on memory, brain structure, and β-amyloid across the adult life span. *JAMA Neurol.* **72**, 511–519 (2015).
19. D. B. Dubal, Sex difference in Alzheimer's disease: An updated, balanced and emerging perspective on differing vulnerabilities. *Handb. Clin. Neurol.* **175**, 261–273 (2020).
20. A. C. McCarrey, Y. An, M. H. Kitner-Triolo, L. Ferrucci, S. M. Resnick, Sex differences in cognitive trajectories in clinically normal older adults. *Psychol. Aging* **31**, 166–175 (2016).
21. D. A. Levine, A. L. Gross, E. M. Briceno, N. Tilton, B. J. Giordani, J. B. Sussman, R. A. Hayward, J. F. Burke, S. Hingtgen, M. S. V. Elkind, J. J. Manly, R. F. Gottesman, D. J. Gaskin, S. Sidney, R. L. Sacco, S. E. Tom, C. B. Wright, K. Yaffe, A. T. Galecki, Sex differences in cognitive decline among US adults. *JAMA Netw. Open* **4**, e210169 (2021).
22. F. Marino, D. Wang, G. E. Merrihew, M. J. MacCoss, D. B. Dubal, A second X chromosome improves cognition in aging male and female mice. bioRxiv 2024.07.26.605328 [Preprint] (2024). https://doi.org/10.1101/2024.07.26.605328.
23. E. J. Davis, L. Broestl, S. Abdulai-Saiku, K. Worden, L. W. Bonham, E. Minones-Moyano, A. J. Moreno, D. Wang, K. Chang, G. Williams, B. I. Garay, I. Lobach, N. Devidze, D. Kim, C. Anderson-Bergman, G. Q. Yu, C. C. White, J. A. Harris, B. L. Miller, D. A. Bennett, A. P. Arnold, P. L. De Jager, J. J. Palop, B. Panning, J. S. Yokoyama, L. Mucke, D. B. Dubal, A

second X chromosome contributes to resilience in a mouse model of Alzheimer's disease. *Sci. Transl. Med.* **12**, eaaz5677 (2020).
24. A. Loda, S. Collombet, E. Heard, Gene regulation in time and space during X-chromosome inactivation. *Nat. Rev. Mol. Cell Biol.* **23**, 231–249 (2022).
25. J. B. Berletch, W. Ma, F. Yang, J. Shendure, W. S. Noble, C. M. Disteche, X. Deng, Escape from X inactivation varies in mouse tissues. *PLOS Genet.* **11**, e1005079 (2015).
26. L. Carrel, H. F. Willard, Heterogeneous gene expression from the inactive X chromosome: An X-linked gene that escapes X inactivation in some human cell lines but is inactivated in others. *Proc. Natl. Acad. Sci. U.S.A.* **96**, 7364–7369 (1999).
27. F. Yang, T. Babak, J. Shendure, C. M. Disteche, Global survey of escape from X inactivation by RNA-sequencing in mouse. *Genome Res.* **20**, 614–622 (2010).
28. H. Wu, J. Luo, H. Yu, A. Rattner, A. Mo, Y. Wang, P. M. Smallwood, B. Erlanger, S. J. Wheelan, J. Nathans, Cellular resolution maps of X chromosome inactivation: Implications for neural development, function, and disease. *Neuron* **81**, 103–119 (2014).
29. J. B. Berletch, W. Ma, F. Yang, J. Shendure, W. S. Noble, C. M. Disteche, X. Deng, Identification of genes escaping X inactivation by allelic expression analysis in a novel hybrid mouse model. *Data Brief* **5**, 761–769 (2015).
30. G. Csankovszki, B. Panning, B. Bates, J. R. Pehrson, R. Jaenisch, Conditional deletion of *Xist* disrupts histone macroH2A localization but not maintenance of X inactivation. *Nat. Genet.* **22**, 323–324 (1999).
31. F. A. Wolf, P. Angerer, F. J. Theis, SCANPY: Large-scale single-cell gene expression data analysis. *Genome Biol.* **19**, 15 (2018).
32. Z. Yao, C. T. J. van Velthoven, T. N. Nguyen, J. Goldy, A. E. Sedeno-Cortes, F. Baftizadeh, D. Bertagnolli, T. Casper, M. Chiang, K. Crichton, S. L. Ding, O. Fong, E. Garren, A. Glandon, N. W. Gouwens, J. Gray, L. T. Graybuck, M. J. Hawrylycz, D. Hirschstein, M. Kroll, K. Lathia, C. Lee, B. Levi, D. McMillen, S. Mok, T. Pham, Q. Ren, C. Rimorin, N. Shapovalova, J. Sulc, S. M. Sunkin, M. Tieu, A. Torkelson, H. Tung, K. Ward, N. Dee, K. A. Smith, B. Tasic, H. Zeng, A taxonomy of transcriptomic cell types across the isocortex and hippocampal formation. *Cell* **184**, 3222–3241.e26 (2021).
33. K. H. Hajdarovic, D. Yu, L. A. Hassell, S. Evans, S. Packer, N. Neretti, A. E. Webb, Single-cell analysis of the aging female mouse hypothalamus. *Nat. Aging* **2**, 662–678 (2022).
34. A. Zeisel, A. B. Munoz-Manchado, S. Codeluppi, P. Lonnerberg, G. La Manno, A. Jureus, S. Marques, H. Munguba, L. He, C. Betsholtz, C. Rolny, G. Castelo-Branco, J. Hjerling-Leffler, S. Linnarsson, Brain structure. Cell types in the mouse cortex and hippocampus revealed by single-cell RNA-seq. *Science* **347**, 1138–1142 (2015).
35. B. Tasic, Z. Yao, L. T. Graybuck, K. A. Smith, T. N. Nguyen, D. Bertagnolli, J. Goldy, E. Garren, M. N. Economo, S. Viswanathan, O. Penn, T. Bakken, V. Menon, J. Miller, O. Fong, K. E. Hirokawa, K. Lathia, C. Rimorin, M. Tieu, R. Larsen, T. Casper, E. Barkan, M. Kroll, S. Parry, N. V. Shapovalova, D. Hirschstein, J. Pendergraft, H. A. Sullivan, T. K. Kim, A. Szafer, N. Dee, P. Groblewski, I. Wickersham, A. Cetin, J. A. Harris, B. P. Levi, S. M. Sunkin, L. Madisen, T. L. Daigle, L. Looger, A. Bernard, J. Phillips, E. Lein, M. Hawrylycz, K. Svoboda, A. R. Jones, C. Koch, H. Zeng, Shared and distinct transcriptomic cell types across neocortical areas. *Nature* **563**, 72–78 (2018).
36. B. Muzellec, M. Telenczuk, V. Cabeli, M. Andreux, PyDESeq2: A python package for bulk RNA-seq differential expression analysis. *Bioinformatics* **39**, btad547 (2023).
37. I. M. P. Badia, J. Velez Santiago, J. Braunger, C. Geiss, D. Dimitrov, S. Muller-Dott, P. Taus, A. Dugourd, C. H. Holland, R. O. Ramirez Flores, J. Saez-Rodriguez, decoupleR: Ensemble of computational methods to infer biological activities from omics data. *Bioinform. Adv.* **2**, vbac016 (2022).
38. M. Ximerakis, S. L. Lipnick, B. T. Innes, S. K. Simmons, X. Adiconis, D. Dionne, B. A. Mayweather, L. Nguyen, Z. Niziolek, C. Ozek, V. L. Butty, R. Isserlin, S. M. Buchanan, S. S. Levine, A. Regev, G. D. Bader, J. Z. Levin, L. L. Rubin, Single-cell transcriptomic profiling of the aging mouse brain. *Nat. Neurosci.* **22**, 1696–1708 (2019).
39. L. Kolberg, U. Raudvere, I. Kuzmin, P. Adler, J. Vilo, H. Peterson, g:Profiler-interoperable web service for functional enrichment analysis and gene identifier mapping (2023 update). *Nucleic Acids Res.* **51**, W207–W212 (2023).
40. U. Zechner, M. Wilda, H. Kehrer-Sawatzki, W. Vogel, R. Fundele, H. Hameister, A high density of X-linked genes for general cognitive ability: A run-away process shaping human evolution? *Trends Genet.* **17**, 697–701 (2001).
41. N. K. Hussain, G. M. Thomas, J. Luo, R. L. Huganir, Regulation of AMPA receptor subunit GluA1 surface expression by PAK3 phosphorylation. *Proc. Natl. Acad. Sci. U.S.A.* **112**, E5883–E5890 (2015).
42. W. Ba, J. van der Raadt, N. N. Kasri, Rho GTPase signaling at the synapse: Implications for intellectual disability. *Exp. Cell Res.* **319**, 2368–2374 (2013).
43. T. W. Chapman, R. A. Hill, Myelin plasticity in adulthood and aging. *Neurosci. Lett.* **715**, 134645 (2020).
44. E. Bagdatlioglu, P. Porcari, E. Greally, A. M. Blamire, V. W. Straub, Cognitive impairment appears progressive in the mdx mouse. *Neuromuscul. Disord.* **30**, 368–388 (2020).
45. C. Pascual-Morena, I. Cavero-Redondo, I. Sequi-Dominguez, E. Rodriguez-Gutierrez, M. E. Visier-Alfonso, V. Martinez-Vizcaino, Intelligence quotient-genotype association in dystrophinopathies: A systematic review and meta-analysis. *Neuropathol. Appl. Neurobiol.* **49**, e12914 (2023).
46. Y. Lou, X. Shi, G. Su, Y. Guo, L. Gao, Y. Wang, P. Miao, J. Feng, Hemizygous splicing variant in CNKSR2 results in X-linked intellectual developmental disorder. *Mol. Genet. Genomic Med.* **12**, e2389 (2024).
47. U. Aypar, E. C. Wirrell, N. L. Hoppman, CNKSR2 deletions: A novel cause of X-linked intellectual disability and seizures. *Am. J. Med. Genet. A* **167**, 1668–1670 (2015).
48. K. M. Allen, J. G. Gleeson, S. Bagrodia, M. W. Partington, J. C. MacMillan, R. A. Cerione, J. C. Mulley, C. A. Walsh, PAK3 mutation in nonsyndromic X-linked mental retardation. *Nat. Genet.* **20**, 25–30 (1998).
49. G. Pascolini, F. Gaudioso, C. Passarelli, A. Novelli, N. Di Giosaffatte, S. Majore, P. Grammatico, Clinical and molecular aspects of the neurodevelopmental disorder associated with PAK3 perturbation. *J. Mol. Neurosci.* **71**, 2474–2481 (2021).
50. D. Caudal, V. Francois, A. Lafoux, M. Ledevin, I. Anegon, C. Le Guiner, T. Larcher, C. Huchet, Characterization of brain dystrophins absence and impact in dystrophin-deficient Dmdmdx rat model. *PLOS ONE* **15**, e0230083 (2020).
51. H. Ito, R. Morishita, M. Noda, T. Ishiguro, M. Nishikawa, K. I. Nagata, The synaptic scaffolding protein CNKSR2 interacts with CYTH2 to mediate hippocampal granule cell development. *J. Biol. Chem.* **297**, 101427 (2021).
52. A. Dubos, G. Combeau, Y. Bernardinelli, J. V. Barnier, O. Hartley, H. Gaertner, B. Boda, D. Muller, Alteration of synaptic network dynamics by the intellectual disability protein PAK3. *J. Neurosci.* **32**, 519–527 (2012).
53. B. Boda, S. Alberi, I. Nikonenko, R. Node-Langlois, P. Jourdain, M. Moosmayer, L. Parisi-Jourdain, D. Muller, The mental retardation protein PAK3 contributes to synapse formation and plasticity in hippocampus. *J. Neurosci.* **24**, 10816–10825 (2004).
54. J. Meng, Y. Meng, A. Hanna, C. Janus, Z. Jia, Abnormal long-lasting synaptic plasticity and cognition in mice lacking the mental retardation gene Pak3. *J. Neurosci.* **25**, 6641–6650 (2005).
55. X. Li, V. Giri, Y. Cui, M. Yin, Z. Xian, J. Li, LncRNA FTX inhibits hippocampal neuron apoptosis by regulating miR-21-5p/SOX7 axis in a rat model of temporal lobe epilepsy. *Biochem. Biophys. Res. Commun.* **512**, 79–86 (2019).
56. G. Zhang, Y. Gao, L. Jiang, Y. Zhang, LncRNA FTX inhibits ferroptosis of hippocampal neurons displaying epileptiform discharges in vitro through the miR-142-5p/GABPB1 axis. *Neuroscience* **526**, 48–60 (2023).
57. C. Chureau, S. Chantalat, A. Romito, A. Galvani, L. Duret, P. Avner, C. Rougeulle, *Ftx* is a non-coding RNA which affects *Xist* expression and chromatin structure within the X-inactivation center region. *Hum. Mol. Genet.* **20**, 705–718 (2011).
58. H. B. Werner, E. M. Kramer-Albers, N. Strenzke, G. Saher, S. Tenzer, Y. Ohno-Iwashita, P. De Monasterio-Schrader, W. Mobius, T. Moser, I. R. Griffiths, K. A. Nave, A critical role for the cholesterol-associated proteolipids PLP and M6B in myelination of the central nervous system. *Glia* **61**, 567–586 (2013).
59. M. J. Osorio, S. A. Goldman, Neurogenetics of pelizaeus-merzbacher disease. *Handb. Clin. Neurol.* **148**, 701–722 (2018).
60. P. Aguilera, A. J. Lopez-Contreras, ATRX, a guardian of chromatin. *Trends Genet.* **39**, 505–519 (2023).
61. J. Shin, M. C. Wallingford, J. Gallant, C. Marcho, B. Jiao, M. Byron, M. Bossenz, J. B. Lawrence, S. N. Jones, J. Mager, I. Bach, RLIM is dispensable for X-chromosome inactivation in the mouse embryonic epiblast. *Nature* **511**, 86–89 (2014).
62. O. Rosspopoff, E. Cazottes, C. Huret, A. Loda, A. J. Collier, M. Casanova, P. J. Rugg-Gunn, E. Heard, J. F. Ouimette, C. Rougeulle, Species-specific regulation of *XIST* by the *JPX/FTX* orthologs. *Nucleic Acids Res.* **51**, 2177–2194 (2023).
63. K. M. Choi, J. Y. Kim, Y. Kim, Distribution of the immunoreactivity for glycoprotein M6B in the neurogenic niche and reactive glia in the injury penumbra following traumatic brain injury in mice. *Exp. Neurobiol.* **22**, 277–282 (2013).
64. M. Klugmann, M. H. Schwab, A. Puhlhofer, A. Schneider, F. Zimmermann, I. R. Griffiths, K. A. Nave, Assembly of CNS myelin in the absence of proteolipid protein. *Neuron* **18**, 59–70 (1997).
65. S. G. Kaler, ATP7A-related copper transport diseases—Emerging concepts and future trends. *Nat. Rev. Neurol.* **7**, 15–29 (2011).
66. S. Okano, Y. Makita, A. Miyamoto, G. Taketazu, K. Kimura, I. Fukuda, H. Tanaka, K. Yanagi, T. Kaname, GRIA3 p.Met661Thr variant in a female with developmental epileptic encephalopathy. *Hum. Genome Var.* **10**, 4 (2023).
67. J. Piard, J. H. Hu, P. M. Campeau, S. Rzonca, H. Van Esch, E. Vincent, M. Han, E. Rossignol, J. Castaneda, J. Chelly, C. Skinner, V. M. Kalscheuer, R. Wang, E. Lemyre, J. Kosinska, P. Stawinski, J. Bal, D. A. Hoffman, C. E. Schwartz, L. Van Maldergem, T. Wang, P. F. Worley, FRMPD4 mutations cause X-linked intellectual disability and disrupt dendritic spine morphogenesis. *Hum. Mol. Genet.* **27**, 589–600 (2018).
68. G. J. Ramakers, D. Wolfer, G. Rosenberger, K. Kuchenbecker, H. J. Kreienkamp, J. Prange-Kiel, G. Rune, K. Richter, K. Langnaese, S. Masneuf, M. R. Bosl, K. D. Fischer, H. J. Krugers, H. P. Lipp, E. van Galen, K. Kutsche, Dysregulation of Rho GTPases in the αPix/Arhgef6 mouse model of X-linked intellectual disability is paralleled by impaired structural and synaptic plasticity and cognitive deficits. *Hum. Mol. Genet.* **21**, 268–286 (2012).
69. Z. Tumer, L. B. Moller, Menkes disease. *Eur. J. Hum. Genet.* **18**, 511–518 (2010).

70. U. Moog, K. Kutsche, "CASK disorders" in *GeneReviews*®, M. P. Adam, J. Feldman, G. M. Mirzaa, R. A. Pagon, S. E. Wallace, L. J. H. Bean, K. W. Gripp, A. Amemiya, Eds. (University of Washington, 1993).
71. M. Morleo, B. Franco, "Microphthalmia with linear skin defects syndrome" in *GeneReviews*®, M. P. Adam, J. Feldman, G. M. Mirzaa, R. A. Pagon, S. E. Wallace, L. J. H. Bean, K. W. Gripp, A. Amemiya, Eds. (University of Washington, 1993).
72. U. Hehr, G. Uyanik, L. Aigner, S. Couillard-Despres, J. Winkler, "DCX-related disorders" in *GeneReviews*®, M. P. Adam, J. Feldman, G. M. Mirzaa, R. A. Pagon, S. E. Wallace, L. J. H. Bean, K. W. Gripp, A. Amemiya, Eds. (University of Washington, 1993).
73. B. Jia, L. Huang, Y. Chen, S. Liu, C. Chen, K. Xiong, L. Song, Y. Zhou, X. Yang, M. Zhong, A novel contiguous deletion involving NDP, MAOB and EFHC2 gene in a patient with familial Norrie disease: Bilateral blindness and leucocoria without other deficits. *J. Genet.* **96**, 1015–1020 (2017).
74. J. F. Quesada-Espinosa, L. Garzon-Lorenzo, J. M. Lezana-Rosales, M. J. Gomez-Rodriguez, M. T. Sanchez-Calvin, C. Palma-Milla, I. Gomez-Manjon, I. Hidalgo-Mayoral, R. Perez de la Fuente, A. Arteche-Lopez, M. I. Alvarez-Mora, A. Camacho-Salas, J. Cruz-Rojo, I. Lazaro-Rodriguez, M. Morales-Conejo, N. Nunez-Enamorado, A. Bustamante-Aragones, R. S. de Las Heras, M. A. Gomez-Cano, P. Ramos-Gomez, O. Sierra-Tomillo, A. Juarez-Rufian, J. Gallego-Merlo, L. Rausell-Sanchez, M. Moreno-Garcia, J. S. Del Pozo, First female with Allan-Herndon-Dudley syndrome and partial deletion of X-inactivation center. *Neurogenetics* **22**, 343–346 (2021).
75. B. Rinaldi, A. Bayat, L. G. Zachariassen, J. H. Sun, Y. H. Ge, D. Zhao, K. Bonde, L. H. Madsen, I. A. A. Awad, D. Bagiran, A. Sbeih, S. M. Shah, S. El-Sayed, S. M. Lyngby, M. G. Pedersen, C. Stenum-Berg, L. C. Walker, I. Krey, A. Delahaye-Duriez, L. T. Emrick, K. Sully, C. N. Murali, L. C. Burrage, J. A. P. Gonzalez, M. Parnes, J. Friedman, B. Isidor, J. Lefranc, S. Redon, D. Heron, C. Mignot, B. Keren, M. Fradin, C. Dubourg, S. Mercier, T. Besnard, B. Cogne, W. Deb, C. Rivier, D. Milani, M. F. Bedeschi, C. Di Napoli, F. Grilli, P. Marchisio, S. Koudijs, D. Veenma, E. Argilli, S. A. Lynch, P. Y. B. Au, F. E. A. Valenzuela, C. Brown, D. Masser-Frye, M. Jones, L. P. Romero, W. L. Li, E. Thorpe, L. Hecher, J. Johannsen, J. Denecke, V. McNiven, A. Szuto, E. Wakeling, V. Cruz, V. Sency, H. Wang, J. Piard, F. Kortum, T. Herget, T. Bierhals, A. Condell, B. Ben-Zeev, S. Kaur, J. Christodoulou, A. Piton, C. Zweier, C. Kraus, A. Micalizzi, M. Trivisano, N. Specchio, G. Lesca, R. S. Moller, Z. Tumer, M. Musgaard, B. Gerard, J. R. Lemke, Y. S. Shi, A. S. Kristensen, Gain-of-function and loss-of-function variants in GRIA3 lead to distinct neurodevelopmental phenotypes. *Brain* **147**, 1837–1855 (2024).
76. M. I. Boyle, C. Jespersgaard, K. Brondum-Nielsen, A. M. Bisgaard, Z. Tumer, Cornelia de Lange syndrome. *Clin. Genet.* **88**, 1–12 (2015).
77. A. A. Gholkar, S. Senese, Y. C. Lo, E. Vides, E. Contreras, E. Hodara, J. Capri, J. P. Whitelegge, J. Z. Torres, The X-linked-intellectual-disability-associated Ubiquitin ligase Mid2 interacts with astrin and regulates astrin levels to promote cell division. *Cell Rep.* **14**, 180–188 (2016).
78. J. C. Lieske, D. S. Milliner, L. Beara-Lasic, P. Harris, A. Cogal, E. Abrash, "Dent disease" in *GeneReviews*®, M. P. Adam, J. Feldman, G. M. Mirzaa, R. A. Pagon, S. E. Wallace, L. J. H. Bean, K. W. Gripp, A. Amemiya, Eds. (University of Washington, 1993).
79. V. M. Pravata, M. Omelkova, M. P. Stavridis, C. M. Desbiens, H. M. Stephen, D. J. Lefeber, J. Gecz, M. Gundogdu, K. Ounap, S. Joss, C. E. Schwartz, L. Wells, D. M. F. van Aalten, An intellectual disability syndrome with single-nucleotide variants in O-GlcNAc transferase. *Eur. J. Hum. Genet.* **28**, 706–714 (2020).
80. C. Castillon, L. Gonzalez, F. Domenichini, S. Guyon, K. Da Silva, C. Durand, P. Lestaevel, C. Vaillend, S. Laroche, J. V. Barnier, R. Poirier, The intellectual disability PAK3 R67C mutation impacts cognitive functions and adult hippocampal neurogenesis. *Hum. Mol. Genet.* **29**, 1950–1968 (2020).
81. N. Sahajpal, C. Ziats, A. Chaubey, B. R. DuPont, F. Abidi, C. E. Schwartz, R. E. Stevenson, Clinical findings in individuals with duplication of genes associated with X-linked intellectual disability. *Clin. Genet.* **105**, 173–184 (2024).
82. R. C. Rogers, F. E. Abidi, "RPS6KA3-related intellectual disability" in *GeneReviews*®, M. P. Adam, J. Feldman, G. M. Mirzaa, R. A. Pagon, S. E. Wallace, L. J. H. Bean, K. W. Gripp, A. Amemiya, Eds. (University of Washington, 1993).
83. B. Poreau, F. Ramond, R. Harbuz, V. Satre, C. Barro, C. Vettier, V. Adouard, J. Thevenon, P. S. Jouk, C. Coutton, R. Touraine, K. Dieterich, Xq22.3q23 microdeletion harboring TMEM164 and AMMECR1 genes: Two case reports confirming a recognizable phenotype with short stature, midface hypoplasia, intellectual delay, and elliptocytosis. *Am. J. Med. Genet. A* **179**, 650–654 (2019).
84. S. Bassani, L. A. Cingolani, P. Valnegri, A. Folci, J. Zapata, A. Gianfelice, C. Sala, Y. Goda, M. Passafaro, The X-linked intellectual disability protein TSPAN7 regulates excitatory synapse development and AMPAR trafficking. *Neuron* **73**, 1143–1158 (2012).
85. Y. Huang, S. Huang, S. M. Lam, Z. Liu, G. Shui, Y. Q. Zhang, Acsl, the *Drosophila* ortholog of intellectual-disability-related ACSL4, inhibits synaptic growth by altered lipids. *J. Cell Sci.* **129**, 4034–4045 (2016).
86. A. Dubos, A. Castells-Nobau, H. Meziane, M. A. Oortveld, X. Houbaert, G. Iacono, C. Martin, C. Mittelhaeuser, V. Lalanne, J. M. Kramer, A. Bhukel, C. Quentin, J. Slabbert, P. Verstreken, S. J. Sigrist, N. Messaddeq, M. C. Birling, M. Selloum, H. G. Stunnenberg, Y. Humeau, A. Schenck, Y. Herault, Conditional depletion of intellectual disability and Parkinsonism candidate gene ATP6AP2 in fly and mouse induces cognitive impairment and neurodegeneration. *Hum. Mol. Genet.* **24**, 6736–6755 (2015).
87. R. E. Stevenson, "Alpha-thalassemia X-linked intellectual disability syndrome" in *GeneReviews*®, M. P. Adam, J. Feldman, G. M. Mirzaa, R. A. Pagon, S. E. Wallace, L. J. H. Bean, K. W. Gripp, A. Amemiya, Eds. (University of Washington, 1993).
88. A. Shukla, K. M. Girisha, P. H. Somashekar, S. Nampoothiri, R. McClellan, H. J. Vernon, Variants in the transcriptional corepressor BCORL1 are associated with an X-linked disorder of intellectual disability, dysmorphic features, and behavioral abnormalities. *Am. J. Med. Genet. A* **179**, 870–874 (2019).
89. N. D. Rendtorff, H. G. Karstensen, M. Lodahl, J. Tolmie, C. McWilliam, M. Bak, N. Tommerup, L. Nazaryan-Petersen, H. Kunst, M. Wong, S. Joss, V. Carelli, L. Tranebjaerg, Identification and analysis of deletion breakpoints in four Mohr-Tranebjaerg syndrome (MTS) patients. *Sci. Rep.* **12**, 14959 (2022).
90. S. Liang, N. Jiang, S. Li, X. Jiang, D. Yu, A maternally inherited 8.05 Mb Xq21 deletion associated with choroideremia, deafness, and mental retardation syndrome in a male patient. *Mol. Cytogenet.* **10**, 23 (2017).
91. L. S. Blok, E. Madsen, J. Juusola, C. Gilissen, D. Baralle, M. R. Reijnders, H. Venselaar, C. Helsmoortel, M. T. Cho, A. Hoischen, L. E. Vissers, T. S. Koemans, W. Wissink-Lindhout, E. E. Eichler, C. Romano, H. Van Esch, C. Stumpel, M. Vreeburg, E. Smeets, K. Oberndorff, B. W. van Bon, M. Shaw, J. Gecz, E. Haan, M. Bienek, C. Jensen, B. L. Loeys, A. Van Dijck, A. M. Innes, H. Racher, S. Vermeer, N. Di Donato, A. Rump, K. Tatton-Brown, M. J. Parker, A. Henderson, S. A. Lynch, A. Fryer, A. Ross, P. Vasudevan, U. Kini, R. Newbury-Ecob, K. Chandler, A. Male, D. D. D. Study, S. Dijkstra, J. Schieving, J. Giltay, K. L. van Gassen, J. Schuurs-Hoeijmakers, P. L. Tan, I. Pediaditakis, S. A. Haas, K. Retterer, P. Reed, K. G. Monaghan, E. Haverfield, M. Natowicz, A. Myers, M. C. Kruer, Q. Stein, K. A. Strauss, K. W. Brigatti, K. Keating, B. K. Burton, K. H. Kim, J. Charrow, J. Norman, A. Foster-Barber, A. D. Kline, A. Kimball, E. Zackai, M. Harr, J. Fox, J. McLaughlin, K. Lindstrom, K. M. Haude, K. van Roozendaal, H. Brunner, W. K. Chung, R. F. Kooy, R. Pfundt, V. Kalscheuer, S. G. Mehta, N. Katsanis, T. Kleefstra, Mutations in DDX3X are a common cause of unexplained intellectual disability with gender-specific effects on Wnt signaling. *Am. J. Hum. Genet.* **97**, 343–352 (2015).
92. A. Mir, Y. Song, H. Lee, H. Khanahmad, E. Khorram, J. Nasiri, M. A. Tabatabaiefar, Whole exome sequencing revealed variants in four genes underlying X-linked intellectual disability in four Iranian families: Novel deleterious variants and clinical features with the review of literature. *BMC Med. Genomics* **16**, 239 (2023).
93. S. L. Chiu, G. H. Diering, B. Ye, K. Takamiya, C. M. Chen, Y. Jiang, T. Niranjan, C. E. Schwartz, T. Wang, R. L. Huganir, GRASP1 regulates synaptic plasticity and learning through endosomal recycling of AMPA receptors. *Neuron* **93**, 1405–1419.e8 (2017).
94. N. S. Levy, G. K. E. Umanah, E. J. Rogers, R. Jada, O. Lache, A. P. Levy, IQSEC2-associated intellectual disability and autism. *Int. J. Mol. Sci.* **20**, 3038 (2019).
95. R. S. D'Souza, L. Law, *Danon Disease* (StatPearls, 2024).
96. T. Brunet, K. McWalter, K. Mayerhanser, G. M. Anbouba, A. Armstrong-Javors, I. Bader, E. Baugh, A. Begtrup, C. P. Bupp, B. L. Callewaert, A. Cereda, M. A. Cousin, J. C. D. R. Jimenez, L. Demmer, N. R. Dsouza, N. Fleischer, R. H. Gavrilova, S. Ghate, E. Graf, A. Green, S. R. Green, M. Iascone, A. Kdissa, D. Klee, E. W. Klee, E. Lancaster, K. Lindstrom, J. A. Mayr, M. McEntagart, N. J. L. Meeks, D. Mittag, H. Moore, A. K. Olsen, D. Ortiz, G. Parsons, L. D. M. Pena, R. E. Person, S. Punj, G. A. Ramos-Rivera, M. J. G. Sacoto, G. B. Schaefer, R. E. Schnur, T. M. Scott, D. A. Scott, C. R. Serbinski, V. Shashi, V. M. Siu, B. F. Stadheim, J. A. Sullivan, J. Svantnerova, L. Velsher, D. S. Wargowski, I. M. Wentzensen, D. Wieczorek, J. Winkelmann, P. Yap, M. Zech, M. T. Zimmermann, T. Meitinger, F. Distelmaier, M. Wagner, Defining the genotypic and phenotypic spectrum of X-linked MSL3-related disorder. *Genet. Med.* **23**, 384–395 (2021).
97. Y. Wu, G. J. Lyon, NAA10-related syndrome. *Exp. Mol. Med.* **50**, 1–10 (2018).
98. T. A. Nguyen, A. W. Lehr, K. W. Roche, Neuroligins and neurodevelopmental disorders: X-linked genetics. *Front. Synaptic. Neurosci.* **12**, 33 (2020).
99. U. Lichter-Konecki, L. Caldovic, H. Morizono, K. Simpson, N. Ah Mew, E. MacLeod, "Ornithine transcarbamylase deficiency" in *GeneReviews*®, M. P. Adam, J. Feldman, G. M. Mirzaa, R. A. Pagon, S. E. Wallace, L. J. H. Bean, K. W. Gripp, A. Amemiya, Eds. (University of Washington, 1993).
100. K. Saida, T. Fukuda, D. A. Scott, T. Sengoku, K. Ogata, A. Nicosia, A. Hernandez-Garcia, S. R. Lalani, M. S. Azamian, H. Streff, P. Liu, H. Dai, T. Mizuguchi, S. Miyatake, M. Asahina, T. Ogata, N. Miyake, N. Matsumoto, OTUD5 variants associated with X-linked intellectual disability and congenital malformation. *Front. Cell. Dev. Biol.* **9**, 631428 (2021).
101. Z. Liu, B. Xin, I. N. Smith, V. Sency, J. Szekely, A. Alkelai, A. Shuldiner, S. Efthymiou, F. Rajabi, S. Coury, C. A. Brownstein, S. Rudnik-Schoneborn, A. L. Bruel, J. Thevenon, S. Zeidler, P. Jayakar, A. Schmidt, K. Cremer, H. Engels, S. O. Peters, M. S. Zaki, R. Duan, C. Zhu, Y. Xu, C. Gao, T. Sepulveda-Morales, R. Maroofian, I. A. Alkhawaja, M. Khawaja, H. Alhalasah, H. Houlden, J. A. Madden, V. Turchetti, D. Marafi, P. B. Agrawal, U. Schatz, A. Rotenberg, J. Rotenberg, G. M. S. Mancini, S. Bakhtiari, M. Kruer, I. Thiffault, S. Hirsch, M. Hempel, L. G. Stuhn, T. B. Haack, J. E. Posey, J. R. Lupski, H. Lee, N. B. Sarn, C. Eng, C. Gonzaga-Jauregui, B. Zhang, H. Wang, Hemizygous variants in protein phosphatase 1 regulatory subunit 3F (PPP1R3F) are associated with a neurodevelopmental disorder

characterized by developmental delay, intellectual disability and autistic features. *Hum. Mol. Genet.* **32**, 2981–2995 (2023).
102. H. Hu, S. A. Haas, J. Chelly, H. Van Esch, M. Raynaud, A. P. de Brouwer, S. Weinert, G. Froyen, S. G. Frints, F. Laumonnier, T. Zemojtel, M. I. Love, H. Richard, A. K. Emde, M. Bienek, C. Jensen, M. Hambrock, U. Fischer, C. Langnick, M. Feldkamp, W. Wissink-Lindhout, N. Lebrun, L. Castelnau, J. Rucci, R. Montjean, O. Dorseuil, P. Billuart, T. Stuhlmann, M. Shaw, M. A. Corbett, A. Gardner, S. Willis-Owen, C. Tan, K. L. Friend, S. Belet, K. E. van Roozendaal, M. Jimenez-Pocquet, M. P. Moizard, N. Ronce, R. Sun, S. O'Keeffe, R. Chenna, A. van Bommel, J. Goke, A. Hackett, M. Field, L. Christie, J. Boyle, E. Haan, J. Nelson, G. Turner, G. Baynam, G. Gillessen-Kaesbach, U. Muller, D. Steinberger, B. Budny, M. Badura-Stronka, A. Latos-Bielenska, L. B. Ousager, P. Wieacker, G. R. Criado, M. L. Bondeson, G. Anneren, A. Dufke, M. Cohen, L. Van Maldergem, C. Vincent-Delorme, B. Echenne, B. Simon-Bouy, T. Kleefstra, M. Willemsen, J. P. Fryns, K. Devriendt, R. Ullmann, M. Vingron, K. Wrogemann, T. F. Wienker, A. Tzschach, H. van Bokhoven, J. Gecz, T. J. Jentsch, W. Chen, H. H. Ropers, V. M. Kalscheuer, X-exome sequencing of 405 unresolved families identifies seven novel intellectual disability genes. *Mol. Psychiatry* **21**, 133–148 (2016).
103. C. Sarret, I. Oliver Petit, D. Tonduti, "Allan-Herndon-Dudley syndrome" in *GeneReviews®*, M. P. Adam, J. Feldman, G. M. Mirzaa, R. A. Pagon, S. E. Wallace, L. J. H. Bean, K. W. Gripp, A. Amemiya, Eds. (University of Washington, 1993).
104. C. Barba, I. Blumcke, M. R. Winawer, T. Hartlieb, H. C. Kang, L. Grisotto, M. Chipaux, C. G. Bien, B. Hermanovska, B. E. Porter, H. G. W. Lidov, V. Cetica, F. G. Woermann, J. A. Lopez-Rivera, P. D. Canoll, I. Mader, L. D'Incerti, S. Baldassari, E. Yang, A. Gaballa, H. Vogel, B. Straka, L. Macconi, T. Polster, G. A. Grant, L. Krskova, H. J. Shin, A. Ko, P. B. Crino, P. Krsek, J. H. Lee, D. Lal, S. Baulac, A. Poduri, R. Guerrini, SLC35A2 Study Group, Clinical features, neuropathology, and surgical outcome in patients with refractory epilepsy and brain somatic variants in the SLC35A2 gene. *Neurology* **100**, e528–e542 (2023).
105. W. Khayat, A. Hackett, M. Shaw, A. Ilie, T. Dudding-Byth, V. M. Kalscheuer, L. Christie, M. A. Corbett, J. Juusola, K. L. Friend, B. M. Kirmse, J. Gecz, M. Field, J. Orlowski, A recurrent missense variant in SLC9A7 causes nonsyndromic X-linked intellectual disability with alteration of Golgi acidification and aberrant glycosylation. *Hum. Mol. Genet.* **28**, 598–614 (2019).
106. F. Lopes, M. Barbosa, A. Ameur, G. Soares, J. de Sa, A. I. Dias, G. Oliveira, P. Cabral, T. Temudo, E. Calado, I. F. Cruz, J. P. Vieira, R. Oliveira, S. Esteves, S. Sauer, I. Jonasson, A. C. Syvanen, U. Gyllensten, D. Pinto, P. Maciel, Identification of novel genetic causes of Rett syndrome-like phenotypes. *J. Med. Genet.* **53**, 190–199 (2016).
107. Y. R. Lee, M. G. Thomas, A. Roychaudhury, C. Skinner, G. Maconachie, M. Crosier, H. Horak, C. S. Constantinescu, T. I. Choi, J. J. Kyung, T. Wang, B. Ku, B. N. Chodirker, M. F. Hammer, I. Gottlob, W. H. J. Norton, A. E. Chudley, C. E. Schwartz, C. H. Kim, Eye movement defects in KO zebrafish reveals *SRPK3* as a causative gene for an X-linked intellectual disability. *Res. Sq.* (2023).
108. R. Kumar, M. A. Corbett, B. W. Van Bon, A. Gardner, J. A. Woenig, L. A. Jolly, E. Douglas, K. Friend, C. Tan, H. Van Esch, M. Holvoet, M. Raynaud, M. Field, M. Leffler, B. Budny, M. Wisniewska, M. Badura-Stronka, A. Latos-Bielenska, J. Batanian, J. A. Rosenfeld, L. Basel-Vanagaite, C. Jensen, M. Bienek, G. Froyen, R. Ullmann, H. Hu, M. I. Love, S. A. Haas, P. Stankiewicz, S. W. Cheung, A. Baxendale, J. Nicholl, E. M. Thompson, E. Haan, V. M. Kalscheuer, J. Gecz, Increased STAG2 dosage defines a novel cohesinopathy with intellectual disability and behavioral problems. *Hum. Mol. Genet.* **24**, 7171–7181 (2015).
109. D. Lehalle, P. Vabres, A. Sorlin, T. Bierhals, M. Avila, V. Carmignac, M. Chevarin, E. Torti, Y. Abe, T. Bartolomaeus, J. Clayton-Smith, B. Cogne, I. Cusco, L. Duplomb, E. De Bont, Y. Duffourd, F. Duijkers, O. Elpeleg, A. Fattal, D. Genevieve, M. J. Guillen Sacoto, A. Guimier, D. J. Harris, M. Hempel, B. Isidor, T. Jouan, P. Kuentz, E. Koshimizu, K. Lichtenbelt, V. Loik Ramey, M. Maik, S. Miyakate, Y. Murakami, L. Pasquier, H. Pedro, L. Simone, K. Sondergaard-Schatz, J. St-Onge, J. Thevenon, I. Valenzuela, R. Abou Jamra, K. van Gassen, M. M. van Haelst, S. van Koningsbruggen, E. Verdura, C. Whelan Habela, P. Zacher, J. B. Riviere, C. Thauvin-Robinet, J. Betschinger, L. Faivre, De novo mutations in the X-linked TFE3 gene cause intellectual disability with pigmentary mosaicism and storage disorder-like features. *J. Med. Genet.* **57**, 808–819 (2020).
110. C. Mignon-Ravix, P. Cacciagli, N. Choucair, C. Popovici, C. Missirian, M. Milh, A. Megarbane, T. Busa, S. Julia, N. Girard, C. Badens, S. Sigaudy, N. Philip, L. Villard, Intragenic rearrangements in X-linked intellectual deficiency: Results of a-CGH in a series of 54 patients and identification of TRPC5 and KLHL15 as potential XLID genes. *Am. J. Med. Genet. A* **164A**, 1991–1997 (2014).
111. N. Vasli, I. Ahmed, K. Mittal, M. Ohadi, A. Mikhailov, M. A. Rafiq, A. Bhatti, M. T. Carter, D. M. Andrade, M. Ayub, J. B. Vincent, P. John, Identification of a homozygous missense mutation in LRP2 and a hemizygous missense mutation in TSPYL2 in a family with mild intellectual disability. *Psychiatr. Genet.* **26**, 66–73 (2016).
112. L. A. Jolly, C. C. Homan, R. Jacob, S. Barry, J. Gecz, The UPF3B gene, implicated in intellectual disability, autism, ADHD and childhood onset schizophrenia regulates neural progenitor cell behaviour and neuronal outgrowth. *Hum. Mol. Genet.* **22**, 4673–4687 (2013).
113. S. Kury, J. Zhang, T. Besnard, A. Caro-Llopis, X. Zeng, S. M. Robert, S. S. Josiah, E. Kiziltug, A. S. Denomme-Pichon, B. Cogne, A. J. Kundishora, L. T. Hao, H. Li, R. E. Stevenson, R. J. Louie, W. Deb, E. Torti, V. Vignard, K. McWalter, F. L. Raymond, F. Rajabi, E. Ranza, D. Grozeva, S. A. Coury, X. Blanc, E. Brischoux-Boucher, B. Keren, K. Ounap, K. Reinson, P. Ilves, I. M. Wentzensen, E. E. Barr, S. H. Guihard, P. Charles, E. G. Seaby, K. G. Monaghan, M. Rio, Y. van Bever, M. van Slegtenhorst, W. K. Chung, A. Wilson, D. Quinquis, F. Breheret, K. Retterer, P. Lindenbaum, E. Scalais, L. Rhodes, K. Stouffs, E. M. Pereira, S. M. Berger, S. S. Milla, A. B. Jaykumar, M. H. Cobb, S. Panchagnula, P. Q. Duy, M. Vincent, S. Mercier, B. Gilbert-Dussardier, X. Le Guillou, S. Audebert-Bellanger, S. Odent, S. Schmitt, P. Boisseau, D. Bonneau, A. Toutain, E. Colin, L. Pasquier, R. Redon, A. Bouman, J. A. Rosenfeld, M. J. Friez, H. Perez-Pena, S. R. Akhtar Rizvi, S. Haider, S. E. Antonarakis, C. E. Schwartz, F. Martinez, S. Bezieau, K. T. Kahle, B. Isidor, Rare pathogenic variants in WNK3 cause X-linked intellectual disability. *Genet. Med.* **24**, 1941–1951 (2022).
114. M. Parisi, I. Glass, "Joubert syndrome" in *GeneReviews®*, M. P. Adam, J. Feldman, G. M. Mirzaa, R. A. Pagon, S. E. Wallace, L. J. H. Bean, K. W. Gripp, A. Amemiya, Eds. (University of Washington, 1993).
115. R. Singh, D. Samanta, *Pelizaeus-Merzbacher Disease* (StatPearls, 2024).
116. B. A. Strange, M. P. Witter, E. S. Lein, E. I. Moser, Functional organization of the hippocampal longitudinal axis. *Nat. Rev. Neurosci.* **15**, 655–669 (2014).
117. J. L. Bizon, T. C. Foster, G. E. Alexander, E. L. Glisky, Characterizing cognitive aging of working memory and executive function in animal models. *Front. Aging Neurosci.* **4**, 19 (2012).
118. L. Sun, Z. Wang, T. Lu, T. A. Manolio, A. D. Paterson, eXclusionarY: 10 years later, where are the sex chromosomes in GWASs? *Am. J. Hum. Genet.* **110**, 903–912 (2023).
119. B. Chen, R. V. Craiu, L. J. Strug, L. Sun, The X factor: A robust and powerful approach to X-chromosome-inclusive whole-genome association studies. *Genet. Epidemiol.* **45**, 694–709 (2021).
120. J. Le Borgne, L. Gomez, S. Heikkinen, N. Amin, S. Ahmad, S. H. Choi, J. Bis, B. Grenier-Boley, O. G. Rodriguez, L. Kleineidam, J. Young, K. P. Tripathi, L. Wang, A. Varma, S. van der Lee, V. Damotte, I. de Rojas, S. Palmal, EADB, GR@ACE, DEGESCO, EADI, GERAD, DemGene, FinnGen, ADGC, CHARGE, R. Lipton, E. Reiman, A. M. Kee, P. De Jager, W. Bush, S. Small, A. Levey, A. Saykin, T. Foroud, M. Albert, B. Hyman, R. Petersen, S. Younkin, M. Sano, T. Wisniewski, R. Vassar, J. Schneider, V. Henderson, E. Roberson, C. De Carli, F. L. Ferla, J. Brewer, R. Swerdlow, L. Van Eldik, K. Hamilton-Nelson, H. Paulson, A. Naj, O. Lopez, H. Chui, P. Crane, T. Grabowski, W. Kukull, S. Asthana, S. Craft, S. Strittmatter, C. Cruchaga, J. Leverenz, A. Goate, M Ilyas Kamboh, P. St George-Hyslop, O. Valladares, A. Kuzma, L. Cantwell, M. Riemenschneider, J. Morris, S. Slifer, C. Dalmasso, A. Castillo, F. Küçükali, O. Peters, A. Schneider, M. Dichgans, D. Rujescu, N. Scherbaum, J. Deckert, S. Riedel-Heller, L. Hausner, L. Molina-Porcel, E. Düzel, T. Grimmer, J. Wiltfang, S. Heilmann-Heimbach, S. Moebus, T. Tegos, N. Scarmeas, O. Dols-Icardo, F. Moreno, J. Pérez-Tur, M. J. Bullido, P. Pastor, R. Sánchez-Valle, V. Álvarez, M. Boada, P. García-González, R. Puerta, P. Mir, L. M. Real, G. Piñol-Ripoll, J. M. García-Alberca, J. L. Royo, E. Rodriguez-Rodriguez, H. Soininen, A. de Mendonça, S. Mehrabian, L. Traykov, J. Hort, M. Vyhnalek, J. Q. Thomassen, Y. A. L. Pijnenburg, H. Holstege, J. van Swieten, I. Ramakers, F. Verhey, P. Scheltens, C. Graff, G. Papenberg, V. Giedraitis, R. Ghidoni, V. Fernandez, P. G. Kehoe, R. Frikke-Schmidt, M. Tsolaki, P. Sánchez-Juan, K. Sleegers, M. Ingelsson, J. Haines, L. Farrer, R. Mayeux, L.-S. Wang, R. Sims, A. DeStefano, G. D. Schellenberg, S. Seshadri, P. Amouyel, J. Williams, W. van der Flier, A. Ramirez, M. Pericak-Vance, O. Andreassen, C. Van Duijn, M. Hiltunen, A. Ruiz, J. Dupuis, E. Martin, J.-C. Lambert, B. Kunkle, C. Bellenguez, X chromosome-wide association study for Alzheimer's disease. medRxiv 2024.05.02.24306739 [Preprint] (2024). https://doi.org/10.1101/2024.05.02.24306739.
121. E. Simmonds, G. Leonenko, U. Yaman, E. Bellou, A. Myers, K. Morgan, K. Brookes, J. Hardy, D. Salih, V. Escott-Price, Chromosome X-wide association study in case control studies of pathologically confirmed Alzheimer's disease in a European population. *Transl. Psychiatry* **14**, 358 (2024).
122. M. E. Belloy, Y. Le Guen, I. Stewart, K. Williams, J. Herz, R. Sherva, R. Zhang, V. Merritt, M. S. Panizzon, R. L. Hauger, J. M. Gaziano, M. Logue, V. Napolioni, M. D. Greicius, Role of the X chromosome in alzheimer disease genetics. *JAMA Neurol.* **81**, 1032–1042 (2024).
123. M. F. Lyon, Gene action in the X-chromosome of the mouse (Mus musculus L.). *Nature* **190**, 372–373 (1961).
124. T. Jegu, E. Aeby, J. T. Lee, The X chromosome in space. *Nat. Rev. Genet.* **18**, 377–389 (2017).
125. J. T. Lee, M. S. Bartolomei, X-inactivation, imprinting, and long noncoding RNAs in health and disease. *Cell* **152**, 1308–1323 (2013).
126. R. M. Boumil, J. T. Lee, Forty years of decoding the silence in X-chromosome inactivation. *Hum. Mol. Genet.* **10**, 2225–2232 (2001).
127. R. M. Malcore, S. Kalantry, A comparative analysis of mouse imprinted and random X-chromosome inactivation. *Epigenomes* **8**, 8 (2024).
128. C. M. Disteche, J. B. Berletch, X-chromosome inactivation and escape. *J. Genet.* **94**, 591–599 (2015).
129. E. Heard, C. Rougeulle, Digging into X chromosome inactivation. *Science* **374**, 942–943 (2021).
130. L. Carrel, C. J. Brown, When the Lyon(ized chromosome) roars: Ongoing expression from an inactive X chromosome. *Philos. Trans. R. Soc. Lond. B Biol. Sci.* **372**, 20160355 (2017).

131. M. Wang, F. Lin, K. Xing, L. Liu, Random X-chromosome inactivation dynamics in vivo by single-cell RNA sequencing. *BMC Genomics* **18**, 90 (2017).
132. H. Mohammed, I. Hernando-Herraez, A. Savino, A. Scialdone, I. Macaulay, C. Mulas, T. Chandra, T. Voet, W. Dean, J. Nichols, J. C. Marioni, W. Reik, Single-cell landscape of transcriptional heterogeneity and cell fate decisions during mouse early gastrulation. *Cell Rep.* **20**, 1215–1228 (2017).
133. A. Keniry, M. E. Blewitt, Studying X chromosome inactivation in the single-cell genomic era. *Biochem. Soc. Trans.* **46**, 577–586 (2018).
134. G. Chen, J. P. Schell, J. A. Benitez, S. Petropoulos, M. Yilmaz, B. Reinius, Z. Alekseenko, L. Shi, E. Hedlund, F. Lanner, R. Sandberg, Q. Deng, Single-cell analyses of X Chromosome inactivation dynamics and pluripotency during differentiation. *Genome Res.* **26**, 1342–1354 (2016).
135. H. Marks, H. H. Kerstens, T. S. Barakat, E. Splinter, R. A. Dirks, G. van Mierlo, O. Joshi, S. Y. Wang, T. Babak, C. A. Albers, T. Kalkan, A. Smith, A. Jouneau, W. de Laat, J. Gribnau, H. G. Stunnenberg, Dynamics of gene silencing during X inactivation using allele-specific RNA-seq. *Genome Biol.* **16**, 149 (2015).
136. K. Wainer Katsir, M. Linial, Human genes escaping X-inactivation revealed by single cell expression data. *BMC Genomics* **20**, 201 (2019).
137. M. Garieri, G. Stamoulis, X. Blanc, E. Falconnet, P. Ribaux, C. Borel, F. Santoni, S. E. Antonarakis, Extensive cellular heterogeneity of X inactivation revealed by single-cell allele-specific expression in human fibroblasts. *Proc. Natl. Acad. Sci. U.S.A.* **115**, 13015–13020 (2018).
138. Y. Tomofuji, R. Edahiro, K. Sonehara, Y. Shirai, K. H. Kock, Q. S. Wang, S. Namba, J. Moody, Y. Ando, A. Suzuki, T. Yata, K. Ogawa, T. Naito, H. Namkoong, Q. X. Xuan Lin, E. V. Buyamin, L. M. Tan, R. Sonthalia, K. Y. Han, H. Tanaka, H. Lee, Asian Immune Diversity Atlas (AIDA) Network, Japan COVID-19 Task Force, Biobank Japan Project, T. Okuno, B. Liu, K. Matsuda, K. Fukunaga, H. Mochizuki, W. Y. Park, K. Yamamoto, C. C. Hon, J. W. Shin, S. Prabhakar, A. Kumanogoh, Y. Okada, Quantification of escape from X chromosome inactivation with single-cell omics data reveals heterogeneity across cell types and tissues. *Cell Genom.* **4**, 100625 (2024).
139. A. K. San Roman, A. K. Godfrey, H. Skaletsky, D. W. Bellott, A. F. Groff, H. L. Harris, L. V. Blanton, J. F. Hughes, L. Brown, S. Phou, A. Buscetta, P. Kruszka, N. Banks, A. Dutra, E. Pak, P. C. Lasutschinkow, C. Keen, S. M. Davis, N. R. Tartaglia, C. Samango-Sprouse, M. Muenke, D. C. Page, The human inactive X chromosome modulates expression of the active X chromosome. *Cell Genom.* **3**, 100259 (2023).
140. R. Aspinall, Longevity and the immune response. *Biogerontology* **1**, 273–278 (2000).
141. C. Lopez-Lee, L. Kodama, L. Fan, M. Y. Wong, N. R. Foxe, L. Jiaz, F. Yu, P. Ye, J. Zhu, K. Norman, E. R. Torres, R. D. Kim, G. A. Mousa, D. Dubal, S. Liddelow, W. Luo, L. Gan, Sex chromosomes and gonads shape the sex-biased transcriptomic landscape in Tlr7-mediated demyelination during aging. bioRxiv 2023.09.19.558439 [Preprint] (2023). https://doi.org/10.1101/2023.09.19.558439.
142. Y. Itoh, L. C. Golden, N. Itoh, M. A. Matsukawa, E. Ren, V. Tse, A. P. Arnold, R. R. Voskuhl, The X-linked histone demethylase Kdm6a in CD4+ T lymphocytes modulates autoimmunity. *J. Clin. Invest.* **129**, 3852–3863 (2019).
143. Y. Yan, X. Wang, D. Chaput, M. K. Shin, Y. Koh, L. Gan, A. A. Pieper, J. A. Woo, D. E. Kang, X-linked ubiquitin-specific peptidase 11 increases tauopathy vulnerability in women. *Cell* **185**, 3913–3930.e19 (2022).
144. F. Xie, P. Liang, H. Fu, J. C. Zhang, J. Chen, Effects of normal aging on myelin sheath ultrastructures in the somatic sensorimotor system of rats. *Mol. Med. Rep.* **10**, 459–466 (2014).
145. A. Peters, C. Sethares, Is there remyelination during aging of the primate central nervous system? *J. Comp. Neurol.* **460**, 238–254 (2003).
146. E. C. Sams, Oligodendrocytes in the aging brain. *Neuronal Signal.* **5**, NS20210008 (2021).
147. A. L. Graciani, M. U. Gutierre, A. A. Coppi, R. M. Arida, R. C. Gutierre, Myelin, aging, and physical exercise. *Neurobiol. Aging* **127**, 70–81 (2023).
148. K. A. Phillips, C. M. Watson, A. Bearman, A. R. Knippenberg, J. Adams, C. Ross, S. D. Tardif, Age-related changes in myelin of axons of the corpus callosum and cognitive decline in common marmosets. *Am. J. Primatol.* **81**, e22949 (2019).
149. P. Bonifazi, M. Goldin, M. A. Picardo, I. Jorquera, A. Cattani, G. Bianconi, A. Represa, Y. Ben-Ari, R. Cossart, GABAergic hub neurons orchestrate synchrony in developing hippocampal networks. *Science* **326**, 1419–1424 (2009).
150. C. Lopez-Otin, M. A. Blasco, L. Partridge, M. Serrano, G. Kroemer, Hallmarks of aging: An expanding universe. *Cell* **186**, 243–278 (2023).
151. Y. Liu, L. Sinke, T. H. Jonkman, R. C. Slieker, BIOS Consortium, E. W. van Zwet, L. Daxinger, B. T. Heijmans, The inactive X chromosome accumulates widespread epigenetic variability with age. *Clin. Epigenetics* **15**, 135 (2023).
152. A. Grigoryan, J. Pospiech, S. Kramer, D. Lipka, T. Liehr, H. Geiger, H. Kimura, M. A. Mulaw, M. C. Florian, Attrition of X chromosome inactivation in aged hematopoietic stem cells. *Stem Cell Rep.* **16**, 708–716 (2021).
153. S. Li, J. B. Lund, K. Christensen, J. Baumbach, J. Mengel-From, T. Kruse, W. Li, A. Mohammadnejad, A. Pattie, R. E. Marioni, I. J. Deary, Q. Tan, Exploratory analysis of age and sex dependent DNA methylation patterns on the X-chromosome in whole blood samples. *Genome Med.* **12**, 39 (2020).
154. A. J. Sharp, E. Stathaki, E. Migliavacca, M. Brahmachary, S. B. Montgomery, Y. Dupre, S. E. Antonarakis, DNA methylation profiles of human active and inactive X chromosomes. *Genome Res.* **21**, 1592–1600 (2011).
155. W. Chang, Y. Zhao, D. Rayee, Q. Xie, M. Suzuki, D. Zheng, A. Cvekl, Dynamic changes in whole genome DNA methylation, chromatin and gene expression during mouse lens differentiation. *Epigenetics Chromatin* **16**, 4 (2023).
156. L. Giorgetti, B. R. Lajoie, A. C. Carter, M. Attia, Y. Zhan, J. Xu, C. J. Chen, N. Kaplan, H. Y. Chang, E. Heard, J. Dekker, Structural organization of the inactive X chromosome in the mouse. *Nature* **535**, 575–579 (2016).
157. J. G. Herndon, The grandmother effect: Implications for studies on aging and cognition. *Gerontology* **56**, 73–79 (2010).
158. S. Nattrass, D. P. Croft, S. Ellis, M. A. Cant, M. N. Weiss, B. M. Wright, E. Stredulinsky, T. Doniol-Valcroze, J. K. B. Ford, K. C. Balcomb, D. W. Franks, Postreproductive killer whale grandmothers improve the survival of their grandoffspring. *Proc. Natl. Acad. Sci. U.S.A.* **116**, 26669–26673 (2019).
159. K. Hawkes, Grandmothers and the evolution of human longevity. *Am. J. Hum. Biol.* **15**, 380–400 (2003).
160. K. Hawkes, The grandmother effect. *Nature* **428**, 128–129 (2004).
161. J. S. Peccei, A critique of the grandmother hypotheses: Old and new. *Am. J. Hum. Biol.* **13**, 434–452 (2001).
162. K. A. Wareham, M. F. Lyon, P. H. Glenister, E. D. Williams, Age related reactivation of an X-linked gene. *Nature* **327**, 725–727 (1987).
163. B. P. Balaton, O. Fornes, W. W. Wasserman, C. J. Brown, Cross-species examination of X-chromosome inactivation highlights domains of escape from silencing. *Epigenetics Chromatin* **14**, 12 (2021).
164. H. Fang, C. M. Disteche, J. B. Berletch, X inactivation and escape: Epigenetic and structural features. *Front. Cell Dev. Biol.* **7**, 219 (2019).
165. B. R. Migeon, An overview of X inactivation based on species differences. *Semin. Cell Dev. Biol.* **56**, 111–116 (2016).
166. A. J. Sandweiss, V. L. Brandt, H. Y. Zoghbi, Advances in understanding of Rett syndrome and MECP2 duplication syndrome: Prospects for future therapies. *Lancet Neurol.* **19**, 689–698 (2020).
167. L. M. Lombardi, S. A. Baker, H. Y. Zoghbi, MECP2 disorders: From the clinic to mice and back. *J. Clin. Invest.* **125**, 2914–2923 (2015).
168. N. B. Grimm, J. T. Lee, Selective Xi reactivation and alternative methods to restore MECP2 function in Rett syndrome. *Trends Genet.* **38**, 920–943 (2022).
169. A. Zito, J. T. Lee, Variable expression of MECP2, CDKL5, and FMR1 in the human brain: Implications for gene restorative therapies. *Proc. Natl. Acad. Sci. U.S.A.* **121**, e2312757121 (2024).
170. R. A. Neff, M. Wang, S. Vatansever, L. Guo, C. Ming, Q. Wang, E. Wang, E. Horgusluoglu-Moloch, W. M. Song, A. Li, E. L. Castranio, J. Tcw, L. Ho, A. Goate, V. Fossati, S. Noggle, S. Gandy, M. E. Ehrlich, P. Katsel, E. Schadt, D. Cai, K. J. Brennand, V. Haroutunian, B. Zhang, Molecular subtyping of Alzheimer's disease using RNA sequencing data reveals novel mechanisms and targets. *Sci. Adv.* **7**, eabb5398 (2021).
171. C. Xu, M. Prete, S. Webb, L. Jardine, B. J. Stewart, R. Hoo, P. He, K. B. Meyer, S. A. Teichmann, Automatic cell-type harmonization and integration across Human Cell Atlas datasets. *Cell* **186**, 5876–5891.e20 (2023).
172. C. Dominguez Conde, C. Xu, L. B. Jarvis, D. B. Rainbow, S. B. Wells, T. Gomes, S. K. Howlett, O. Suchanek, K. Polanski, H. W. King, L. Mamanova, N. Huang, P. A. Szabo, L. Richardson, L. Bolt, E. S. Fasouli, K. T. Mahbubani, M. Prete, L. Tuck, N. Richoz, Z. K. Tuong, L. Campos, H. S. Mousa, E. J. Needham, S. Pritchard, T. Li, R. Elmentaite, J. Park, E. Rahmani, D. Chen, D. K. Menon, O. A. Bayraktar, L. K. James, K. B. Meyer, N. Yosef, M. R. Clatworthy, P. A. Sims, D. L. Farber, K. Saeb-Parsy, J. L. Jones, S. A. Teichmann, Cross-tissue immune cell analysis reveals tissue-specific features in humans. *Science* **376**, eabl5197 (2022).
173. E. Caglayan, Y. Liu, G. Konopka, Neuronal ambient RNA contamination causes misinterpreted and masked cell types in brain single-nuclei datasets. *Neuron* **110**, 4043–4056.e5 (2022).
174. S. L. Wolock, R. Lopez, A. M. Klein, Scrublet: Computational identification of cell doublets in single-cell transcriptomic data. *Cell Syst.* **8**, 281–291.e9 (2019).
175. S. Abdulai-Saiku, S. Gupta, D. Wang, F. Marino, A. J. Moreno, Y. Huang, D. Srivastava, B. Panning, D. B. Dubal, The maternal X chromosome affects cognition and brain ageing in female mice. *Nature* **638**, 152–159 (2025).
176. A. Ferraj, P. A. Audano, P. Balachandran, A. Czechanski, J. I. Flores, A. A. Radecki, V. Mosur, D. S. Gordon, I. A. Walawalkar, E. E. Eichler, L. G. Reinholdt, C. R. Beck, Resolution of structural variation in diverse mouse genomes reveals chromatin remodeling due to transposable elements. *Cell Genom.* **3**, 100291 (2023).
177. J. F. Degner, J. C. Marioni, A. A. Pai, J. K. Pickrell, E. Nkadori, Y. Gilad, J. K. Pritchard, Effect of read-mapping biases on detecting allele-specific expression from RNA-sequencing data. *Bioinformatics* **25**, 3207–3212 (2009).
178. M. Lewandoski, K. M. Wassarman, G. R. Martin, Zp3-cre, a transgenic mouse line for the activation or inactivation of loxP-flanked target genes specifically in the female germ line. *Curr. Biol.* **7**, 148–151 (1997).

179. U. Raudvere, L. Kolberg, I. Kuzmin, T. Arak, P. Adler, H. Peterson, J. Vilo, g:Profiler: A web server for functional enrichment analysis and conversions of gene lists (2019 update). *Nucleic Acids Res.* **47**, W191–W198 (2019).
180. M. Wang, A. Li, M. Sekiya, N. D. Beckmann, X. Quan, N. Schrode, M. B. Fernando, A. Yu, L. Zhu, J. Cao, L. Lyu, E. Horgusluoglu, Q. Wang, L. Guo, Y.-s. Wang, R. Neff, W.-m. Song, E. Wang, Q. Shen, X. Zhou, C. Ming, S.-M. Ho, S. Vatansever, H. Ü. Kaniskan, J. Jin, M.-M. Zhou, K. Ando, L. Ho, P. A. Slesinger, Z. Yue, J. Zhu, P. Katsel, S. Gandy, M. E. Ehrlich, V. Fossati, S. Noggle, D. Cai, V. Haroutunian, K. M. Iijima, E. Schadt, K. J. Brennand, B. Zhang, Transformative network modeling of multi-omics data reveals detailed circuits, key regulators, and potential therapeutics for Alzheimer's disease. *Neuron* **109**, 257–272.e14 (2021).
181. M. Wang, N. D. Beckmann, P. Roussos, E. Wang, X. Zhou, Q. Wang, C. Ming, R. Neff, W. Ma, J. F. Fullard, M. E. Hauberg, J. Bendl, M. A. Peters, B. Logsdon, P. Wang, M. Mahajan, L. M. Mangravite, E. B. Dammer, D. M. Duong, J. J. Lah, N. T. Seyfried, A. I. Levey, J. D. Buxbaum, M. Ehrlich, S. Gandy, P. Katsel, V. Haroutunian, E. Schadt, B. Zhang, The Mount Sinai cohort of large-scale genomic, transcriptomic and proteomic data in Alzheimer's disease. *Sci. Data* **5**, 180185 (2018).
182. M. I. Love, W. Huber, S. Anders, Moderated estimation of fold change and dispersion for RNA-seq data with DESeq2. *Genome Biol.* **15**, 550 (2014).
183. A. D. Edelstein, M. A. Tsuchida, N. Amodaj, H. Pinkard, R. D. Vale, N. Stuurman, Advanced methods of microscope control using μManager software. *J. Biol. Methods* **1**, e10 (2014).
184. J. Schindelin, I. Arganda-Carreras, E. Frise, V. Kaynig, M. Longair, T. Pietzsch, S. Preibisch, C. Rueden, S. Saalfeld, B. Schmid, J. Y. Tinevez, D. J. White, V. Hartenstein, K. Eliceiri, P. Tomancak, A. Cardona, Fiji: An open-source platform for biological-image analysis. *Nat. Methods* **9**, 676–682 (2012).
185. S. Schildge, C. Bohrer, K. Beck, C. Schachtrup, Isolation and culture of mouse cortical astrocytes. *J. Vis. Exp.* , 50079 (2013).
186. J. Schwieger, K. H. Esser, T. Lenarz, V. Scheper, Establishment of a long-term spiral ganglion neuron culture with reduced glial cell number: Effects of AraC on cell composition and neurons. *J. Neurosci. Methods* **268**, 106–116 (2016).
187. D. B. Dubal, L. Zhu, P. E. Sanchez, K. Worden, L. Broestl, E. Johnson, K. Ho, G. Q. Yu, D. Kim, A. Betourne, O. M. Kuro, E. Masliah, C. R. Abraham, L. Mucke, Life extension factor klotho prevents mortality and enhances cognition in hAPP transgenic mice. *J. Neurosci.* **35**, 2358–2371 (2015).
188. C. Park, O. Hahn, S. Gupta, A. J. Moreno, F. Marino, B. Kedir, D. Wang, S. A. Villeda, T. Wyss-Coray, D. B. Dubal, Platelet factors are induced by longevity factor klotho and enhance cognition in young and aging mice. *Nat. Aging* **3**, 1067–1078 (2023).
189. D. B. Dubal, J. S. Yokoyama, L. Zhu, L. Broestl, K. Worden, D. Wang, V. E. Sturm, D. Kim, E. Klein, G. Q. Yu, K. Ho, K. E. Eilertson, L. Yu, M. Kuro-o, P. L. De Jager, G. Coppola, G. W. Small, D. A. Bennett, J. H. Kramer, C. R. Abraham, B. L. Miller, L. Mucke, Life extension factor klotho enhances cognition. *Cell Rep.* **7**, 1065–1076 (2014).
190. J. Leon, A. J. Moreno, B. I. Garay, R. J. Chalkley, A. L. Burlingame, D. Wang, D. B. Dubal, Peripheral elevation of a klotho fragment enhances brain function and resilience in young, aging, and α-synuclein transgenic mice. *Cell Rep.* **20**, 1360–1371 (2017).
191. F. Dellu, W. Mayo, J. Cherkaoui, M. Le Moal, H. Simon, A two-trial memory task with automated recording: Study in young and aged rats. *Brain Res.* **588**, 132–139 (1992).
192. L. A. Goff, A. F. Groff, M. Sauvageau, Z. Trayes-Gibson, D. B. Sanchez-Gomez, M. Morse, R. D. Martin, L. E. Elcavage, S. C. Liapis, M. Gonzalez-Celeiro, O. Plana, E. Li, C. Gerhardinger, G. S. Tomassy, P. Arlotta, J. L. Rinn, Spatiotemporal expression and transcriptional perturbations by long noncoding RNAs in the mouse brain. *Proc. Natl. Acad. Sci. U.S.A.* **112**, 6855–6862 (2015).
193. G. Figlia, S. Muller, A. M. Hagenston, S. Kleber, M. Roiuk, J. P. Quast, N. Ten Bosch, D. Carvajal Ibanez, D. Mauceri, A. Martin-Villalba, A. A. Teleman, Brain-enriched RagB isoforms regulate the dynamics of mTORC1 activity through GATOR1 inhibition. *Nat. Cell Biol.* **24**, 1407–1421 (2022).
194. S. H. Jung, M. L. Brownlow, M. Pellegrini, R. Jankord, Divergence in Morris water maze-based cognitive performance under chronic stress is associated with the hippocampal whole transcriptomic modification in mice. *Front. Mol. Neurosci.* **10**, 275 (2017).
195. C. Montani, M. Ramos-Brossier, L. Ponzoni, L. Gritti, A. W. Cwetsch, D. Braida, Y. Saillour, B. Terragni, M. Mantegazza, M. Sala, C. Verpelli, P. Billuart, C. Sala, The X-linked intellectual disability protein IL1RAPL1 regulates dendrite complexity. *J. Neurosci.* **37**, 6606–6627 (2017).
196. C. Montani, L. Gritti, S. Beretta, C. Verpelli, C. Sala, The synaptic and neuronal functions of the X-linked intellectual disability protein interleukin-1 receptor accessory protein like 1 (IL1RAPL1). *Dev. Neurobiol.* **79**, 85–95 (2019).
197. D. Rujescu, E. M. Meisenzahl, S. Krejcova, I. Giegling, T. Zetzsche, M. Reiser, C. M. Born, H.-J. Möller, A. Veske, A. Gal, U. Finckh, Plexin B3 is genetically associated with verbal performance and white matter volume in human brain. *Mol. Psychiatry* **12**, 190–194 (2007).

Acknowledgments: We acknowledge D. Velasco for illustrations in Figs. 1A, 4A, and 7A; E. Kakoshina for formatting Fig. 2; A. Moreno for illustrations in Figs. 6D and 7A; C. Chen and E. Davis for mouse colony management; S. Nanda for helping with the initial round of genomic library processing; and the Center for Advanced Light Microscopy for assistance with imaging. **Funding:** This work was supported by NIH grants RF1AG079176-01 and RF1AG068325-01 (to D.B.D.), Simons Foundation grant 811225SPI (to D.B.D.), Simons Foundation Transition to Independence Award (to S.A.-S.), Philanthropy (to D.B.D.), Bakar Aging Research Institute (to F.M. and D.B.D.), Simons Foundation grant SF811217 (to B.A.B.), NIH-NIBIB T32 EB001631 (to L.W.B.), RSNA R&E Foundation RR24-217 (to L.W.B.), NIH-NIA R01AG062588 (to J.S.Y.), Alzheimer's Association AARF-23-1145318 (to R.S.), and New Vision Research CCAD 2024-001-1 (to R.S.). **Author contributions:** Conceptualization: M.G., C.K.S., V.R., and D.B.D. Methodology: M.G., C.K.S., S.A.-S., R.S., L.W.B., B.A.B., K.B.C., V.R., and D.B.D. Investigation: M.G., C.K.S., S.A.-S., R.S., F.M., D.W., and L.W.B. Visualization: M.G., C.K.S., S.A.-S., R.S., F.M., and D.B.D. Supervision: J.S.Y., B.P., B.A.B., K.B.C., V.R., and D.B.D. Writing—original draft: M.G. and D.B.D. Writing—review and editing: M.G., C.K.S., S.A.-S., R.S., F.M., D.W., L.W.B., J.S.Y., B.P., B.A.B., K.B.C., V.R., and D.B.D. **Competing interests:** D.B.D. serves on the Board of the Glenn Medical Foundation and consulted for Unity Biotechnology (unrelated to the content of the manuscript) and SV Health Investors (unrelated to the content of the manuscript). J.S.Y. serves on the scientific advisory board for the Epstein Family Alzheimer's Research Collaboration. All other authors declare that they have no competing interests. **Data and materials availability:** All data are available in the main text or Supplementary Materials or as raw and processed files available at GEO series accession number GSE276652. Codes for the sequencing filtering normalization, Xa/Xi expression, and pseudobulking are available at https://github.com/margaretgadek/XCIEscapeAging-/releases/tag/v1.0.0 and https://zenodo.org/records/13750335.

Submitted 11 September 2024
Accepted 31 December 2024
Published 5 March 2025
10.1126/sciadv.ads8169
