# Supplementary material for: Aging activates escape of the silent X chromosome in the female mouse hippocampus
Source: Sci Adv. 2025 Mar 5;11(10):eads8169. doi: 10.1126/sciadv.ads8169 (PMC11881916; doi:10.1126/sciadv.ads8169)
Supplement: Supplementary file 2 — Supplementary Text Figs. S1 to S5 Table S1 Legends for data S1 to S8 References [file sciadv.ads8169_sm.pdf]

Science Advances
AAAS

Supplementary Materials for

Aging activates escape of the silent X chromosome in the female mouse hippocampus

Margaret Gadek *et al.*

Corresponding author: Dena B. Dubal, dena.dubal@ucsf.edu

Sci. Adv. **11**, eads8169 (2025)
DOI: 10.1126/sciadv.ads8169

The PDF file includes:

Supplementary Text
Figs. S1 to S5
Table S1
Legends for data S1 to S8
References

Other Supplementary Material for this manuscript includes the following:

Data S1 to S8

Supplementary Text

Enrichment of differentially expressed genes on the X chromosome in the aging hippocampus

The most frequent neuronal DEGs were, *Mamld1, Kantr*, and *Rragb*, expressed from the Xa, and *Ftx*, expressed from the Xi. Each gene was upregulated in at least 4/5 cell types (**Fig. 2 F-J**). *Kantr* and *Rragb* have established roles in the neurons, in axon guidance(*192*) and mTORC1 signaling(*193*), respectively; thus, these functions may be modulated in hippocampal neuron aging. *Ftx* is discussed at length in the main text.

In multiple glial populations, *Gm14634*, *Frmpd4*, *Il1rapl1, Plxnb3,* from the Xa and *Gpm6b* and *Plp1* from the Xi were upregulated (**Fig 2**. **H-N**). *Gm14634* has been associated with Morris water maze performance, which assesses spatial memory(*194*); therefore, it may be modulated as memory declines with aging. *Frmpd4* (*67*) and *Il1rapl1*(*195, 196*) are associated with intellectual disability and specific haplotypes of *Plxnb3* are associated with cognitive performance (*197*). Thus, changes in the glial enriched genes could modulate cognition with aging. *Gpm6b* and *Plp1* are discussed at length in the main text.

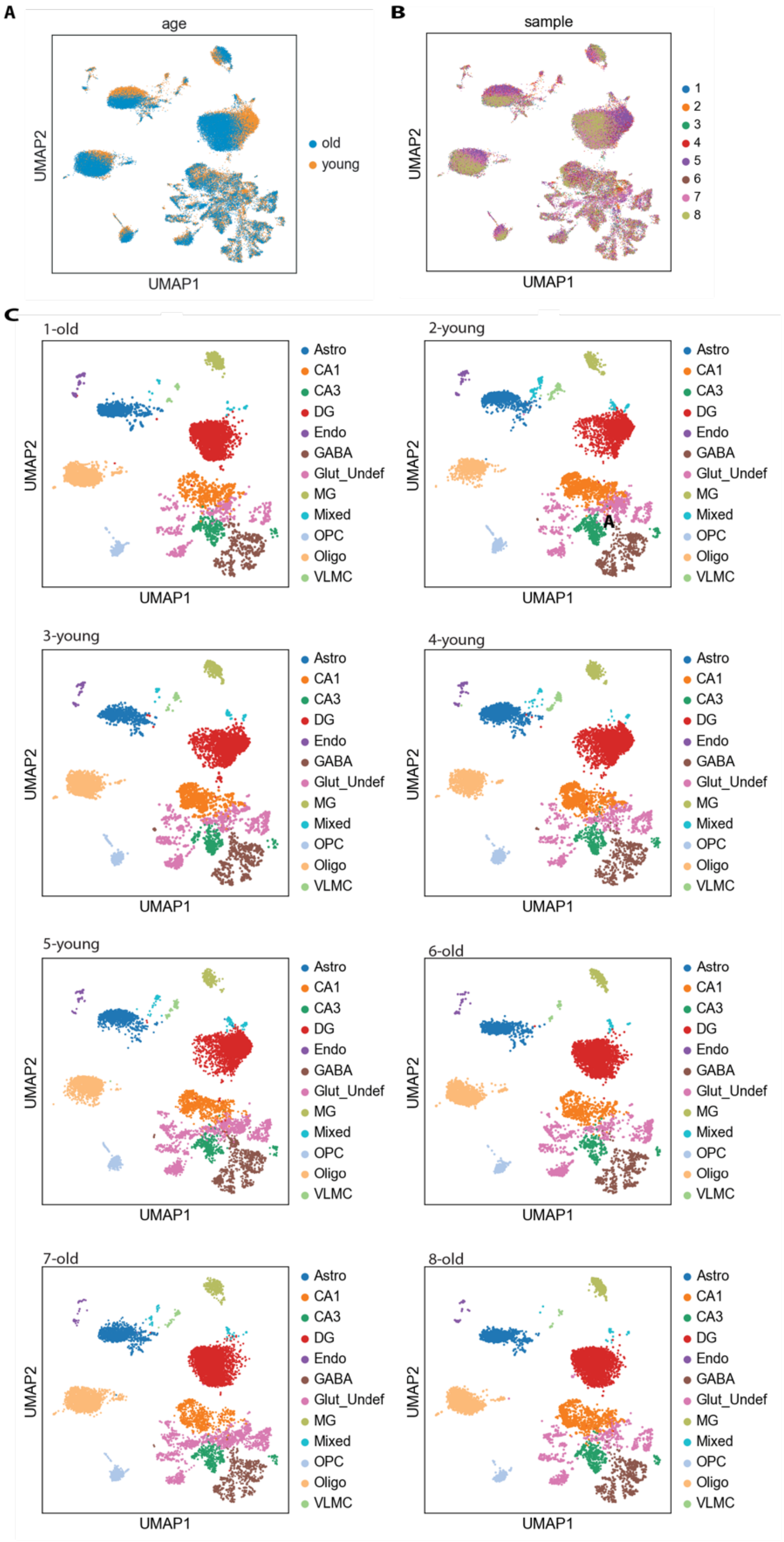

Fig. S1. Young and old nuclei across cell types

A. Uniform manifold approximation and projection (UMAP) showing the distribution of young and old nuclei across cell types.

B. UMAP showing distribution of samples
C. UMAP showing cell type distribution per sample

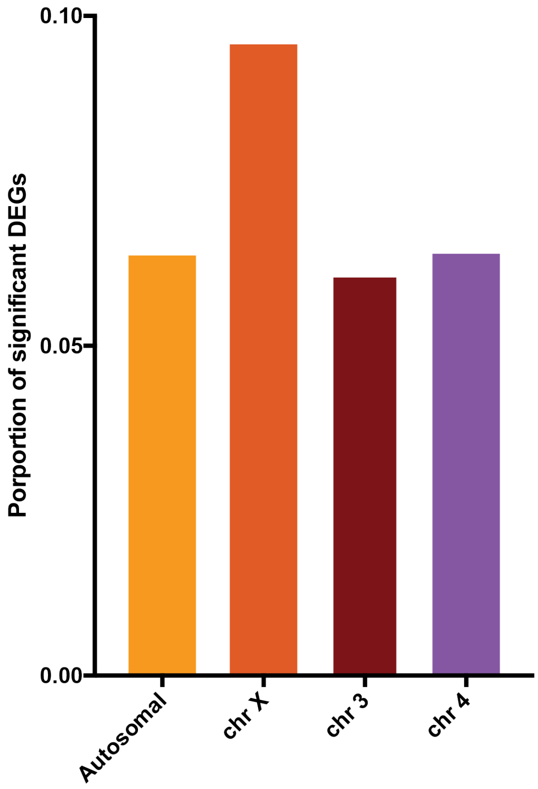

Fig. S2. Enrichment of X chromosome differentially expressed genes (DEGs)
Significant (adjusted pval <0.05 and log_2fold change <-0.1 or log_2fold change > 0.1) DEGs from the autosome and X chromosomes (relevant to Figure 2B), plus chromosomes 3 and 4, normalized to total expressed genes. Chromosomes 3 (1670 *M. musculus* genes) and 4 (1894 *M. musculus* genes) contain a similar amount of genes as the X chromosome (1537 *M. musculus* genes).

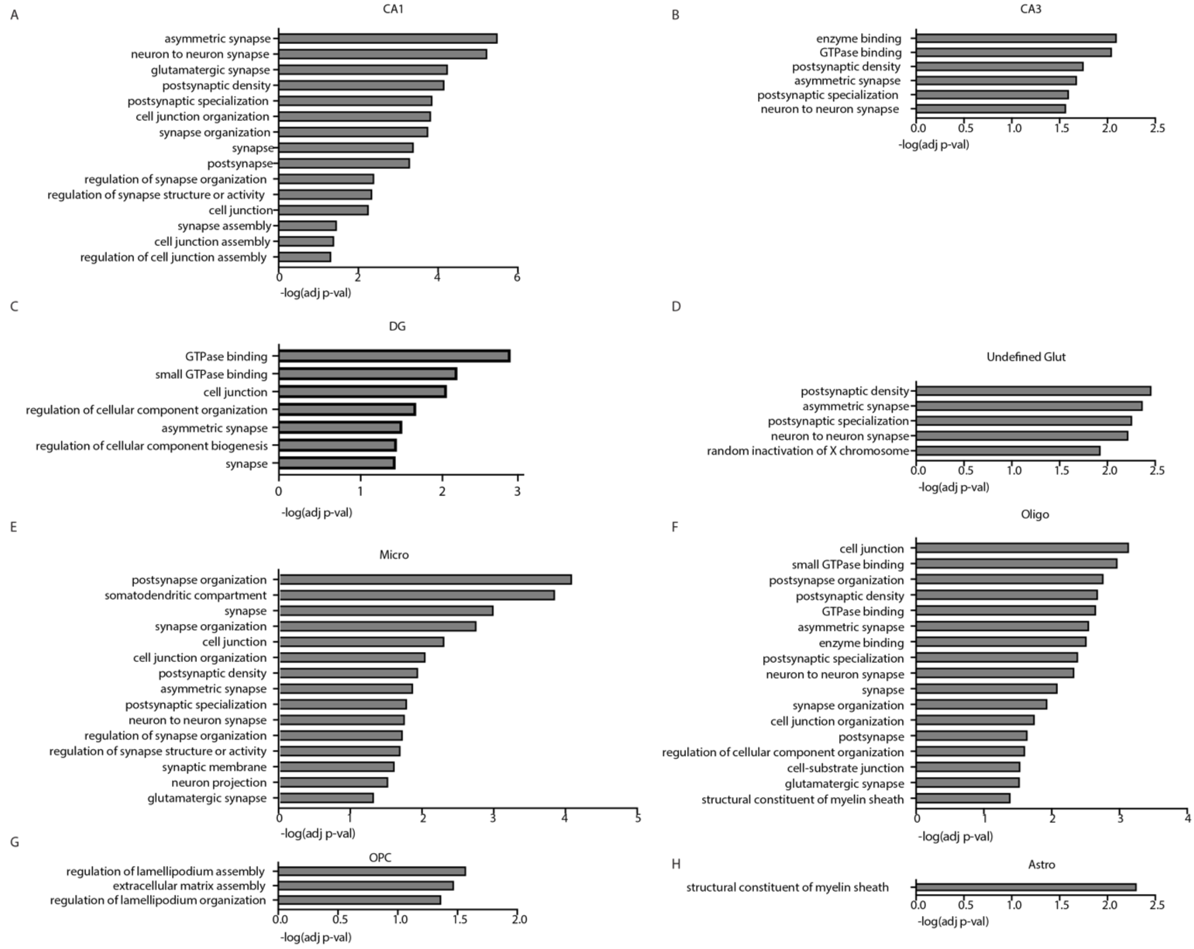

Fig. S3. Gene ontology (GO) term analysis of DEGs by cell types

A. GO analysis of X chromosome DEGs in CA1 neurons represented by -log(adjusted p-value).
B. GO analysis of X chromosome DEGs in CA3 neurons represented by -log(adjusted p-value).
C. GO analysis of X chromosome DEGs in DG neurons represented by -log(adjusted p-value).
D. GO analysis of X chromosome DEGs in undefined glutamatergic neurons represented by -log(adjusted p-value).
E. GO analysis of X chromosome DEGs in microglia represented by -log(adjusted p-value).
F. GO analysis of X chromosome DEGs in oligodendrocytes represented by -log(adjusted p-value).
G. GO analysis of X chromosome DEGs in oligodendrocyte progenitor cells (OPCs) represented by -log(adjusted p-value).
H. GO analysis of X chromosome DEGs in astrocytes progenitor cells (OPCs) represented by -log(adjusted p-value).

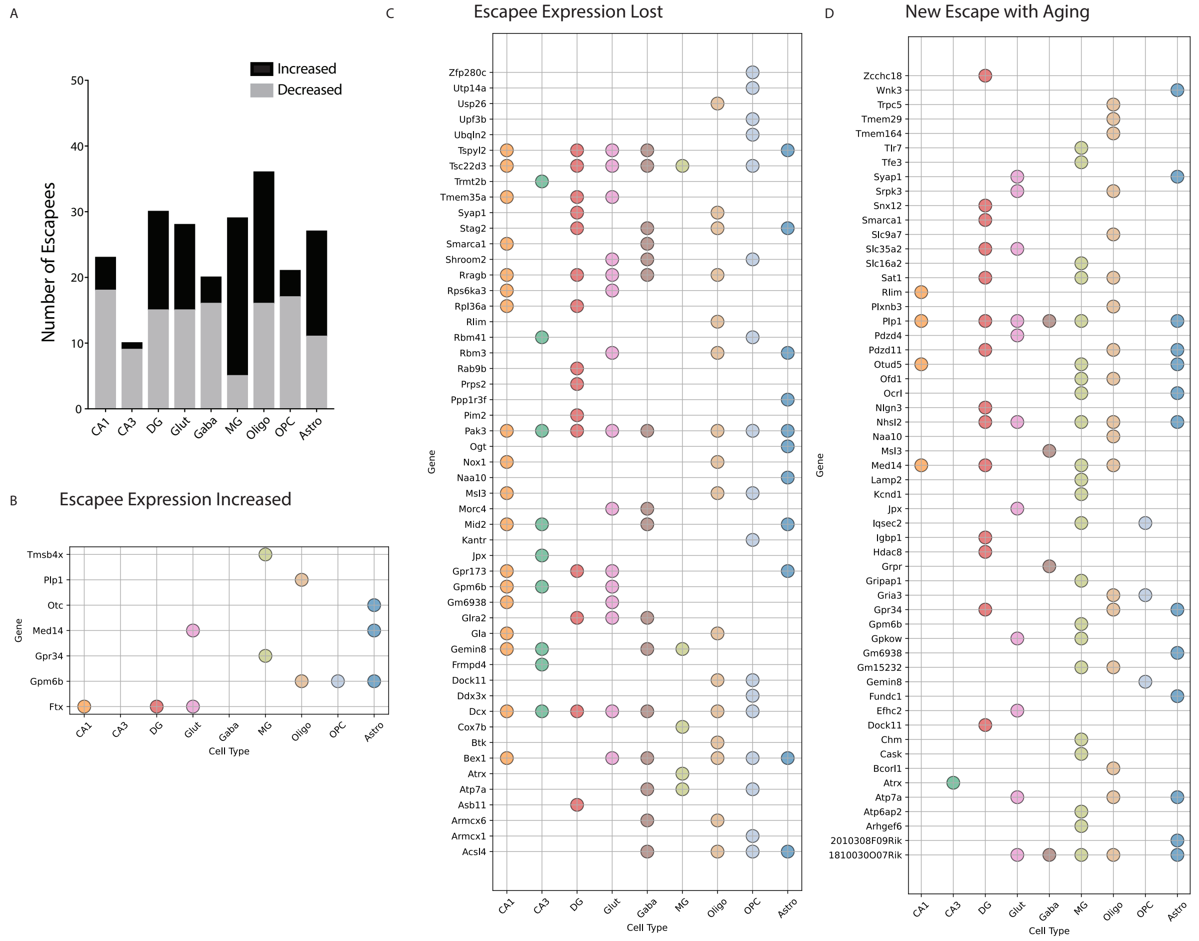

Fig. S4. Patterns of escape of the X chromosome with age

A. Number of escapees that were increased (escapee expression increased and new escape with aging) and decreased (escapee expression lost) with age by cell type.

B. Escapee expression increased by cell type, indicated by the presence of a circle at the intersection of the corresponding gene and cell type

C. Escapee expression lost with age by cell type, indicated by the presence of a circle at the intersection of the corresponding gene and cell type

D. New escape with aging by cell type, indicated by the presence of a circle at the intersection of the corresponding gene and cell type

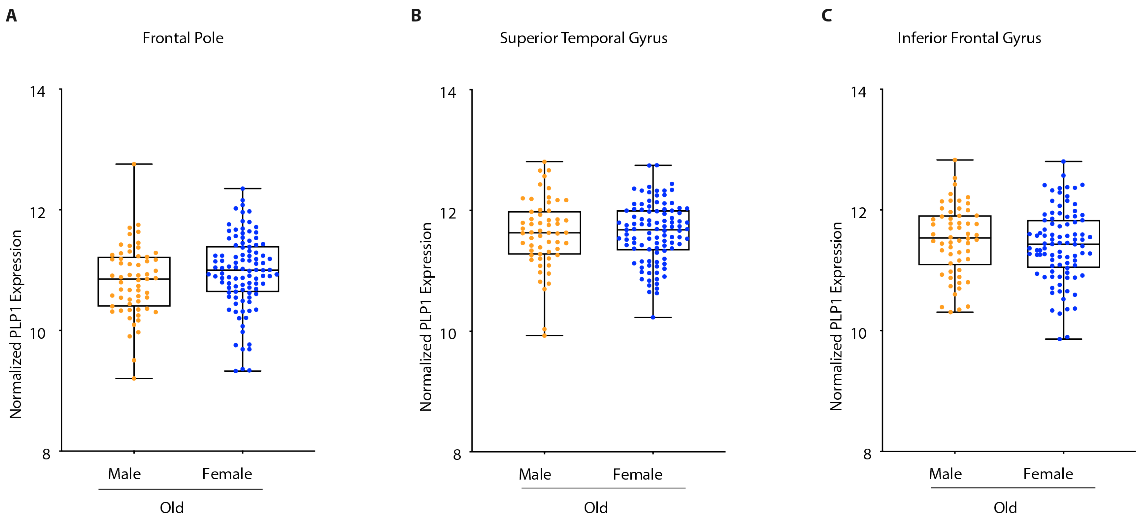

Fig. S5. Human Plp1 expression by brain region and sex

A. Relative *PLP1* mRNA expression in the frontal pole from postmortem tissue of old men and women. Depicted *PLP1* data underwent normalization, log2-transformation, and residualization for covariates. Box plots represent the median and 25th and 75th percentiles, and box hinges represent the interquartile range of the two middle quartiles within a group. Min and max data points define the extent of whiskers (error bars). (XY=59, XX=102)

B. Relative *PLP1* mRNA expression in the superior temporal gyrus from postmortem tissue of old men and women. (XY=58, XX=101)

C. Relative *PLP1* mRNA expression in the inferior frontal gyrus from postmortem tissue of old men and women. (XY=58, XX=92)

Genes	ID	DEG	Esc	Ref
1810030O07Rik			x	
2010308F09Rik			x	
Acsl4	x		x	85
Arhgef6	x		x	68
Armcx1			x	
Armcx6			x	
Asb11			x	
Atp6ap2	x		x	86
Atp7a	x		x	69
Atrx	x		x	87
Bcorl1	x		x	88
Bex1			x	
Btk	x		x	89
Cask	x	x	x	70
Chm	x		x	90
Cox7b	x		x	71
Dcx	x		x	72
Ddx3x	x		x	91
Diaph2		x		
Dock11			x	
Efhc2	x		x	73
Frmpd4	x		x	67
Ftx	x	x	x	74
Fundc1			x	
Gemin8			x	
Gla			x	
Glra2	x		x	92
Gm15232			x	
Gm6938			x	
Gpkow			x	
Gpm6b		x	x	
Gpr173			x	
Gpr34		x	x	
Gria3	x		x	75
Gripap1	x		x	93
Grpr			x	
Hdac8	x	x	x	76
Igbp1			x	
Iqsec2	x		x	94
Jpx	x	x	x	74
Kantr		x	x	
Kcnd1			x	
Lamp2	x		x	95
Med14			x	
Mid2	x		x	77
Morc4			x	
Msl3	x		x	96
Naa10	x		x	97
Nhsl2		x	x	
Nlgn3	x		x	98
Nox1			x	

Genes	ID	DEG	Esc	Ref
Ocrl	x		x	78
Ofd1	x		x	114
Ogt	x		x	79
Otc	x		x	99
Otud5	x		x	100
Pak3	x		x	80
Pdzd11			x	
Pdzd4			x	
Pim2			x	
Plp1	x	x	x	115
Plxnb3		x	x	
Ppp1r3f	x		x	101
Prps2			x	
Rab9b			x	
Rbm3			x	
Rbm41			x	
Rlim	x		x	102
Rpl36a			x	
Rps6ka3	x	x	x	82
Rragb			x	
Sat1			x	
Shroom2			x	
Slc16a2	x		x	103
Slc35a2	x		x	104
Slc9a7	x		x	105
Smarca1	x		x	106
Snx12			x	
Srpk3	x		x	107
Stag2	x		x	108
Syap1			x	
Tfe3	x		x	109
Tlr7			x	
Tmem164	x		x	83
Tmem29			x	
Tmem35a			x	
Tmsb4x			x	
Trmt2b			x	
Trpc5	x		x	110
Tsc22d3		x	x	
Tspan7	x	x		84
Tspyl2	x		x	111
Ubqln2			x	
Upf3b	x		x	112
Usp26			x	
Utp14a			x	
Wnk3	x		x	113
Xist		x		
Zcchc18			x	
Zfp280c			x	

Table S1. DEGs and escapees in aging and human intellectual disability Cell-type specific Xi DEGs (**Fig. 2**) and genes following one of three escapee expression patterns with aging (**Figs. 3, S4, 4**) (increased escapee expression, loss of escapee expression, and new escape) and their presence in human intellectual disability.

Data S1. (separate file)
Quality control metrics by sample, provided by 10X Genomics Cell Ranger. Columns show sample, age, estimated number of cells, mean reads per cell, Median UMI Counts per nucleus (M. *musculus*), Median UMI Counts per nucleus (*M. castaneus*), Median Genes per nucleus (*M. musculus*), and Median Genes per nucleus (*M. castaneus*).

Data S2. (separate file)
Top 100 genes ranked in each cell type cluster. Genes are ranked by the Scanpy ranked_genes_groups function using the Wilcox rank-sum method. Clusters are made by the Leiden algorithm at a 0.5 resolution.

Data S3. (separate file)
Cell type composition by sample. Sample name and age group is noted followed by the number of nuclei aligning with each cell type group.

Data S4. (separate file)
DEGs for bulk and cell type-specific populations. DESeq2 statistics including baseMean, log2FoldChange, log fold change standard error (lfcSE), pvalue, and adjusted pvalue (padj) are included. Statistically significant genes are denoted by "s" in the sig column. Cell types are noted in the X column. The presence of the gene on the X, 3, and 4 chromosomes is noted in the xchr, 3chr, and 4chr columns by "X," "3", or "4" in the corresponding columns.

Data S5. (separate file)
Significant DEGs (adjusted pval <0.05 and $\log_2$fold change <-0.1 or $\log_2$fold change > 0.1) from the combined "All" cell types group were found in other publications profiling the aging brain, following a similar format to S2. Direction of Gadek et al. DEGs, followed by columns comparing Gadek et al. direction to bulk DEGs from the Ximerakis et al. (*37*) and each hippocampal cell types from Hajdarovic et al.(32). The comparison columns have a 1 if the DEGs match in the same direction, 0 if they conflict, and N/A if not found. Sum of comparisons DEGs are reported in the final column.

Data S6. (separate file)
Escape calculation spreadsheets (Escape_Calc_CellType) containing escape calculations and confidence intervals by cell types. Escape median and 99% confidence interval calculations and adjustments, and corresponding categorization in old and young cell types are shown. Escape categorization by cell type spreadsheets (CellType Summary). The table includes genes by category and the number of genes in each category.

Data S7. (separate file)
Cell type comparison between manual annotation of cell types and automated annotation by CellTypist. Manual and CellTypist's predicted labels and majority voting are noted for each barcode. A simplified classification to compare the broad manual cell types and CellTypist cell types is also included. Cell type classes of each category (neuron, glia, other) are also compared.

Data S8. (separate file)
Data depicted in each figure with small n. Figure number is referenced in the spreadsheet tab.

REFERENCES AND NOTES

1. D. B. Dubal, C. Murphy, Y. Suh, B. A. Benayoun, Biological sex matters in brain aging. *Neuron* **113**, 2–6 (2025).

2. V. Zarulli, J. A. Barthold Jones, A. Oksuzyan, R. Lindahl-Jacobsen, K. Christensen, J. W. Vaupel, Women live longer than men even during severe famines and epidemics. *Proc. Natl. Acad. Sci. U.S.A.* **115**, E832–E840 (2018).

3. United Nations Department of Economic and Social Affairs, Population Division, *World Population Ageing* (United Nations Department of Economic and Social Affairs, 2020).

4. A. M. Bronikowski, R. P. Meisel, P. R. Biga, J. R. Walters, J. E. Mank, E. Larschan, G. S. Wilkinson, N. Valenzuela, A. M. Conard, J. P. de Magalhaes, J. E. Duan, A. E. Elias, T. Gamble, R. M. Graze, K. E. Gribble, J. A. Kreiling, N. C. Riddle, Sex-specific aging in animals: Perspective and future directions. *Aging Cell* **21**, e13542 (2022).

5. E. L. Barrett, D. S. Richardson, Sex differences in telomeres and lifespan. *Aging Cell* **10**, 913–921 (2011).

6. A. M. Bronikowski, J. Altmann, D. K. Brockman, M. Cords, L. M. Fedigan, A. Pusey, T. Stoinski, W. F. Morris, K. B. Strier, S. C. Alberts, Aging in the natural world: Comparative data reveal similar mortality patterns across primates. *Science* **331**, 1325–1328 (2011).

7. T. H. Clutton-Brock, K. Isvaran, Sex differences in ageing in natural populations of vertebrates. *Proc. Biol. Sci.* **274**, 3097–3104 (2007).

8. M. Bou Sleiman, S. Roy, A. W. Gao, M. C. Sadler, G. V. G. von Alvensleben, H. Li, S. Sen, D. E. Harrison, J. F. Nelson, R. Strong, R. A. Miller, Z. Kutalik, R. W. Williams, J. Auwerx, Sex- and age-dependent genetics of longevity in a heterogeneous mouse population. *Science* **377**, eabo3191 (2022).

9. C. J. Cheng, J. A. L. Gelfond, R. Strong, J. F. Nelson, Genetically heterogeneous mice exhibit a female survival advantage that is age- and site-specific: Results from a large multi-site study. *Aging Cell* **18**, e12905 (2019).

10. E. J. Davis, I. Lobach, D. B. Dubal, Female XX sex chromosomes increase survival and extend lifespan in aging mice. *Aging Cell* **18**, e12871 (2019).

11. S. N. Austad, K. E. Fischer, Sex differences in lifespan. *Cell Metab.* **23**, 1022–1033 (2016).

12. E. M. Crimmins, J. K. Kim, A. Sole-Auro, Gender differences in health: Results from SHARE, ELSA and HRS. *Eur. J. Public Health* **21**, 81–91 (2011).

13. E. H. Gordon, R. E. Hubbard, Do sex differences in chronic disease underpin the sex-frailty paradox? *Mech. Ageing Dev.* **179**, 44–50 (2019).

14. S. Horvath, M. Gurven, M. E. Levine, B. C. Trumble, H. Kaplan, H. Allayee, B. R. Ritz, B. Chen, A. T. Lu, T. M. Rickabaugh, B. D. Jamieson, D. Sun, S. Li, W. Chen, L. Quintana-Murci, M. Fagny, M. S. Kobor, P. S. Tsao, A. P. Reiner, K. L. Edlefsen, D. Absher, T. L. Assimes, An epigenetic clock analysis of race/ethnicity, sex, and coronary heart disease. *Genome Biol.* **17**, 171 (2016).

15. M. S. Goyal, T. M. Blazey, Y. Su, L. E. Couture, T. J. Durbin, R. J. Bateman, T. L. Benzinger, J. C. Morris, M. E. Raichle, A. G. Vlassenko, Persistent metabolic youth in the aging female brain. *Proc. Natl. Acad. Sci. U.S.A.* **116**, 3251–3255 (2019).

16. E. M. Arenaza-Urquijo, R. Boyle, K. Casaletto, K. J. Anstey, C. Vila-Castelar, A. Colverson, E. Palpatzis, J. M. Eissman, T. Kheng Siang Ng, S. Raghavan, M. Akinci, J. M. J. Vonk, L. S. Machado, P. P. Zanwar, H. L. Shrestha, M. Wagner, S. Tamburin, H. R. Sohrabi, S. Loi, D. Bartres-Faz, D. B. Dubal, P. Vemuri, O. Okonkwo, T. J. Hohman, M. Ewers, R. F. Buckley, Reserve, Resilience and Protective Factors Professional Interest Area, Sex and Gender Professional Interest area and the ADDRESS! Special Interest Group, Sex and gender differences in cognitive resilience to aging and Alzheimer's disease. *Alzheimers Dement.* **20**, 5695–5719 (2024).

17. K. B. Casaletto, F. M. Elahi, A. M. Staffaroni, S. Walters, W. R. Contreras, A. Wolf, D. Dubal, B. Miller, K. Yaffe, J. H. Kramer, Cognitive aging is not created equally: Differentiating unique cognitive phenotypes in "normal" adults. *Neurobiol. Aging* **77**, 13–19 (2019).

18. C. R. Jack, Jr., H. J. Wiste, S. D. Weigand, D. S. Knopman, P. Vemuri, M. M. Mielke, V. Lowe, M. L. Senjem, J. L. Gunter, M. M. Machulda, B. E. Gregg, V. S. Pankratz, W. A. Rocca, R. C. Petersen, Age,

sex, and APOE ε4 effects on memory, brain structure, and β-amyloid across the adult life span. *JAMA Neurol.* **72**, 511–519 (2015).

19. D. B. Dubal, Sex difference in Alzheimer's disease: An updated, balanced and emerging perspective on differing vulnerabilities. *Handb. Clin. Neurol.* **175**, 261–273 (2020).

20. A. C. McCarrey, Y. An, M. H. Kitner-Triolo, L. Ferrucci, S. M. Resnick, Sex differences in cognitive trajectories in clinically normal older adults. *Psychol. Aging* **31**, 166–175 (2016).

21. D. A. Levine, A. L. Gross, E. M. Briceno, N. Tilton, B. J. Giordani, J. B. Sussman, R. A. Hayward, J. F. Burke, S. Hingtgen, M. S. V. Elkind, J. J. Manly, R. F. Gottesman, D. J. Gaskin, S. Sidney, R. L. Sacco, S. E. Tom, C. B. Wright, K. Yaffe, A. T. Galecki, Sex differences in cognitive decline among US adults. *JAMA Netw. Open* **4**, e210169 (2021).

22. F. Marino, D. Wang, G. E. Merrihew, M. J. MacCoss, D. B. Dubal, A second X chromosome improves cognition in aging male and female mice. bioRxiv 2024.07.26.605328 [Preprint] (2024). https://doi.org/10.1101/2024.07.26.605328.

23. E. J. Davis, L. Broestl, S. Abdulai-Saiku, K. Worden, L. W. Bonham, E. Minones-Moyano, A. J. Moreno, D. Wang, K. Chang, G. Williams, B. I. Garay, I. Lobach, N. Devidze, D. Kim, C. Anderson-Bergman, G. Q. Yu, C. C. White, J. A. Harris, B. L. Miller, D. A. Bennett, A. P. Arnold, P. L. De Jager, J. J. Palop, B. Panning, J. S. Yokoyama, L. Mucke, D. B. Dubal, A second X chromosome contributes to resilience in a mouse model of Alzheimer's disease. *Sci. Transl. Med.* **12**, eaaz5677 (2020).

24. A. Loda, S. Collombet, E. Heard, Gene regulation in time and space during X-chromosome inactivation. *Nat. Rev. Mol. Cell Biol.* **23**, 231–249 (2022).

25. J. B. Berletch, W. Ma, F. Yang, J. Shendure, W. S. Noble, C. M. Disteche, X. Deng, Escape from X inactivation varies in mouse tissues. *PLOS Genet.* **11**, e1005079 (2015).

26. L. Carrel, H. F. Willard, Heterogeneous gene expression from the inactive X chromosome: An X-linked gene that escapes X inactivation in some human cell lines but is inactivated in others. *Proc. Natl. Acad. Sci. U.S.A.* **96**, 7364–7369 (1999).

27. F. Yang, T. Babak, J. Shendure, C. M. Disteche, Global survey of escape from X inactivation by RNA-sequencing in mouse. *Genome Res.* **20**, 614–622 (2010).

28. H. Wu, J. Luo, H. Yu, A. Rattner, A. Mo, Y. Wang, P. M. Smallwood, B. Erlanger, S. J. Wheelan, J. Nathans, Cellular resolution maps of X chromosome inactivation: Implications for neural development, function, and disease. *Neuron* **81**, 103–119 (2014).

29. J. B. Berletch, W. Ma, F. Yang, J. Shendure, W. S. Noble, C. M. Disteche, X. Deng, Identification of genes escaping X inactivation by allelic expression analysis in a novel hybrid mouse model. *Data Brief* **5**, 761–769 (2015).

30. G. Csankovszki, B. Panning, B. Bates, J. R. Pehrson, R. Jaenisch, Conditional deletion of *Xist* disrupts histone macroH2A localization but not maintenance of X inactivation. *Nat. Genet.* **22**, 323–324 (1999).

31. F. A. Wolf, P. Angerer, F. J. Theis, SCANPY: Large-scale single-cell gene expression data analysis. *Genome Biol.* **19**, 15 (2018).

32. Z. Yao, C. T. J. van Velthoven, T. N. Nguyen, J. Goldy, A. E. Sedeno-Cortes, F. Baftizadeh, D. Bertagnolli, T. Casper, M. Chiang, K. Crichton, S. L. Ding, O. Fong, E. Garren, A. Glandon, N. W. Gouwens, J. Gray, L. T. Graybuck, M. J. Hawrylycz, D. Hirschstein, M. Kroll, K. Lathia, C. Lee, B. Levi, D. McMillen, S. Mok, T. Pham, Q. Ren, C. Rimorin, N. Shapovalova, J. Sulc, S. M. Sunkin, M. Tieu, A. Torkelson, H. Tung, K. Ward, N. Dee, K. A. Smith, B. Tasic, H. Zeng, A taxonomy of transcriptomic cell types across the isocortex and hippocampal formation. *Cell* **184**, 3222–3241.e26 (2021).

33. K. H. Hajdarovic, D. Yu, L. A. Hassell, S. Evans, S. Packer, N. Neretti, A. E. Webb, Single-cell analysis of the aging female mouse hypothalamus. *Nat. Aging* **2**, 662–678 (2022).

34. A. Zeisel, A. B. Munoz-Manchado, S. Codeluppi, P. Lonnerberg, G. La Manno, A. Jureus, S. Marques, H. Munguba, L. He, C. Betsholtz, C. Rolny, G. Castelo-Branco, J. Hjerling-Leffler, S. Linnarsson, Brain structure. Cell types in the mouse cortex and hippocampus revealed by single-cell RNA-seq. *Science* **347**, 1138–1142 (2015).

35. B. Tasic, Z. Yao, L. T. Graybuck, K. A. Smith, T. N. Nguyen, D. Bertagnolli, J. Goldy, E. Garren, M. N. Economo, S. Viswanathan, O. Penn, T. Bakken, V. Menon, J. Miller, O. Fong, K. E. Hirokawa, K. Lathia, C. Rimorin, M. Tieu, R. Larsen, T. Casper, E. Barkan, M. Kroll, S. Parry, N. V. Shapovalova, D. Hirschstein, J. Pendergraft, H. A. Sullivan, T. K. Kim, A. Szafer, N. Dee, P. Groblewski, I. Wickersham, A. Cetin, J. A. Harris, B. P. Levi, S. M. Sunkin, L. Madisen, T. L. Daigle, L. Looger, A. Bernard, J. Phillips, E. Lein, M. Hawrylycz, K. Svoboda, A. R. Jones, C. Koch, H. Zeng, Shared and distinct transcriptomic cell types across neocortical areas. *Nature* **563**, 72–78 (2018).

36. B. Muzellec, M. Telenczuk, V. Cabeli, M. Andreux, PyDESeq2: A python package for bulk RNA-seq differential expression analysis. *Bioinformatics* **39**, btad547 (2023).

37. I. M. P. Badia, J. Velez Santiago, J. Braunger, C. Geiss, D. Dimitrov, S. Muller-Dott, P. Taus, A. Dugourd, C. H. Holland, R. O. Ramirez Flores, J. Saez-Rodriguez, decoupleR: Ensemble of computational methods to infer biological activities from omics data. *Bioinform. Adv.* **2**, vbac016 (2022).

38. M. Ximerakis, S. L. Lipnick, B. T. Innes, S. K. Simmons, X. Adiconis, D. Dionne, B. A. Mayweather, L. Nguyen, Z. Niziolek, C. Ozek, V. L. Butty, R. Isserlin, S. M. Buchanan, S. S. Levine, A. Regev, G. D. Bader, J. Z. Levin, L. L. Rubin, Single-cell transcriptomic profiling of the aging mouse brain. *Nat. Neurosci.* **22**, 1696–1708 (2019).

39. L. Kolberg, U. Raudvere, I. Kuzmin, P. Adler, J. Vilo, H. Peterson, g:Profiler-interoperable web service for functional enrichment analysis and gene identifier mapping (2023 update). *Nucleic Acids Res.* **51**, W207–W212 (2023).

40. U. Zechner, M. Wilda, H. Kehrer-Sawatzki, W. Vogel, R. Fundele, H. Hameister, A high density of X-linked genes for general cognitive ability: A run-away process shaping human evolution? *Trends Genet.* **17**, 697–701 (2001).

41. N. K. Hussain, G. M. Thomas, J. Luo, R. L. Huganir, Regulation of AMPA receptor subunit GluA1 surface expression by PAK3 phosphorylation. *Proc. Natl. Acad. Sci. U.S.A.* **112**, E5883–E5890 (2015).

42. W. Ba, J. van der Raadt, N. Nadif Kasri, Rho GTPase signaling at the synapse: Implications for intellectual disability. *Exp. Cell Res.* **319**, 2368–2374 (2013).

43. T. W. Chapman, R. A. Hill, Myelin plasticity in adulthood and aging. *Neurosci. Lett.* **715**, 134645 (2020).

44. E. Bagdatlioglu, P. Porcari, E. Greally, A. M. Blamire, V. W. Straub, Cognitive impairment appears progressive in the mdx mouse. *Neuromuscul. Disord.* **30**, 368–388 (2020).

45. C. Pascual-Morena, I. Cavero-Redondo, I. Sequi-Dominguez, E. Rodriguez-Gutierrez, M. E. Visier-Alfonso, V. Martinez-Vizcaino, Intelligence quotient-genotype association in dystrophinopathies: A systematic review and meta-analysis. *Neuropathol. Appl. Neurobiol.* **49**, e12914 (2023).

46. Y. Lou, X. Shi, G. Su, Y. Guo, L. Gao, Y. Wang, P. Miao, J. Feng, Hemizygous splicing variant in CNKSR2 results in X-linked intellectual developmental disorder. *Mol. Genet. Genomic Med.* **12**, e2389 (2024).

47. U. Aypar, E. C. Wirrell, N. L. Hoppman, CNKSR2 deletions: A novel cause of X-linked intellectual disability and seizures. *Am. J. Med. Genet. A* **167**, 1668–1670 (2015).

48. K. M. Allen, J. G. Gleeson, S. Bagrodia, M. W. Partington, J. C. MacMillan, R. A. Cerione, J. C. Mulley, C. A. Walsh, PAK3 mutation in nonsyndromic X-linked mental retardation. *Nat. Genet.* **20**, 25–30 (1998).

49. G. Pascolini, F. Gaudioso, C. Passarelli, A. Novelli, N. Di Giosaffatte, S. Majore, P. Grammatico, Clinical and molecular aspects of the neurodevelopmental disorder associated with PAK3 perturbation. *J. Mol. Neurosci.* **71**, 2474–2481 (2021).

50. D. Caudal, V. Francois, A. Lafoux, M. Ledevin, I. Anegon, C. Le Guiner, T. Larcher, C. Huchet, Characterization of brain dystrophins absence and impact in dystrophin-deficient Dmdmdx rat model. *PLOS ONE* **15**, e0230083 (2020).

51. H. Ito, R. Morishita, M. Noda, T. Ishiguro, M. Nishikawa, K. I. Nagata, The synaptic scaffolding protein CNKSR2 interacts with CYTH2 to mediate hippocampal granule cell development. *J. Biol. Chem.* **297**, 101427 (2021).

52. A. Dubos, G. Combeau, Y. Bernardinelli, J. V. Barnier, O. Hartley, H. Gaertner, B. Boda, D. Muller, Alteration of synaptic network dynamics by the intellectual disability protein PAK3. *J. Neurosci.* **32**, 519–527 (2012).

53. B. Boda, S. Alberi, I. Nikonenko, R. Node-Langlois, P. Jourdain, M. Moosmayer, L. Parisi-Jourdain, D. Muller, The mental retardation protein PAK3 contributes to synapse formation and plasticity in hippocampus. *J. Neurosci.* **24**, 10816–10825 (2004).

54. J. Meng, Y. Meng, A. Hanna, C. Janus, Z. Jia, Abnormal long-lasting synaptic plasticity and cognition in mice lacking the mental retardation gene Pak3. *J. Neurosci.* **25**, 6641–6650 (2005).

55. X. Li, V. Giri, Y. Cui, M. Yin, Z. Xian, J. Li, LncRNA FTX inhibits hippocampal neuron apoptosis by regulating miR-21-5p/SOX7 axis in a rat model of temporal lobe epilepsy. *Biochem. Biophys. Res. Commun.* **512**, 79–86 (2019).

56. G. Zhang, Y. Gao, L. Jiang, Y. Zhang, LncRNA FTX inhibits ferroptosis of hippocampal neurons displaying epileptiform discharges in vitro through the miR-142-5p/GABPB1 axis. *Neuroscience* **526**, 48–60 (2023).

57. C. Chureau, S. Chantalat, A. Romito, A. Galvani, L. Duret, P. Avner, C. Rougeulle, *Ftx* is a non-coding RNA which affects *Xist* expression and chromatin structure within the X-inactivation center region. *Hum. Mol. Genet.* **20**, 705–718 (2011).

58. H. B. Werner, E. M. Kramer-Albers, N. Strenzke, G. Saher, S. Tenzer, Y. Ohno-Iwashita, P. De Monasterio-Schrader, W. Mobius, T. Moser, I. R. Griffiths, K. A. Nave, A critical role for the cholesterol-associated proteolipids PLP and M6B in myelination of the central nervous system. *Glia* **61**, 567–586 (2013).

59. M. J. Osorio, S. A. Goldman, Neurogenetics of pelizaeus-merzbacher disease. *Handb. Clin. Neurol.* **148**, 701–722 (2018).

60. P. Aguilera, A. J. Lopez-Contreras, ATRX, a guardian of chromatin. *Trends Genet.* **39**, 505–519 (2023).

61. J. Shin, M. C. Wallingford, J. Gallant, C. Marcho, B. Jiao, M. Byron, M. Bossenz, J. B. Lawrence, S. N. Jones, J. Mager, I. Bach, RLIM is dispensable for X-chromosome inactivation in the mouse embryonic epiblast. *Nature* **511**, 86–89 (2014).

62. O. Rosspopoff, E. Cazottes, C. Huret, A. Loda, A. J. Collier, M. Casanova, P. J. Rugg-Gunn, E. Heard, J. F. Ouimette, C. Rougeulle, Species-specific regulation of *XIST* by the *JPX*/*FTX* orthologs. *Nucleic Acids Res.* **51**, 2177–2194 (2023).

63. K. M. Choi, J. Y. Kim, Y. Kim, Distribution of the immunoreactivity for glycoprotein M6B in the neurogenic niche and reactive glia in the injury penumbra following traumatic brain injury in mice. *Exp. Neurobiol.* **22**, 277–282 (2013).

64. M. Klugmann, M. H. Schwab, A. Puhlhofer, A. Schneider, F. Zimmermann, I. R. Griffiths, K. A. Nave, Assembly of CNS myelin in the absence of proteolipid protein. *Neuron* **18**, 59–70 (1997).

65. S. G. Kaler, ATP7A-related copper transport diseases—Emerging concepts and future trends. *Nat. Rev. Neurol.* **7**, 15–29 (2011).

66. S. Okano, Y. Makita, A. Miyamoto, G. Taketazu, K. Kimura, I. Fukuda, H. Tanaka, K. Yanagi, T. Kaname, GRIA3 p.Met661Thr variant in a female with developmental epileptic encephalopathy. *Hum. Genome Var.* **10**, 4 (2023).

67. J. Piard, J. H. Hu, P. M. Campeau, S. Rzonca, H. Van Esch, E. Vincent, M. Han, E. Rossignol, J. Castaneda, J. Chelly, C. Skinner, V. M. Kalscheuer, R. Wang, E. Lemyre, J. Kosinska, P. Stawinski, J. Bal, D. A. Hoffman, C. E. Schwartz, L. Van Maldergem, T. Wang, P. F. Worley, FRMPD4 mutations cause X-linked intellectual disability and disrupt dendritic spine morphogenesis. *Hum. Mol. Genet.* **27**, 589–600 (2018).

68. G. J. Ramakers, D. Wolfer, G. Rosenberger, K. Kuchenbecker, H. J. Kreienkamp, J. Prange-Kiel, G. Rune, K. Richter, K. Langnaese, S. Masneuf, M. R. Bosl, K. D. Fischer, H. J. Krugers, H. P. Lipp, E. van Galen, K. Kutsche, Dysregulation of Rho GTPases in the αPix/Arhgef6 mouse model of X-linked

intellectual disability is paralleled by impaired structural and synaptic plasticity and cognitive deficits. *Hum. Mol. Genet.* **21**, 268–286 (2012).

69. Z. Tumer, L. B. Moller, Menkes disease. *Eur. J. Hum. Genet.* **18**, 511–518 (2010).

70. U. Moog, K. Kutsche, "CASK disorders" in *GeneReviews*®, M. P. Adam, J. Feldman, G. M. Mirzaa, R. A. Pagon, S. E. Wallace, L. J. H. Bean, K. W. Gripp, A. Amemiya, Eds. (University of Washington, 1993).

71. M. Morleo, B. Franco, "Microphthalmia with linear skin defects syndrome" in *GeneReviews*®, M. P. Adam, J. Feldman, G. M. Mirzaa, R. A. Pagon, S. E. Wallace, L. J. H. Bean, K. W. Gripp, A. Amemiya, Eds. (University of Washington, 1993).

72. U. Hehr, G. Uyanik, L. Aigner, S. Couillard-Despres, J. Winkler, "DCX-related disorders" in *GeneReviews*®, M. P. Adam, J. Feldman, G. M. Mirzaa, R. A. Pagon, S. E. Wallace, L. J. H. Bean, K. W. Gripp, A. Amemiya, Eds. (University of Washington, 1993).

73. B. Jia, L. Huang, Y. Chen, S. Liu, C. Chen, K. Xiong, L. Song, Y. Zhou, X. Yang, M. Zhong, A novel contiguous deletion involving NDP, MAOB and EFHC2 gene in a patient with familial Norrie disease: Bilateral blindness and leucocoria without other deficits. *J. Genet.* **96**, 1015–1020 (2017).

74. J. F. Quesada-Espinosa, L. Garzon-Lorenzo, J. M. Lezana-Rosales, M. J. Gomez-Rodriguez, M. T. Sanchez-Calvin, C. Palma-Milla, I. Gomez-Manjon, I. Hidalgo-Mayoral, R. Perez de la Fuente, A. Arteche-Lopez, M. I. Alvarez-Mora, A. Camacho-Salas, J. Cruz-Rojo, I. Lazaro-Rodriguez, M. Morales-Conejo, N. Nunez-Enamorado, A. Bustamante-Aragones, R. Simon de Las Heras, M. A. Gomez-Cano, P. Ramos-Gomez, O. Sierra-Tomillo, A. Juarez-Rufian, J. Gallego-Merlo, L. Rausell-Sanchez, M. Moreno-Garcia, J. Sanchez Del Pozo, First female with Allan-Herndon-Dudley syndrome and partial deletion of X-inactivation center. *Neurogenetics* **22**, 343–346 (2021).

75. B. Rinaldi, A. Bayat, L. G. Zachariassen, J. H. Sun, Y. H. Ge, D. Zhao, K. Bonde, L. H. Madsen, I. A. A. Awad, D. Bagiran, A. Sbeih, S. M. Shah, S. El-Sayed, S. M. Lyngby, M. G. Pedersen, C. Stenum-Berg, L. C. Walker, I. Krey, A. Delahaye-Duriez, L. T. Emrick, K. Sully, C. N. Murali, L. C. Burrage, J. A. Plaud Gonzalez, M. Parnes, J. Friedman, B. Isidor, J. Lefranc, S. Redon, D. Heron, C. Mignot, B.

Keren, M. Fradin, C. Dubourg, S. Mercier, T. Besnard, B. Cogne, W. Deb, C. Rivier, D. Milani, M. F. Bedeschi, C. Di Napoli, F. Grilli, P. Marchisio, S. Koudijs, D. Veenma, E. Argilli, S. A. Lynch, P. Y. B. Au, F. E. Ayala Valenzuela, C. Brown, D. Masser-Frye, M. Jones, L. Patron Romero, W. L. Li, E. Thorpe, L. Hecher, J. Johannsen, J. Denecke, V. McNiven, A. Szuto, E. Wakeling, V. Cruz, V. Sency, H. Wang, J. Piard, F. Kortum, T. Herget, T. Bierhals, A. Condell, B. Ben-Zeev, S. Kaur, J. Christodoulou, A. Piton, C. Zweier, C. Kraus, A. Micalizzi, M. Trivisano, N. Specchio, G. Lesca, R. S. Moller, Z. Tumer, M. Musgaard, B. Gerard, J. R. Lemke, Y. S. Shi, A. S. Kristensen, Gain-of-function and loss-of-function variants in GRIA3 lead to distinct neurodevelopmental phenotypes. *Brain* **147**, 1837–1855 (2024).

76. M. I. Boyle, C. Jespersgaard, K. Brondum-Nielsen, A. M. Bisgaard, Z. Tumer, Cornelia de Lange syndrome. *Clin. Genet.* **88**, 1–12 (2015).

77. A. A. Gholkar, S. Senese, Y. C. Lo, E. Vides, E. Contreras, E. Hodara, J. Capri, J. P. Whitelegge, J. Z. Torres, The X-linked-intellectual-disability-associated Ubiquitin ligase Mid2 interacts with astrin and regulates astrin levels to promote cell division. *Cell Rep.* **14**, 180–188 (2016).

78. J. C. Lieske, D. S. Milliner, L. Beara-Lasic, P. Harris, A. Cogal, E. Abrash, "Dent disease" in *GeneReviews®*, M. P. Adam, J. Feldman, G. M. Mirzaa, R. A. Pagon, S. E. Wallace, L. J. H. Bean, K. W. Gripp, A. Amemiya, Eds. (University of Washington, 1993).

79. V. M. Pravata, M. Omelkova, M. P. Stavridis, C. M. Desbiens, H. M. Stephen, D. J. Lefeber, J. Gecz, M. Gundogdu, K. Ounap, S. Joss, C. E. Schwartz, L. Wells, D. M. F. van Aalten, An intellectual disability syndrome with single-nucleotide variants in O-GlcNAc transferase. *Eur. J. Hum. Genet.* **28**, 706–714 (2020).

80. C. Castillon, L. Gonzalez, F. Domenichini, S. Guyon, K. Da Silva, C. Durand, P. Lestaevel, C. Vaillend, S. Laroche, J. V. Barnier, R. Poirier, The intellectual disability PAK3 R67C mutation impacts cognitive functions and adult hippocampal neurogenesis. *Hum. Mol. Genet.* **29**, 1950–1968 (2020).

81. N. Sahajpal, C. Ziats, A. Chaubey, B. R. DuPont, F. Abidi, C. E. Schwartz, R. E. Stevenson, Clinical findings in individuals with duplication of genes associated with X-linked intellectual disability. *Clin. Genet.* **105**, 173–184 (2024).

82. R. C. Rogers, F. E. Abidi, "RPS6KA3-related intellectual disability" in *GeneReviews®*, M. P. Adam, J. Feldman, G. M. Mirzaa, R. A. Pagon, S. E. Wallace, L. J. H. Bean, K. W. Gripp, A. Amemiya, Eds. (University of Washington, 1993).

83. B. Poreau, F. Ramond, R. Harbuz, V. Satre, C. Barro, C. Vettier, V. Adouard, J. Thevenon, P. S. Jouk, C. Coutton, R. Touraine, K. Dieterich, Xq22.3q23 microdeletion harboring TMEM164 and AMMECR1 genes: Two case reports confirming a recognizable phenotype with short stature, midface hypoplasia, intellectual delay, and elliptocytosis. *Am. J. Med. Genet. A* **179**, 650–654 (2019).

84. S. Bassani, L. A. Cingolani, P. Valnegri, A. Folci, J. Zapata, A. Gianfelice, C. Sala, Y. Goda, M. Passafaro, The X-linked intellectual disability protein TSPAN7 regulates excitatory synapse development and AMPAR trafficking. *Neuron* **73**, 1143–1158 (2012).

85. Y. Huang, S. Huang, S. M. Lam, Z. Liu, G. Shui, Y. Q. Zhang, Acsl, the *Drosophila* ortholog of intellectual-disability-related ACSL4, inhibits synaptic growth by altered lipids. *J. Cell Sci.* **129**, 4034–4045 (2016).

86. A. Dubos, A. Castells-Nobau, H. Meziane, M. A. Oortveld, X. Houbaert, G. Iacono, C. Martin, C. Mittelhaeuser, V. Lalanne, J. M. Kramer, A. Bhukel, C. Quentin, J. Slabbert, P. Verstreken, S. J. Sigrist, N. Messaddeq, M. C. Birling, M. Selloum, H. G. Stunnenberg, Y. Humeau, A. Schenck, Y. Herault, Conditional depletion of intellectual disability and Parkinsonism candidate gene ATP6AP2 in fly and mouse induces cognitive impairment and neurodegeneration. *Hum. Mol. Genet.* **24**, 6736–6755 (2015).

87. R. E. Stevenson, "Alpha-thalassemia X-linked intellectual disability syndrome" in *GeneReviews®*, M. P. Adam, J. Feldman, G. M. Mirzaa, R. A. Pagon, S. E. Wallace, L. J. H. Bean, K. W. Gripp, A. Amemiya, Eds. (University of Washington, 1993).

88. A. Shukla, K. M. Girisha, P. H. Somashekar, S. Nampoothiri, R. McClellan, H. J. Vernon, Variants in the transcriptional corepressor BCORL1 are associated with an X-linked disorder of intellectual disability, dysmorphic features, and behavioral abnormalities. *Am. J. Med. Genet. A* **179**, 870–874 (2019).

89. N. D. Rendtorff, H. G. Karstensen, M. Lodahl, J. Tolmie, C. McWilliam, M. Bak, N. Tommerup, L. Nazaryan-Petersen, H. Kunst, M. Wong, S. Joss, V. Carelli, L. Tranebjaerg, Identification and analysis of deletion breakpoints in four Mohr-Tranebjaerg syndrome (MTS) patients. *Sci. Rep.* **12**, 14959 (2022).

90. S. Liang, N. Jiang, S. Li, X. Jiang, D. Yu, A maternally inherited 8.05 Mb Xq21 deletion associated with choroideremia, deafness, and mental retardation syndrome in a male patient. *Mol. Cytogenet.* **10**, 23 (2017).

91. L. Snijders Blok, E. Madsen, J. Juusola, C. Gilissen, D. Baralle, M. R. Reijnders, H. Venselaar, C. Helsmoortel, M. T. Cho, A. Hoischen, L. E. Vissers, T. S. Koemans, W. Wissink-Lindhout, E. E. Eichler, C. Romano, H. Van Esch, C. Stumpel, M. Vreeburg, E. Smeets, K. Oberndorff, B. W. van Bon, M. Shaw, J. Gecz, E. Haan, M. Bienek, C. Jensen, B. L. Loeys, A. Van Dijck, A. M. Innes, H. Racher, S. Vermeer, N. Di Donato, A. Rump, K. Tatton-Brown, M. J. Parker, A. Henderson, S. A. Lynch, A. Fryer, A. Ross, P. Vasudevan, U. Kini, R. Newbury-Ecob, K. Chandler, A. Male, D. D. D. Study, S. Dijkstra, J. Schieving, J. Giltay, K. L. van Gassen, J. Schuurs-Hoeijmakers, P. L. Tan, I. Pediaditakis, S. A. Haas, K. Retterer, P. Reed, K. G. Monaghan, E. Haverfield, M. Natowicz, A. Myers, M. C. Kruer, Q. Stein, K. A. Strauss, K. W. Brigatti, K. Keating, B. K. Burton, K. H. Kim, J. Charrow, J. Norman, A. Foster-Barber, A. D. Kline, A. Kimball, E. Zackai, M. Harr, J. Fox, J. McLaughlin, K. Lindstrom, K. M. Haude, K. van Roozendaal, H. Brunner, W. K. Chung, R. F. Kooy, R. Pfundt, V. Kalscheuer, S. G. Mehta, N. Katsanis, T. Kleefstra, Mutations in DDX3X are a common cause of unexplained intellectual disability with gender-specific effects on Wnt signaling. *Am. J. Hum. Genet.* **97**, 343–352 (2015).

92. A. Mir, Y. Song, H. Lee, H. Khanahmad, E. Khorram, J. Nasiri, M. A. Tabatabaiefar, Whole exome sequencing revealed variants in four genes underlying X-linked intellectual disability in four Iranian families: Novel deleterious variants and clinical features with the review of literature. *BMC Med. Genomics* **16**, 239 (2023).

93. S. L. Chiu, G. H. Diering, B. Ye, K. Takamiya, C. M. Chen, Y. Jiang, T. Niranjan, C. E. Schwartz, T. Wang, R. L. Huganir, GRASP1 regulates synaptic plasticity and learning through endosomal recycling of AMPA receptors. *Neuron* **93**, 1405–1419.e8 (2017).

94. N. S. Levy, G. K. E. Umanah, E. J. Rogers, R. Jada, O. Lache, A. P. Levy, IQSEC2-associated intellectual disability and autism. *Int. J. Mol. Sci.* **20**, 3038 (2019).

95. R. S. D’Souza, L. Law, *Danon Disease* (StatPearls, 2024).

96. T. Brunet, K. McWalter, K. Mayerhanser, G. M. Anbouba, A. Armstrong-Javors, I. Bader, E. Baugh, A. Begtrup, C. P. Bupp, B. L. Callewaert, A. Cereda, M. A. Cousin, J. C. Del Rey Jimenez, L. Demmer, N. R. Dsouza, N. Fleischer, R. H. Gavrilova, S. Ghate, E. Graf, A. Green, S. R. Green, M. Iascone, A. Kdissa, D. Klee, E. W. Klee, E. Lancaster, K. Lindstrom, J. A. Mayr, M. McEntagart, N. J. L. Meeks, D. Mittag, H. Moore, A. K. Olsen, D. Ortiz, G. Parsons, L. D. M. Pena, R. E. Person, S. Punj, G. A. Ramos-Rivera, M. J. G. Sacoto, G. Bradley Schaefer, R. E. Schnur, T. M. Scott, D. A. Scott, C. R. Serbinski, V. Shashi, V. M. Siu, B. F. Stadheim, J. A. Sullivan, J. Svantnerova, L. Velsher, D. S. Wargowski, I. M. Wentzensen, D. Wieczorek, J. Winkelmann, P. Yap, M. Zech, M. T. Zimmermann, T. Meitinger, F. Distelmaier, M. Wagner, Defining the genotypic and phenotypic spectrum of X-linked MSL3-related disorder. *Genet. Med.* **23**, 384–395 (2021).

97. Y. Wu, G. J. Lyon, NAA10-related syndrome. *Exp. Mol. Med.* **50**, 1–10 (2018).

98. T. A. Nguyen, A. W. Lehr, K. W. Roche, Neuroligins and neurodevelopmental disorders: X-linked genetics. *Front. Synaptic. Neurosci.* **12**, 33 (2020).

99. U. Lichter-Konecki, L. Caldovic, H. Morizono, K. Simpson, N. Ah Mew, E. MacLeod, “Ornithine transcarbamylase deficiency” in *GeneReviews®*, M. P. Adam, J. Feldman, G. M. Mirzaa, R. A. Pagon, S. E. Wallace, L. J. H. Bean, K. W. Gripp, A. Amemiya, Eds. (University of Washington, 1993).

100. K. Saida, T. Fukuda, D. A. Scott, T. Sengoku, K. Ogata, A. Nicosia, A. Hernandez-Garcia, S. R. Lalani, M. S. Azamian, H. Streff, P. Liu, H. Dai, T. Mizuguchi, S. Miyatake, M. Asahina, T. Ogata, N. Miyake, N. Matsumoto, OTUD5 variants associated with X-linked intellectual disability and congenital malformation. *Front. Cell. Dev. Biol.* **9**, 631428 (2021).

101. Z. Liu, B. Xin, I. N. Smith, V. Sency, J. Szekely, A. Alkelai, A. Shuldiner, S. Efthymiou, F. Rajabi, S. Coury, C. A. Brownstein, S. Rudnik-Schoneborn, A. L. Bruel, J. Thevenon, S. Zeidler, P. Jayakar, A. Schmidt, K. Cremer, H. Engels, S. O. Peters, M. S. Zaki, R. Duan, C. Zhu, Y. Xu, C. Gao, T. Sepulveda-Morales, R. Maroofian, I. A. Alkhawaja, M. Khawaja, H. Alhalasah, H. Houlden, J. A. Madden, V. Turchetti, D. Marafi, P. B. Agrawal, U. Schatz, A. Rotenberg, J. Rotenberg, G. M. S. Mancini, S. Bakhtiari, M. Kruer, I. Thiffault, S. Hirsch, M. Hempel, L. G. Stuhn, T. B. Haack, J. E. Posey, J. R.

Lupski, H. Lee, N. B. Sarn, C. Eng, C. Gonzaga-Jauregui, B. Zhang, H. Wang, Hemizygous variants in protein phosphatase 1 regulatory subunit 3F (PPP1R3F) are associated with a neurodevelopmental disorder characterized by developmental delay, intellectual disability and autistic features. *Hum. Mol. Genet.* **32**, 2981–2995 (2023).

102. H. Hu, S. A. Haas, J. Chelly, H. Van Esch, M. Raynaud, A. P. de Brouwer, S. Weinert, G. Froyen, S. G. Frints, F. Laumonnier, T. Zemojtel, M. I. Love, H. Richard, A. K. Emde, M. Bienek, C. Jensen, M. Hambrock, U. Fischer, C. Langnick, M. Feldkamp, W. Wissink-Lindhout, N. Lebrun, L. Castelnau, J. Rucci, R. Montjean, O. Dorseuil, P. Billuart, T. Stuhlmann, M. Shaw, M. A. Corbett, A. Gardner, S. Willis-Owen, C. Tan, K. L. Friend, S. Belet, K. E. van Roozendaal, M. Jimenez-Pocquet, M. P. Moizard, N. Ronce, R. Sun, S. O'Keeffe, R. Chenna, A. van Bommel, J. Goke, A. Hackett, M. Field, L. Christie, J. Boyle, E. Haan, J. Nelson, G. Turner, G. Baynam, G. Gillessen-Kaesbach, U. Muller, D. Steinberger, B. Budny, M. Badura-Stronka, A. Latos-Bielenska, L. B. Ousager, P. Wieacker, G. Rodriguez Criado, M. L. Bondeson, G. Anneren, A. Dufke, M. Cohen, L. Van Maldergem, C. Vincent-Delorme, B. Echenne, B. Simon-Bouy, T. Kleefstra, M. Willemsen, J. P. Fryns, K. Devriendt, R. Ullmann, M. Vingron, K. Wrogemann, T. F. Wienker, A. Tzschach, H. van Bokhoven, J. Gecz, T. J. Jentsch, W. Chen, H. H. Ropers, V. M. Kalscheuer, X-exome sequencing of 405 unresolved families identifies seven novel intellectual disability genes. *Mol. Psychiatry* **21**, 133–148 (2016).

103. C. Sarret, I. Oliver Petit, D. Tonduti, "Allan-Herndon-Dudley syndrome" in *GeneReviews*®, M. P. Adam, J. Feldman, G. M. Mirzaa, R. A. Pagon, S. E. Wallace, L. J. H. Bean, K. W. Gripp, A. Amemiya, Eds. (University of Washington, 1993).

104. C. Barba, I. Blumcke, M. R. Winawer, T. Hartlieb, H. C. Kang, L. Grisotto, M. Chipaux, C. G. Bien, B. Hermanovska, B. E. Porter, H. G. W. Lidov, V. Cetica, F. G. Woermann, J. A. Lopez-Rivera, P. D. Canoll, I. Mader, L. D'Incerti, S. Baldassari, E. Yang, A. Gaballa, H. Vogel, B. Straka, L. Macconi, T. Polster, G. A. Grant, L. Krskova, H. J. Shin, A. Ko, P. B. Crino, P. Krsek, J. H. Lee, D. Lal, S. Baulac, A. Poduri, R. Guerrini, SLC35A2 Study Group, Clinical features, neuropathology, and surgical outcome in patients with refractory epilepsy and brain somatic variants in the SLC35A2 gene. *Neurology* **100**, e528–e542 (2023).

105. W. Khayat, A. Hackett, M. Shaw, A. Ilie, T. Dudding-Byth, V. M. Kalscheuer, L. Christie, M. A. Corbett, J. Juusola, K. L. Friend, B. M. Kirmse, J. Gecz, M. Field, J. Orlowski, A recurrent missense variant in SLC9A7 causes nonsyndromic X-linked intellectual disability with alteration of Golgi acidification and aberrant glycosylation. *Hum. Mol. Genet.* **28**, 598–614 (2019).

106. F. Lopes, M. Barbosa, A. Ameur, G. Soares, J. de Sa, A. I. Dias, G. Oliveira, P. Cabral, T. Temudo, E. Calado, I. F. Cruz, J. P. Vieira, R. Oliveira, S. Esteves, S. Sauer, I. Jonasson, A. C. Syvanen, U. Gyllensten, D. Pinto, P. Maciel, Identification of novel genetic causes of Rett syndrome-like phenotypes. *J. Med. Genet.* **53**, 190–199 (2016).

107. Y. R. Lee, M. G. Thomas, A. Roychaudhury, C. Skinner, G. Maconachie, M. Crosier, H. Horak, C. S. Constantinescu, T. I. Choi, J. J. Kyung, T. Wang, B. Ku, B. N. Chodirker, M. F. Hammer, I. Gottlob, W. H. J. Norton, A. E. Chudley, C. E. Schwartz, C. H. Kim, Eye movement defects in KO zebrafish reveals *SRPK3* as a causative gene for an X-linked intellectual disability. *Res. Sq.* (2023).

108. R. Kumar, M. A. Corbett, B. W. Van Bon, A. Gardner, J. A. Woenig, L. A. Jolly, E. Douglas, K. Friend, C. Tan, H. Van Esch, M. Holvoet, M. Raynaud, M. Field, M. Leffler, B. Budny, M. Wisniewska, M. Badura-Stronka, A. Latos-Bielenska, J. Batanian, J. A. Rosenfeld, L. Basel-Vanagaite, C. Jensen, M. Bienek, G. Froyen, R. Ullmann, H. Hu, M. I. Love, S. A. Haas, P. Stankiewicz, S. W. Cheung, A. Baxendale, J. Nicholl, E. M. Thompson, E. Haan, V. M. Kalscheuer, J. Gecz, Increased STAG2 dosage defines a novel cohesinopathy with intellectual disability and behavioral problems. *Hum. Mol. Genet.* **24**, 7171–7181 (2015).

109. D. Lehalle, P. Vabres, A. Sorlin, T. Bierhals, M. Avila, V. Carmignac, M. Chevarin, E. Torti, Y. Abe, T. Bartolomaeus, J. Clayton-Smith, B. Cogne, I. Cusco, L. Duplomb, E. De Bont, Y. Duffourd, F. Duijkers, O. Elpeleg, A. Fattal, D. Genevieve, M. J. Guillen Sacoto, A. Guimier, D. J. Harris, M. Hempel, B. Isidor, T. Jouan, P. Kuentz, E. Koshimizu, K. Lichtenbelt, V. Loik Ramey, M. Maik, S. Miyakate, Y. Murakami, L. Pasquier, H. Pedro, L. Simone, K. Sondergaard-Schatz, J. St-Onge, J. Thevenon, I. Valenzuela, R. Abou Jamra, K. van Gassen, M. M. van Haelst, S. van Koningsbruggen, E. Verdura, C. Whelan Habela, P. Zacher, J. B. Riviere, C. Thauvin-Robinet, J. Betschinger, L. Faivre, De novo mutations in the X-linked TFE3 gene cause intellectual disability with pigmentary mosaicism and storage disorder-like features. *J. Med. Genet.* **57**, 808–819 (2020).

110. C. Mignon-Ravix, P. Cacciagli, N. Choucair, C. Popovici, C. Missirian, M. Milh, A. Megarbane, T. Busa, S. Julia, N. Girard, C. Badens, S. Sigaudy, N. Philip, L. Villard, Intragenic rearrangements in X-linked intellectual deficiency: Results of a-CGH in a series of 54 patients and identification of TRPC5 and KLHL15 as potential XLID genes. *Am. J. Med. Genet. A* **164A**, 1991–1997 (2014).

111. N. Vasli, I. Ahmed, K. Mittal, M. Ohadi, A. Mikhailov, M. A. Rafiq, A. Bhatti, M. T. Carter, D. M. Andrade, M. Ayub, J. B. Vincent, P. John, Identification of a homozygous missense mutation in LRP2 and a hemizygous missense mutation in TSPYL2 in a family with mild intellectual disability. *Psychiatr. Genet.* **26**, 66–73 (2016).

112. L. A. Jolly, C. C. Homan, R. Jacob, S. Barry, J. Gecz, The UPF3B gene, implicated in intellectual disability, autism, ADHD and childhood onset schizophrenia regulates neural progenitor cell behaviour and neuronal outgrowth. *Hum. Mol. Genet.* **22**, 4673–4687 (2013).

113. S. Kury, J. Zhang, T. Besnard, A. Caro-Llopis, X. Zeng, S. M. Robert, S. S. Josiah, E. Kiziltug, A. S. Denomme-Pichon, B. Cogne, A. J. Kundishora, L. T. Hao, H. Li, R. E. Stevenson, R. J. Louie, W. Deb, E. Torti, V. Vignard, K. McWalter, F. L. Raymond, F. Rajabi, E. Ranza, D. Grozeva, S. A. Coury, X. Blanc, E. Brischoux-Boucher, B. Keren, K. Ounap, K. Reinson, P. Ilves, I. M. Wentzensen, E. E. Barr, S. H. Guihard, P. Charles, E. G. Seaby, K. G. Monaghan, M. Rio, Y. van Bever, M. van Slegtenhorst, W. K. Chung, A. Wilson, D. Quinquis, F. Breheret, K. Retterer, P. Lindenbaum, E. Scalais, L. Rhodes, K. Stouffs, E. M. Pereira, S. M. Berger, S. S. Milla, A. B. Jaykumar, M. H. Cobb, S. Panchagnula, P. Q. Duy, M. Vincent, S. Mercier, B. Gilbert-Dussardier, X. Le Guillou, S. Audebert-Bellanger, S. Odent, S. Schmitt, P. Boisseau, D. Bonneau, A. Toutain, E. Colin, L. Pasquier, R. Redon, A. Bouman, J. A. Rosenfeld, M. J. Friez, H. Perez-Pena, S. R. Akhtar Rizvi, S. Haider, S. E. Antonarakis, C. E. Schwartz, F. Martinez, S. Bezieau, K. T. Kahle, B. Isidor, Rare pathogenic variants in WNK3 cause X-linked intellectual disability. *Genet. Med.* **24**, 1941–1951 (2022).

114. M. Parisi, I. Glass, “Joubert syndrome” in *GeneReviews*®, M. P. Adam, J. Feldman, G. M. Mirzaa, R. A. Pagon, S. E. Wallace, L. J. H. Bean, K. W. Gripp, A. Amemiya, Eds. (University of Washington, 1993).

115. R. Singh, D. Samanta, *Pelizaeus-Merzbacher Disease* (StatPearls, 2024).

116. B. A. Strange, M. P. Witter, E. S. Lein, E. I. Moser, Functional organization of the hippocampal longitudinal axis. *Nat. Rev. Neurosci.* **15**, 655–669 (2014).

117. J. L. Bizon, T. C. Foster, G. E. Alexander, E. L. Glisky, Characterizing cognitive aging of working memory and executive function in animal models. *Front. Aging Neurosci.* **4**, 19 (2012).

118. L. Sun, Z. Wang, T. Lu, T. A. Manolio, A. D. Paterson, eXclusionarY: 10 years later, where are the sex chromosomes in GWASs? *Am. J. Hum. Genet.* **110**, 903–912 (2023).

119. B. Chen, R. V. Craiu, L. J. Strug, L. Sun, The X factor: A robust and powerful approach to X-chromosome-inclusive whole-genome association studies. *Genet. Epidemiol.* **45**, 694–709 (2021).

120. J. Le Borgne, L. Gomez, S. Heikkinen, N. Amin, S. Ahmad, S. H. Choi, J. Bis, B. Grenier-Boley, O. G. Rodriguez, L. Kleineidam, J. Young, K. P. Tripathi, L. Wang, A. Varma, S. van der Lee, V. Damotte, I. de Rojas, S. Palmal, EADB, GR@ACE, DEGESCO, EADI, GERAD, DemGene, FinnGen, ADGC, CHARGE, R. Lipton, E. Reiman, A. M. Kee, P. De Jager, W. Bush, S. Small, A. Levey, A. Saykin, T. Foroud, M. Albert, B. Hyman, R. Petersen, S. Younkin, M. Sano, T. Wisniewski, R. Vassar, J. Schneider, V. Henderson, E. Roberson, C. De Carli, F. L. Ferla, J. Brewer, R. Swerdlow, L. Van Eldik, K. Hamilton-Nelson, H. Paulson, A. Naj, O. Lopez, H. Chui, P. Crane, T. Grabowski, W. Kukull, S. Asthana, S. Craft, S. Strittmatter, C. Cruchaga, J. Leverenz, A. Goate, M Ilyas Kamboh, P. St George-Hyslop, O. Valladares, A. Kuzma, L. Cantwell, M. Riemenschneider, J. Morris, S. Slifer, C. Dalmasso, A. Castillo, F. Küçükali, O. Peters, A. Schneider, M. Dichgans, D. Rujescu, N. Scherbaum, J. Deckert, S. Riedel-Heller, L. Hausner, L. Molina-Porcel, E. Düzel, T. Grimmer, J. Wiltfang, S. Heilmann-Heimbach, S. Moebus, T. Tegos, N. Scarmeas, O. Dols-Icardo, F. Moreno, J. Pérez-Tur, M. J. Bullido, P. Pastor, R. Sánchez-Valle, V. Álvarez, M. Boada, P. García-González, R. Puerta, P. Mir, L. M. Real, G. Piñol-Ripoll, J. M. García-Alberca, J. L. Royo, E. Rodriguez-Rodriguez, H. Soininen, A. de Mendonça, S. Mehrabian, L. Traykov, J. Hort, M. Vyhnalek, J. Q. Thomassen, Y. A. L. Pijnenburg, H. Holstege, J. van Swieten, I. Ramakers, F. Verhey, P. Scheltens, C. Graff, G. Papenberg, V. Giedraitis, R. Ghidoni, V. Fernandez, P. G. Kehoe, R. Frikke-Schmidt, M. Tsolaki, P. Sánchez-Juan, K. Sleegers, M. Ingelsson, J. Haines, L. Farrer, R. Mayeux, L.-S. Wang, R. Sims, A. DeStefano, G. D. Schellenberg, S. Seshadri, P. Amouyel, J. Williams, W. van der Flier, A. Ramirez, M. Pericak-Vance, O. Andreassen, C. Van Duijn, M. Hiltunen, A. Ruiz, J. Dupuis, E. Martin, J.-C. Lambert, B. Kunkle, C. Bellenguez, X

chromosome-wide association study for Alzheimer's disease. medRxiv 2024.05.02.24306739 [Preprint] (2024). https://doi.org/10.1101/2024.05.02.24306739.

121. E. Simmonds, G. Leonenko, U. Yaman, E. Bellou, A. Myers, K. Morgan, K. Brookes, J. Hardy, D. Salih, V. Escott-Price, Chromosome X-wide association study in case control studies of pathologically confirmed Alzheimer's disease in a European population. *Transl. Psychiatry* **14**, 358 (2024).

122. M. E. Belloy, Y. Le Guen, I. Stewart, K. Williams, J. Herz, R. Sherva, R. Zhang, V. Merritt, M. S. Panizzon, R. L. Hauger, J. M. Gaziano, M. Logue, V. Napolioni, M. D. Greicius, Role of the X chromosome in alzheimer disease genetics. *JAMA Neurol.* **81**, 1032–1042 (2024).

123. M. F. Lyon, Gene action in the X-chromosome of the mouse (Mus musculus L.). *Nature* **190**, 372–373 (1961).

124. T. Jegu, E. Aeby, J. T. Lee, The X chromosome in space. *Nat. Rev. Genet.* **18**, 377–389 (2017).

125. J. T. Lee, M. S. Bartolomei, X-inactivation, imprinting, and long noncoding RNAs in health and disease. *Cell* **152**, 1308–1323 (2013).

126. R. M. Boumil, J. T. Lee, Forty years of decoding the silence in X-chromosome inactivation. *Hum. Mol. Genet.* **10**, 2225–2232 (2001).

127. R. M. Malcore, S. Kalantry, A comparative analysis of mouse imprinted and random X-chromosome inactivation. *Epigenomes* **8**, 8 (2024).

128. C. M. Disteche, J. B. Berletch, X-chromosome inactivation and escape. *J. Genet.* **94**, 591–599 (2015).

129. E. Heard, C. Rougeulle, Digging into X chromosome inactivation. *Science* **374**, 942–943 (2021).

130. L. Carrel, C. J. Brown, When the Lyon(ized chromosome) roars: Ongoing expression from an inactive X chromosome. *Philos. Trans. R. Soc. Lond. B Biol. Sci.* **372**, 20160355 (2017).

131. M. Wang, F. Lin, K. Xing, L. Liu, Random X-chromosome inactivation dynamics in vivo by single-cell RNA sequencing. *BMC Genomics* **18**, 90 (2017).

132. H. Mohammed, I. Hernando-Herraez, A. Savino, A. Scialdone, I. Macaulay, C. Mulas, T. Chandra, T. Voet, W. Dean, J. Nichols, J. C. Marioni, W. Reik, Single-cell landscape of transcriptional heterogeneity and cell fate decisions during mouse early gastrulation. *Cell Rep.* **20**, 1215–1228 (2017).

133. A. Keniry, M. E. Blewitt, Studying X chromosome inactivation in the single-cell genomic era. *Biochem. Soc. Trans.* **46**, 577–586 (2018).

134. G. Chen, J. P. Schell, J. A. Benitez, S. Petropoulos, M. Yilmaz, B. Reinius, Z. Alekseenko, L. Shi, E. Hedlund, F. Lanner, R. Sandberg, Q. Deng, Single-cell analyses of X Chromosome inactivation dynamics and pluripotency during differentiation. *Genome Res.* **26**, 1342–1354 (2016).

135. H. Marks, H. H. Kerstens, T. S. Barakat, E. Splinter, R. A. Dirks, G. van Mierlo, O. Joshi, S. Y. Wang, T. Babak, C. A. Albers, T. Kalkan, A. Smith, A. Jouneau, W. de Laat, J. Gribnau, H. G. Stunnenberg, Dynamics of gene silencing during X inactivation using allele-specific RNA-seq. *Genome Biol.* **16**, 149 (2015).

136. K. Wainer Katsir, M. Linial, Human genes escaping X-inactivation revealed by single cell expression data. *BMC Genomics* **20**, 201 (2019).

137. M. Garieri, G. Stamoulis, X. Blanc, E. Falconnet, P. Ribaux, C. Borel, F. Santoni, S. E. Antonarakis, Extensive cellular heterogeneity of X inactivation revealed by single-cell allele-specific expression in human fibroblasts. *Proc. Natl. Acad. Sci. U.S.A.* **115**, 13015–13020 (2018).

138. Y. Tomofuji, R. Edahiro, K. Sonehara, Y. Shirai, K. H. Kock, Q. S. Wang, S. Namba, J. Moody, Y. Ando, A. Suzuki, T. Yata, K. Ogawa, T. Naito, H. Namkoong, Q. X. Xuan Lin, E. V. Buyamin, L. M. Tan, R. Sonthalia, K. Y. Han, H. Tanaka, H. Lee, Asian Immune Diversity Atlas (AIDA) Network, Japan COVID-19 Task Force, Biobank Japan Project, T. Okuno, B. Liu, K. Matsuda, K. Fukunaga, H. Mochizuki, W. Y. Park, K. Yamamoto, C. C. Hon, J. W. Shin, S. Prabhakar, A. Kumanogoh, Y. Okada, Quantification of escape from X chromosome inactivation with single-cell omics data reveals heterogeneity across cell types and tissues. *Cell Genom.* **4**, 100625 (2024).

139. A. K. San Roman, A. K. Godfrey, H. Skaletsky, D. W. Bellott, A. F. Groff, H. L. Harris, L. V. Blanton, J. F. Hughes, L. Brown, S. Phou, A. Buscetta, P. Kruszka, N. Banks, A. Dutra, E. Pak, P. C.

Lasutschinkow, C. Keen, S. M. Davis, N. R. Tartaglia, C. Samango-Sprouse, M. Muenke, D. C. Page, The human inactive X chromosome modulates expression of the active X chromosome. *Cell Genom.* **3**, 100259 (2023).

140. R. Aspinall, Longevity and the immune response. *Biogerontology* **1**, 273–278 (2000).

141. C. Lopez-Lee, L. Kodama, L. Fan, M. Y. Wong, N. R. Foxe, L. Jiaz, F. Yu, P. Ye, J. Zhu, K. Norman, E. R. Torres, R. D. Kim, G. A. Mousa, D. Dubal, S. Liddelow, W. Luo, L. Gan, Sex chromosomes and gonads shape the sex-biased transcriptomic landscape in Tlr7-mediated demyelination during aging. bioRxiv 2023.09.19.558439 [Preprint] (2023). https://doi.org/10.1101/2023.09.19.558439.

142. Y. Itoh, L. C. Golden, N. Itoh, M. A. Matsukawa, E. Ren, V. Tse, A. P. Arnold, R. R. Voskuhl, The X-linked histone demethylase Kdm6a in CD4+ T lymphocytes modulates autoimmunity. *J. Clin. Invest.* **129**, 3852–3863 (2019).

143. Y. Yan, X. Wang, D. Chaput, M. K. Shin, Y. Koh, L. Gan, A. A. Pieper, J. A. Woo, D. E. Kang, X-linked ubiquitin-specific peptidase 11 increases tauopathy vulnerability in women. *Cell* **185**, 3913–3930.e19 (2022).

144. F. Xie, P. Liang, H. Fu, J. C. Zhang, J. Chen, Effects of normal aging on myelin sheath ultrastructures in the somatic sensorimotor system of rats. *Mol. Med. Rep.* **10**, 459–466 (2014).

145. A. Peters, C. Sethares, Is there remyelination during aging of the primate central nervous system? *J. Comp. Neurol.* **460**, 238–254 (2003).

146. E. C. Sams, Oligodendrocytes in the aging brain. *Neuronal Signal.* **5**, NS20210008 (2021).

147. A. L. Graciani, M. U. Gutierre, A. A. Coppi, R. M. Arida, R. C. Gutierre, Myelin, aging, and physical exercise. *Neurobiol. Aging* **127**, 70–81 (2023).

148. K. A. Phillips, C. M. Watson, A. Bearman, A. R. Knippenberg, J. Adams, C. Ross, S. D. Tardif, Age-related changes in myelin of axons of the corpus callosum and cognitive decline in common marmosets. *Am. J. Primatol.* **81**, e22949 (2019).

149. P. Bonifazi, M. Goldin, M. A. Picardo, I. Jorquera, A. Cattani, G. Bianconi, A. Represa, Y. Ben-Ari, R. Cossart, GABAergic hub neurons orchestrate synchrony in developing hippocampal networks. *Science* **326**, 1419–1424 (2009).

150. C. Lopez-Otin, M. A. Blasco, L. Partridge, M. Serrano, G. Kroemer, Hallmarks of aging: An expanding universe. *Cell* **186**, 243–278 (2023).

151. Y. Liu, L. Sinke, T. H. Jonkman, R. C. Slieker, BIOS Consortium, E. W. van Zwet, L. Daxinger, B. T. Heijmans, The inactive X chromosome accumulates widespread epigenetic variability with age. *Clin. Epigenetics* **15**, 135 (2023).

152. A. Grigoryan, J. Pospiech, S. Kramer, D. Lipka, T. Liehr, H. Geiger, H. Kimura, M. A. Mulaw, M. C. Florian, Attrition of X chromosome inactivation in aged hematopoietic stem cells. *Stem Cell Rep.* **16**, 708–716 (2021).

153. S. Li, J. B. Lund, K. Christensen, J. Baumbach, J. Mengel-From, T. Kruse, W. Li, A. Mohammadnejad, A. Pattie, R. E. Marioni, I. J. Deary, Q. Tan, Exploratory analysis of age and sex dependent DNA methylation patterns on the X-chromosome in whole blood samples. *Genome Med.* **12**, 39 (2020).

154. A. J. Sharp, E. Stathaki, E. Migliavacca, M. Brahmachary, S. B. Montgomery, Y. Dupre, S. E. Antonarakis, DNA methylation profiles of human active and inactive X chromosomes. *Genome Res.* **21**, 1592–1600 (2011).

155. W. Chang, Y. Zhao, D. Rayee, Q. Xie, M. Suzuki, D. Zheng, A. Cvekl, Dynamic changes in whole genome DNA methylation, chromatin and gene expression during mouse lens differentiation. *Epigenetics Chromatin* **16**, 4 (2023).

156. L. Giorgetti, B. R. Lajoie, A. C. Carter, M. Attia, Y. Zhan, J. Xu, C. J. Chen, N. Kaplan, H. Y. Chang, E. Heard, J. Dekker, Structural organization of the inactive X chromosome in the mouse. *Nature* **535**, 575–579 (2016).

157. J. G. Herndon, The grandmother effect: Implications for studies on aging and cognition. *Gerontology* **56**, 73–79 (2010).

158. S. Nattrass, D. P. Croft, S. Ellis, M. A. Cant, M. N. Weiss, B. M. Wright, E. Stredulinsky, T. Doniol-Valcroze, J. K. B. Ford, K. C. Balcomb, D. W. Franks, Postreproductive killer whale grandmothers improve the survival of their grandoffspring. *Proc. Natl. Acad. Sci. U.S.A.* **116**, 26669–26673 (2019).

159. K. Hawkes, Grandmothers and the evolution of human longevity. *Am. J. Hum. Biol.* **15**, 380–400 (2003).

160. K. Hawkes, The grandmother effect. *Nature* **428**, 128–129 (2004).

161. J. S. Peccei, A critique of the grandmother hypotheses: Old and new. *Am. J. Hum. Biol.* **13**, 434–452 (2001).

162. K. A. Wareham, M. F. Lyon, P. H. Glenister, E. D. Williams, Age related reactivation of an X-linked gene. *Nature* **327**, 725–727 (1987).

163. B. P. Balaton, O. Fornes, W. W. Wasserman, C. J. Brown, Cross-species examination of X-chromosome inactivation highlights domains of escape from silencing. *Epigenetics Chromatin* **14**, 12 (2021).

164. H. Fang, C. M. Disteche, J. B. Berletch, X inactivation and escape: Epigenetic and structural features. *Front. Cell Dev. Biol.* **7**, 219 (2019).

165. B. R. Migeon, An overview of X inactivation based on species differences. *Semin. Cell Dev. Biol.* **56**, 111–116 (2016).

166. A. J. Sandweiss, V. L. Brandt, H. Y. Zoghbi, Advances in understanding of Rett syndrome and MECP2 duplication syndrome: Prospects for future therapies. *Lancet Neurol.* **19**, 689–698 (2020).

167. L. M. Lombardi, S. A. Baker, H. Y. Zoghbi, MECP2 disorders: From the clinic to mice and back. *J. Clin. Invest.* **125**, 2914–2923 (2015).

168. N. B. Grimm, J. T. Lee, Selective Xi reactivation and alternative methods to restore MECP2 function in Rett syndrome. *Trends Genet.* **38**, 920–943 (2022).

169. A. Zito, J. T. Lee, Variable expression of MECP2, CDKL5, and FMR1 in the human brain: Implications for gene restorative therapies. *Proc. Natl. Acad. Sci. U.S.A.* **121**, e2312757121 (2024).

170. R. A. Neff, M. Wang, S. Vatansever, L. Guo, C. Ming, Q. Wang, E. Wang, E. Horgusluoglu-Moloch, W. M. Song, A. Li, E. L. Castranio, J. Tcw, L. Ho, A. Goate, V. Fossati, S. Noggle, S. Gandy, M. E. Ehrlich, P. Katsel, E. Schadt, D. Cai, K. J. Brennand, V. Haroutunian, B. Zhang, Molecular subtyping of Alzheimer's disease using RNA sequencing data reveals novel mechanisms and targets. *Sci. Adv.* **7**, eabb5398 (2021).

171. C. Xu, M. Prete, S. Webb, L. Jardine, B. J. Stewart, R. Hoo, P. He, K. B. Meyer, S. A. Teichmann, Automatic cell-type harmonization and integration across Human Cell Atlas datasets. *Cell* **186**, 5876–5891.e20 (2023).

172. C. Dominguez Conde, C. Xu, L. B. Jarvis, D. B. Rainbow, S. B. Wells, T. Gomes, S. K. Howlett, O. Suchanek, K. Polanski, H. W. King, L. Mamanova, N. Huang, P. A. Szabo, L. Richardson, L. Bolt, E. S. Fasouli, K. T. Mahbubani, M. Prete, L. Tuck, N. Richoz, Z. K. Tuong, L. Campos, H. S. Mousa, E. J. Needham, S. Pritchard, T. Li, R. Elmentaite, J. Park, E. Rahmani, D. Chen, D. K. Menon, O. A. Bayraktar, L. K. James, K. B. Meyer, N. Yosef, M. R. Clatworthy, P. A. Sims, D. L. Farber, K. Saeb-Parsy, J. L. Jones, S. A. Teichmann, Cross-tissue immune cell analysis reveals tissue-specific features in humans. *Science* **376**, eabl5197 (2022).

173. E. Caglayan, Y. Liu, G. Konopka, Neuronal ambient RNA contamination causes misinterpreted and masked cell types in brain single-nuclei datasets. *Neuron* **110**, 4043–4056.e5 (2022).

174. S. L. Wolock, R. Lopez, A. M. Klein, Scrublet: Computational identification of cell doublets in single-cell transcriptomic data. *Cell Syst.* **8**, 281–291.e9 (2019).

175. S. Abdulai-Saiku, S. Gupta, D. Wang, A. J. Moreno, Y. Huang, D. Srivastava, B. Panning, D. B. Dubal, The maternal X chromosome impairs cognition and accelerates brain aging through epigenetic modulation in female mice. bioRxiv 2022.03.09.483691 [Preprint] (2022). https://doi.org/10.1101/2022.03.09.483691.

176. A. Ferraj, P. A. Audano, P. Balachandran, A. Czechanski, J. I. Flores, A. A. Radecki, V. Mosur, D. S. Gordon, I. A. Walawalkar, E. E. Eichler, L. G. Reinholdt, C. R. Beck, Resolution of structural variation in diverse mouse genomes reveals chromatin remodeling due to transposable elements. *Cell Genom.* **3**, 100291 (2023).

177. J. F. Degner, J. C. Marioni, A. A. Pai, J. K. Pickrell, E. Nkadori, Y. Gilad, J. K. Pritchard, Effect of read-mapping biases on detecting allele-specific expression from RNA-sequencing data. *Bioinformatics* **25**, 3207–3212 (2009).

178. M. Lewandoski, K. M. Wassarman, G. R. Martin, Zp3-cre, a transgenic mouse line for the activation or inactivation of loxP-flanked target genes specifically in the female germ line. *Curr. Biol.* **7**, 148–151 (1997).

179. U. Raudvere, L. Kolberg, I. Kuzmin, T. Arak, P. Adler, H. Peterson, J. Vilo, g:Profiler: A web server for functional enrichment analysis and conversions of gene lists (2019 update). *Nucleic Acids Res.* **47**, W191–W198 (2019).

180. M. Wang, A. Li, M. Sekiya, N. D. Beckmann, X. Quan, N. Schrode, M. B. Fernando, A. Yu, L. Zhu, J. Cao, L. Lyu, E. Horgusluoglu, Q. Wang, L. Guo, Y.-s. Wang, R. Neff, W.-m. Song, E. Wang, Q. Shen, X. Zhou, C. Ming, S.-M. Ho, S. Vatansever, H. Ü. Kaniskan, J. Jin, M.-M. Zhou, K. Ando, L. Ho, P. A. Slesinger, Z. Yue, J. Zhu, P. Katsel, S. Gandy, M. E. Ehrlich, V. Fossati, S. Noggle, D. Cai, V. Haroutunian, K. M. Iijima, E. Schadt, K. J. Brennand, B. Zhang, Transformative network modeling of multi-omics data reveals detailed circuits, key regulators, and potential therapeutics for Alzheimer’s disease. *Neuron* **109**, 257–272.e14 (2021).

181. M. Wang, N. D. Beckmann, P. Roussos, E. Wang, X. Zhou, Q. Wang, C. Ming, R. Neff, W. Ma, J. F. Fullard, M. E. Hauberg, J. Bendl, M. A. Peters, B. Logsdon, P. Wang, M. Mahajan, L. M. Mangravite, E. B. Dammer, D. M. Duong, J. J. Lah, N. T. Seyfried, A. I. Levey, J. D. Buxbaum, M. Ehrlich, S. Gandy, P. Katsel, V. Haroutunian, E. Schadt, B. Zhang, The Mount Sinai cohort of large-scale genomic, transcriptomic and proteomic data in Alzheimer’s disease. *Sci. Data* **5**, 180185 (2018).

182. M. I. Love, W. Huber, S. Anders, Moderated estimation of fold change and dispersion for RNA-seq data with DESeq2. *Genome Biol.* **15**, 550 (2014).

183. A. D. Edelstein, M. A. Tsuchida, N. Amodaj, H. Pinkard, R. D. Vale, N. Stuurman, Advanced methods of microscope control using μManager software. *J. Biol. Methods* **1**, e10 (2014).

184. J. Schindelin, I. Arganda-Carreras, E. Frise, V. Kaynig, M. Longair, T. Pietzsch, S. Preibisch, C. Rueden, S. Saalfeld, B. Schmid, J. Y. Tinevez, D. J. White, V. Hartenstein, K. Eliceiri, P. Tomancak, A. Cardona, Fiji: An open-source platform for biological-image analysis. *Nat. Methods* **9**, 676–682 (2012).

185. S. Schildge, C. Bohrer, K. Beck, C. Schachtrup, Isolation and culture of mouse cortical astrocytes. *J. Vis. Exp.*, 50079 (2013).

186. J. Schwieger, K. H. Esser, T. Lenarz, V. Scheper, Establishment of a long-term spiral ganglion neuron culture with reduced glial cell number: Effects of AraC on cell composition and neurons. *J. Neurosci. Methods* **268**, 106–116 (2016).

187. D. B. Dubal, L. Zhu, P. E. Sanchez, K. Worden, L. Broestl, E. Johnson, K. Ho, G. Q. Yu, D. Kim, A. Betourne, O. M. Kuro, E. Masliah, C. R. Abraham, L. Mucke, Life extension factor klotho prevents mortality and enhances cognition in hAPP transgenic mice. *J. Neurosci.* **35**, 2358–2371 (2015).

188. C. Park, O. Hahn, S. Gupta, A. J. Moreno, F. Marino, B. Kedir, D. Wang, S. A. Villeda, T. Wyss-Coray, D. B. Dubal, Platelet factors are induced by longevity factor klotho and enhance cognition in young and aging mice. *Nat. Aging* **3**, 1067–1078 (2023).

189. D. B. Dubal, J. S. Yokoyama, L. Zhu, L. Broestl, K. Worden, D. Wang, V. E. Sturm, D. Kim, E. Klein, G. Q. Yu, K. Ho, K. E. Eilertson, L. Yu, M. Kuro-o, P. L. De Jager, G. Coppola, G. W. Small, D. A. Bennett, J. H. Kramer, C. R. Abraham, B. L. Miller, L. Mucke, Life extension factor klotho enhances cognition. *Cell Rep.* **7**, 1065–1076 (2014).

190. J. Leon, A. J. Moreno, B. I. Garay, R. J. Chalkley, A. L. Burlingame, D. Wang, D. B. Dubal, Peripheral elevation of a klotho fragment enhances brain function and resilience in young, aging, and α-synuclein transgenic mice. *Cell Rep.* **20**, 1360–1371 (2017).

191. F. Dellu, W. Mayo, J. Cherkaoui, M. Le Moal, H. Simon, A two-trial memory task with automated recording: Study in young and aged rats. *Brain Res.* **588**, 132–139 (1992).

192. L. A. Goff, A. F. Groff, M. Sauvageau, Z. Trayes-Gibson, D. B. Sanchez-Gomez, M. Morse, R. D. Martin, L. E. Elcavage, S. C. Liapis, M. Gonzalez-Celeiro, O. Plana, E. Li, C. Gerhardinger, G. S. Tomassy, P. Arlotta, J. L. Rinn, Spatiotemporal expression and transcriptional perturbations by long noncoding RNAs in the mouse brain. *Proc. Natl. Acad. Sci. U.S.A.* **112**, 6855–6862 (2015).

193. G. Figlia, S. Muller, A. M. Hagenston, S. Kleber, M. Roiuk, J. P. Quast, N. Ten Bosch, D. Carvajal Ibanez, D. Mauceri, A. Martin-Villalba, A. A. Teleman, Brain-enriched RagB isoforms regulate the dynamics of mTORC1 activity through GATOR1 inhibition. *Nat. Cell Biol.* **24**, 1407–1421 (2022).

194. S. H. Jung, M. L. Brownlow, M. Pellegrini, R. Jankord, Divergence in Morris water maze-based cognitive performance under chronic stress is associated with the hippocampal whole transcriptomic modification in mice. *Front. Mol. Neurosci.* **10**, 275 (2017).

195. C. Montani, M. Ramos-Brossier, L. Ponzoni, L. Gritti, A. W. Cwetsch, D. Braida, Y. Saillour, B. Terragni, M. Mantegazza, M. Sala, C. Verpelli, P. Billuart, C. Sala, The X-linked intellectual disability protein IL1RAPL1 regulates dendrite complexity. *J. Neurosci.* **37**, 6606–6627 (2017).

196. C. Montani, L. Gritti, S. Beretta, C. Verpelli, C. Sala, The synaptic and neuronal functions of the X-linked intellectual disability protein interleukin-1 receptor accessory protein like 1 (IL1RAPL1). *Dev. Neurobiol.* **79**, 85–95 (2019).

197. D. Rujescu, E. M. Meisenzahl, S. Krejcova, I. Giegling, T. Zetzsche, M. Reiser, C. M. Born, H.-J. Möller, A. Veske, A. Gal, U. Finckh, Plexin B3 is genetically associated with verbal performance and white matter volume in human brain. *Mol. Psychiatry* **12**, 190–194 (2007).
